# Supplementary material for: A new molecular tool for detection of the highly invasive gecko, Hemidactylus frenatus
Source: PLoS One. 2026 Feb 4;21(2):e0338377. doi: 10.1371/journal.pone.0338377 (PMC12871987; doi:10.1371/journal.pone.0338377)
Supplement: S1 Table — Species identify determined through positive LAMP result and/or DNA barcoding. (DOCX) [file pone.0338377.s001.docx]

**S1 Supplementary Table.** Samples tested infield: n=100 scats, n=8 tissue samples. Species identification determined through positive LAMP result and/or DNA barcoding.

| **Sample**  **Number** | **Sample ID** | **Location** | **Type** | **In-field Time**  **(min)** | **In-field Temp (^o^C)** | **LAMP**  **Positive**  **(In Field)** | **Lab Time**  **(min)** | **Lab Temp**  **(^o^C)** | **LAMP**  **Positive**  **(In Lab)** | **PCR for**  **DNA barcode** | **ND2 DNA**  **Barcode**  **Obtained** | **Species Identity** |
| --- | --- | --- | --- | --- | --- | --- | --- | --- | --- | --- | --- | --- |
| 1 | BS10 | Karratha | Tissue | 11 | 84.3 | Yes | 9.75 | 84.7 | Yes |  |  | *Hemidactylus frenatus* |
| 2 | BS18 | Karratha | Tissue | 10.5 | 84.6 | Yes | 10.5 | 84.9 | Yes |  |  | *Hemidactylus frenatus* |
| 3 | BS41 | Batam | Tissue | 16 | 85.3 | Yes | 13.75 | 85.3 | Yes |  |  | *Hemidactylus frenatus* |
| 5 | Yard 2 shed | Dampier | Scat | 13 | 84.5 | Yes | 11.75 | 85 | Yes | Yes | Yes | *Hemidactylus frenatus* |
| 6 | Unknown 1 | Perth | Scat |  | - |  |  | - |  | Yes |  |  |
| 7 | C4156 | BWI | Scat |  | - |  |  | - |  | Yes | Yes | *Gehyra variegata* |
| 8 | C4163 | BWI | Scat |  | - |  |  | - |  | Yes | Yes | *Gehyra variegata* |
| 9 | C4165 | BWI | Scat |  | - |  |  | - |  | Yes |  |  |
| 10 | Yard 2 office | Dampier | Scat |  | - |  |  | - |  | Yes | Yes | *Gehyra sp.* |
| 11 | Yard 2 office | Dampier | Scat |  | - |  |  | - |  | Yes |  |  |
| 12 | BS8 | Karratha | Tissue | 7.5 | 84.8 | Yes | 10.25 | 84.7 | Yes |  |  | *Hemidactylus frenatus* |
| 13 | C4166 | BWI | Scat |  | - |  |  | - |  | Yes |  |  |
| 14 | C4167 | BWI | Scat |  | - |  |  | - |  | Yes |  |  |
| 15 | C4183 | BWI | Scat |  | - |  |  | - |  | Yes |  |  |
| 16 | DSB yard 1 office | Dampier | Scat |  | - | No | 16 | 84.6 | Yes |  |  | *Hemidactylus frenatus* |
| 17 | DSB yard 1 office | Dampier | Scat | 23 | 84.4 | Yes | 21.5 | 84.9 | Yes |  |  | *Hemidactylus frenatus* |
| 18 | BS44 | Batam | Tissue | 11.75 | 84.5 | Yes | 10.75 | 84.5 | Yes |  |  | *Hemidactylus frenatus* |
| 19 | C4162 | BWI | Scat |  | - |  |  | - |  | Yes |  |  |
| 20 | C4159 | BWI | Scat |  | - |  |  | - |  | Yes |  |  |
| 21 | C4181 | BWI | Scat |  | - |  |  | - |  | Yes |  |  |
| 22 | D11 | Dampier | Scat | 14.25 | 84.7 | Yes | 12 | 84.8 | Yes |  |  | *Hemidactylus frenatus* |
| 23 | D10 | Dampier | Scat | 15 | 84.5 | Yes | 13.75 | 84.8 | Yes | Yes | Yes | *Hemidactylus frenatus* |
| 24 | BS1 | Karratha | Tissue | 12 | 84.5 | Yes | 10 | 84.8 | Yes |  |  | *Hemidactylus frenatus* |
| 25 | TDSB-01 | Dampier | Scat |  | - | No | 23 | 85.19 | Yes |  |  | *Hemidactylus frenatus* |
| 26 | TDSB-04 | Dampier | Scat |  | 85 |  |  | - |  | Yes |  |  |
| 27 | DSB-10 | Dampier | Scat |  | 84.2 |  |  | - |  | Yes |  |  |
| 28 | DSB-13 | Dampier | Scat |  | 84.2 | No | 19.75 | 84.8 | Yes |  |  | *Hemidactylus frenatus* |
| 29 | C4168 | BWI | Scat |  | - |  |  | - |  | Yes | Yes | *Gehyra variegata* |
| 30 | BS43 | Batam | Tissue | 17.25 | 84.6 | Yes | 23.75 | 84.7 | Yes |  |  | *Hemidactylus frenatus* |
| 31 | 7 | Dampier | Scat | 15.5 | 84.2 | Yes | 16 | 85 | Yes | Yes | Yes | *Hemidactylus frenatus* |
| 32 | 8 | Dampier | Scat |  | - |  |  | - |  | Yes |  |  |
| 33 | 2 | Dampier | Scat | 12.75 | 84.6 | Yes | 12.25 | 85 | Yes | Yes | Yes | *Hemidactylus frenatus* |
| 34 | 3 | Dampier | Scat |  | - |  |  | - |  | Yes |  |  |
| 35 | C4158 | BWI | Scat |  | - |  |  | - |  | Yes |  |  |
| 36 | BS42 | Batam | Tissue | 10.25 | 84.7 | Yes | 9.5 | 85 | Yes |  |  | *Hemidactylus frenatus* |
| 37 | K-01 (fresh) | Karratha | Scat | 14.75 | 84.4 | Yes | 13.75 | 84.8 | Yes | Yes | Yes | *Hemidactylus frenatus* |
| 38 | K-02 (dry) | Karratha | Scat |  | 84.4 | No | 21.25 | 84.8 | Yes | Yes | Yes | *Hemidactylus frenatus* |
| 39 | K-03 (fresh) | Karratha | Scat |  | - |  |  | - |  | Yes | Yes | *Gehyra pilbara* |
| 40 | K-04 (dry) | Karratha | Scat | 13.25 | 85.7 | Yes | 12.25 | 84.9 | Yes | Yes | Yes | *Hemidactylus frenatus* |
| 41 | K-05 (dry small) | Karratha | Scat | 15.75 | 84.4 | Yes | 18.25 | 84.8 | Yes |  |  | *Hemidactylus frenatus* |
| 42 | K-06 (dry large) | Karratha | Scat | 18.25 | 84.6 | Yes | 13 | 85.5 | Yes |  |  | *Hemidactylus frenatus* |
| 43 | K-07 (fresh) | Karratha | Scat | 14.75 | 84.4 | Yes | 11.75 | 84.8 | Yes | Yes | Yes | *Hemidactylus frenatus* |
| 44 | K-08 (fresh) | Karratha | Scat | 15.5 | 84.5 | Yes | 15 | 84.8 | Yes | Yes | Yes | *Hemidactylus frenatus* |
| 45 | K-09 (dry) | Karratha | Scat |  | - |  |  | - |  | Yes |  |  |
| 46 | K-10 (very dry) | Karratha | Scat |  | - | No |  | - |  | Yes | Yes | *Hemidactylus frenatus* |
| 47 | K-11 (very dry) | Karratha | Scat |  | - |  |  | 84.8 |  | Yes |  |  |
| 48 | K-12 (very dry) | Karratha | Scat | 9 | - | No | 23.25 | 84.7 | Yes | Yes | Yes | *Hemidactylus frenatus* |
| 49 | K-13 (fresh) | Karratha | Scat | 13.5 | 84.6 | Yes | 15.5 | 84.6 | Yes |  |  | *Hemidactylus frenatus* |
| 50 | K-14 (fresh) | Karratha | Scat | 13.5 | 84.7 | Yes | 15 | 84.5 | Yes | Yes | Yes | *Hemidactylus frenatus* |
| 51 | k-15 (dry) | Karratha | Scat | 18.5 | 84.3 | Yes | 16.5 | 85 | Yes |  |  | *Hemidactylus frenatus* |
| 52 | K-16 (dry) | Karratha | Scat | 12.5 | 84.4 | Yes | 12.25 | 84.9 | Yes | Yes | Yes | *Hemidactylus frenatus* |
| 53 | K-17 (dry) | Karratha | Scat | 15 | 84.6 | Yes | 14.5 | 85 | Yes |  |  | *Hemidactylus frenatus* |
| 54 | DSB-05 (very dry) | Dampier | Scat |  | - |  |  | - |  | Yes |  |  |
| 55 | K-39 | Karratha | Scat | 13.5 | 84.5 | Yes | 13.5 | 84.8 | Yes | Yes | Yes | *Hemidactylus frenatus* |
| 56 | C4189 | BWI, Airport | Scat |  | - |  |  | - |  | Yes | Yes | *Heteronotia binoei* |
| 57 | C4187 | BWI, Airport | Scat |  | - |  |  | - |  | Yes |  |  |
| 58 | C4401 | BWI, Airport | Scat |  | - |  |  | - |  | Yes |  |  |
| 59 | C4198 | BWI, Airport | Scat |  | - |  |  | - |  | Yes |  |  |
| 60 | K-42 | Karratha | Scat | 19.75 | 84.4 | Yes | 14.25 | 85 | Yes | Yes | Yes | *Hemidactylus frenatus* |
| 61 | K-40 | Karratha | Scat | 19.5 | 84.2 | Yes | 0.25 | 84.6 | Yes | Yes | Yes | *Hemidactylus frenatus* |
| 62 | K-33 | Karratha | Scat | 13.5 | 84.5 | Yes | 13.5 | 84.5 | Yes | Yes | Yes | *Hemidactylus frenatus* |
| 63 | K-43 | Karratha | Scat |  | - | No |  | - |  | Yes | Yes | *Hemidactylus frenatus* |
| 64 | C4387 | BWI, abutment | Scat |  | - |  |  | - |  | Yes |  |  |
| 65 | C4449 | BWI, WAPET | Scat |  | - |  |  | - |  | Yes |  |  |
| 66 | C4406 | BWI, WAPET | Scat |  | - |  |  | - |  | Yes | Yes | *Cryptoblepharus plagiocephalus* |
| 67 | C4407 | BWI | Scat |  | - |  |  | - |  | Yes |  |  |
| 68 | C4404 | BWI | Scat |  | - |  |  | - |  | Yes | Yes | *Gehyra ocellata* |
| 69 | C4432 | BWI | Scat |  | - |  |  | - |  | Yes | Yes | *Gehyra ocellata* |
| 70 | C4415 | BWI | Scat |  | - |  |  | - |  | Yes |  |  |
| 71 | K-34 | Karratha | Scat | 13 | 84.3 | Yes | 11.5 | 84.9 | Yes | Yes | Yes | *Hemidactylus frenatus* |
| 72 | K-31 | Karratha | Scat | 14 | 84.6 | Yes | 12 | 84.9 | Yes | Yes | Yes | *Hemidactylus frenatus* |
| 73 | C4197 | BWI | Scat |  | - |  |  | - |  | Yes |  |  |
| 74 | C4431 | BWI | Scat |  | - |  |  | - |  | Yes |  |  |
| 75 | K-22 | Karratha | Scat | 13.25 | 84.1 | Yes | 14 | 85 | Yes | Yes | Yes | *Hemidactylus frenatus* |
| 76 | C4446 | BWI | Scat |  | - |  |  | - |  | Yes |  |  |
| 77 | K-30 | Karratha | Scat | 13 | 84.3 | Yes | 11.25 | 85 | Yes | Yes | Yes | *Hemidactylus frenatus* |
| 78 | C4416 | BWI | Scat |  | - |  |  | - |  | Yes |  |  |
| 79 | K-35 | Karratha | Scat | 16 | 84.5 | Yes | 16 | 84.9 | Yes | Yes | Yes | *Hemidactylus frenatus* |
| 80 | C4426 | BWI | Scat |  | - |  |  | - |  | Yes | Yes | *Gehyra ocellata* |
| 81 | K-21 | Karratha | Scat | 17.25 | 84.7 | Yes | 19.5 | 84.6 | Yes |  |  | *Hemidactylus frenatus* |
| 82 | C4430 | BWI | Scat |  | - |  |  | - |  | Yes |  |  |
| 83 | C4429 | BWI | Scat |  | - |  |  | - |  | Yes | Yes | *Gehyra ocellata* |
| 84 | C4428 | BWI | Scat |  | - |  |  | - |  | Yes |  |  |
| 85 | C4186 | BWI | Scat |  | - |  |  | - |  | Yes |  |  |
| 86 | C4422 | BWI | Scat |  | - |  |  | - |  | Yes |  |  |
| 87 | C4195 | BWI | Scat |  | - |  |  | - |  | Yes |  |  |
| 88 | K-20 | Karratha | Scat |  | - |  |  | - |  | Yes |  |  |
| 89 | C4420 | BWI | Scat |  | - |  |  | - |  | Yes | Yes | *Cryptoblepharus plagiocephalus* |
| 90 | K-38 | Karratha | Scat | 19 | 84.7 | Yes | 22.5 | 84.6 | Yes |  |  | *Hemidactylus frenatus* |
| 91 | C4417 | BWI | Scat |  | - |  |  | - |  | Yes |  |  |
| 92 | C4427 | BWI | Scat |  | - |  |  | - |  | Yes | Yes | *Gehyra ocellata* |
| 93 | K-32 | Karratha | Scat | 10.8 | 84.4 | Yes | 12.75 | 84.7 | Yes | Yes | Yes | *Hemidactylus frenatus* |
| 94 | C4411 | BWI | Scat |  | - |  |  | - |  | Yes | Yes | *Gehyra ocellata* |
| 95 | C4433 | BWI | Scat |  | - |  |  | - |  | Yes | Yes | *Gehyra ocellata* |
| 96 | C4173 | BWI | Scat |  | - |  |  | - |  | Yes |  |  |
| 97 | C4434 | BWI | Scat |  | - |  |  | - |  | Yes | Yes | *Gehyra ocellata* |
| 98 | C4435 | BWI | Scat |  | - |  |  | - |  | Yes |  |  |
| 99 | C4441 | BWI | Scat |  | - |  |  | - |  | Yes | Yes | *Gehyra variegata* |
| 100 | C4184 | BWI | Scat |  | - |  |  | - |  | Yes | Yes | *Cryptoblepharus plagiocephalus* |
| 101 | K-36 | Karratha | Scat | 12.75 | 85.9 | Yes | 11.25 | 84.9 | Yes | Yes | Yes | *Hemidactylus frenatus* |
| 102 | C4425 | BWI | Scat |  | - |  |  | - |  | Yes | Yes | *Gehyra variegata* |
| 103 | K-41 | Karratha | Scat |  | - | No |  | - |  | Yes | Yes | *Hemidactylus frenatus* |
| 104 | C4405 | BWI | Scat |  | - |  |  | - |  | Yes |  |  |
| 105 | C4425 | BWI | Scat |  | - |  |  | - |  | Yes | Yes | *Gehyra ocellata* |
| 106 | C4436 | BWI | Scat |  | - |  |  | - |  | Yes | Yes | *Gehyra ocellata* |
| 107 | C4421 | BWI | Scat |  | - |  |  | - |  | Yes |  |  |
| 108 | C4450 | BWI | Scat |  | - |  |  | - |  | Yes | Yes | *Gehyra ocellata* |
