# Supplementary material for: A new molecular tool for detection of the highly invasive gecko, Hemidactylus frenatus
Source: PLoS One. 2026 Feb 4;21(2):e0338377. doi: 10.1371/journal.pone.0338377 (PMC12871987; doi:10.1371/journal.pone.0338377)
Supplement: Attachment — Submitted filename: PONE-D-25-13902_R3_review.pdf [file pone.0338377.s009.pdf]

PLOS ONE

A new molecular tool for detection of the highly invasive gecko, Hemidactylus frenatus

--Manuscript Draft--

**Manuscript Number:**	PONE-D-25-13902R3
**Article Type:**	Research Article
**Full Title:**	A new molecular tool for detection of the highly invasive gecko, Hemidactylus frenatus
**Short Title:**	LAMP assay for detection of Hemidactylus frenatus
**Corresponding Author:**	Mark Blacket<br>Agriculture Victoria<br>Bundoora, Victoria AUSTRALIA
**Keywords:**	Gecko; Hemidactylus frenatus; LAMP assay; Invasive species; Trace detection
**Abstract:**	The Common House Gecko Hemidactylus frenatus, also known as the Asian House Gecko (AHG), is the most significant invasive gecko globally. Detecting this species can be challenging because it closely resembles other geckos, and is often not directly observed, being cryptic and nocturnal. Traces such as scats, however, are more readily observed than the animal itself. Here, we developed and tested a new diagnostic mitochondrial ND2 LAMP (Loop-mediated isothermal amplification) assay to detect and distinguish AHG from other geckos. Testing DNA from twenty-five non-target gecko and skink species present in Western Australia demonstrated the species-specificity of the assay. This new molecular assay was found to be highly specific with amplification in under 15 minutes from AHG DNA. Intraspecific variation did not adversely affect the LAMP assay, with all AHG tissue samples successfully amplifying. Including samples from the Cocos (Keeling) Islands territory of Australia which were >6% genetically divergent from mainland Australian samples, representing a genetic group that was previously unknown, referred to here as AHG "clade E". The assay was found to be highly sensitive, capable of amplifying AHG DNA at very low levels, down to 0.0001 ng/µL of AHG DNA, within 25 minutes. The new LAMP assay has been fully optimised for in-field use, including development and testing of a non-destructive DNA extraction method for in-field extractions from both AHG tissue and scat samples, as well as a gBlock gene fragment for use as a synthetic positive control. The in-field protocols were tested on 100 field collected scats, from multiple lizard species in Western Australia, demonstrating the AHG specificity of the assay, with amplification successful on 79% of AHG scats tested in-field. While the assay was highly effective, scat DNA degradation and inhibitors limited detection in 21% of AHG samples, highlighting preservation challenges. This new assay has already been applied operationally in the field, providing early detection of AHG, and preventing potential introduction of this species into new areas.
**Order of Authors:**	Arati Agarwal
	Melissa L Thomas
	Yvette Hitchen
	Paul Doughty
	Simon J. McKirdy
	Brendan C. Rodoni
	Mark James Blacket
**Response to Reviewers:**	Response To Reviewers - PONE-D-25-13902R2<br><br>Reviewer #2: The authors have addressed all the comments raised in the previous version of the manuscript. In the present state, the manuscript is acceptable for the publication.<br>Thank you.<br><br>Reviewer #3: This manuscript describes development of a novel tool for detection of an invasive species. The work is technically sound and worthy of publication. The

Powered by Editorial Manager® and ProduXion Manager® from Aries Systems Corporation

manuscript is generally well written, but there are some corrections and clarifications needed to meet the standard for publication. I have provided comments and suggested changes on the attached PDF and summarise the recommendations here:

General comments
Check usage of commas, these are sometimes missing where appropriate to use and in other cases are included inappropriately. I have made suggested changes on the PDF but please check throughout
DONE

The term 'in-field' is sometimes spelled as "infield" which isn't the intended meaning - make sure "in-field" is used consistently
DONE

There are several sentences with repeated use of the same or very similar terms. While not strictly incorrect, these can be jarring to read, and I'd recommend rewording to avoid repetition where possible. I have commented on examples in the PDF
DONE

The word "as" is used a lot in the text, with various meanings. While its meaning is usually clear from the context, I'd prefer to see "because" used instead of "as" where appropriate. I have highlighted one example in the PDF but there are several. This is just a stylistic preference, but it can help readability and clarity.
DONE

In several places reference is made to "see below" or "as above" or similar. it would be better to refer to the specific relevant section in each case.
DONE

There are some long sentences that are difficult to follow. I have suggested splitting these into multiple shorter sentences
DONE

The LAMP anneal derivative temperature is sometimes referred to as just "anneal derivative" - make consistent (including "temperature" makes most sense)
DONE

I have not reviewed figures because they are not visible within this submission or via the reviewer log in
Noted

Executive summary
Lines 28 and 39 - I would prefer the authors use a term such as demonstrated/demonstrating or supported/supporting rather than confirmed/confirming when referring to the assay specificity. While the level of validation reported here is acceptable, I'd be hesitant to say that specificity is "confirmed" without much wider application of the assay
DONE. Changed as suggested.

Line 29 - 'specific' doesn't appear to make sense here - should this be 'sensitive'?
NOT DONE. For LAMP assays it is common practice to refer to testing an assay against a species-panel as “specificity testing”, whilst testing against known DNA concentrations (e.g. a dilution series) to determine the limits of detection is referred to as “sensitivity testing”. We have used these terms throughout this paper.

Line 40 - Sentence starting "While highly effective," - This reads as though degradation was effective. You could just leave this out, otherwise I'd suggest saying "While the assay was highly effective,"
DONE. Changed as suggested.

Introduction
Line 49 - repetition of "morphological"
DONE. Deleted as suggested.

Powered by Editorial Manager® and ProduXion Manager® from Aries Systems Corporation

Line 115 - suggest inserting "potentially" before "outperforming". Molecular methods weren't formally compared with traditional methods here, so this is speculative.
DONE. Added as suggested.

Line 124-125 - unnecessary repetition of "AHG".
DONE. Replaced 2 x “AHG” with “it”.

Line 136 "see below" - add specific reference
DONE. This note is not referring to other studies, but refers to the present work, where further details of the collection sites for our study are provided. Specific reference to the relevant section has been added (as comment above).

M&M
Line 153 - protocol should be protocols
DONE. Changed as suggested.

Line 156 - "remaining fraction" may be a better term rather than "other half" (given the fraction was 2/3 for some samples)
DONE. Changed as suggested.

Lines 156, 164 - You may need to define what you mean by destructive (vs non-destructive as used below) in this context. In my experience, "destructive" is when the organism is destroyed to provide the DNA sample, so I wouldn't think of scat extractions as being "destructive", but there may be different definitions of the term. I'm also unclear how this destructive sampling (line 156) is different from the non-destructive sampling in the next section (line 164).
DONE. Added definitions, at first mention in text. “Destructive extraction (i.e., involving complete sample homogenisation)”; “Non-destructive extraction (i.e., not involving complete sample homogenisation)”.

Paragraph starting line 178 - Similarly to comment above, I think this needs a bit more context in terms of what you mean by "non-destructive" - e.g. do you mean the samples were (or replicated?) tissue naturally shed from the animal? Saying "in-field" when you were using museum samples also seems inconsistent/confusing. I assume you mean you used museum samples to simulate in-field extraction? Suggest re-wording to clarify.
DONE. As in above comment (i.e., terms are now defined in text). “Non-destructive” extraction applies here as well as the tissue samples were not homogenised.
Line 188 - Sentence starting "Most AHG" is very long - suggest splitting into 2-3 sentences
DONE. Changed as suggested.

Line 196-197 - put as "Australian Genome Research Facility (AGRF)"
DONE. Changed as suggested.

Line 212 - "comprise" may be a better term than "include" here
DONE. Changed as suggested.

Line 223 - the abbreviations used for the primer pairs should be defined. I'd suggest doing this within section 2.4.1 (suggest after sentence finishing at line 217). but it could be done at the start of section 2.4.2 or even in the intro if preferred. Currently this information is presented in the results (section 3.1) but would be better placed in the methods.
DONE. Changed as suggested. Added primer definitions here and removed from section 3.1.

Line 239 - as above, define F2 and B2
DONE. Changed as suggested. Added a reference to “FIP and BIP being comprised of two primer regions, F1+F2 and B1+B2 respectively” to the above definitions.

Line 247 - use "was" instead of "being" to make this sentence grammatically correct
DONE. Changed as suggested.

Line 259-260 - reads as "copy number...was prepared" Suggest you mean something

like "copy number was determined, and..."
DONE. Changed as suggested.

Line 262 - add specific reference
DONE. "… conditions as mentioned above…" refers to the present study, not a previously published study. Specific reference to the relevant section has been added (as comment above).

Line 262-263 - The term "run time" appears to have a different meaning here from that in line 247 leading to some confusion about what is meant. Suggest rewording to clarify.
DONE. Changed as suggested. Have deleted 1 x "run time" in text, and changed another to "amplification time", for clarity. "Run time" is now only used to refer to the complete LAMP assay amplification + annealing time.

Line 272 - remove "for"
DONE. Changed as suggested.

Line 281, 293, 305 - add specific references
DONE. As above, these sentences all provide directions to refer to other sections of the present study, not a previously published study. Specific reference to the relevant section has been added (as comment above).

Line 304-305 - suggest "and" in place of second instance of "using" to avoid repetition
DONE. Changed as suggested.

Results
Line 311-313 - this sentence provides a definition of the primers that is needed (and better placed) in the methods
DONE. Changed as suggested (see above comment).

Line 326 - reference to Table 2 here implies that Table 2 shows barcoding results, but rather it shows the primers that were used - the cross-reference needs clarification.
DONE. Deleted "Table 2", making it clear that Table 3 (and Fig 2) provide the DNA barcoding results. have also added a reference to "S1 Supplementary Table" here, as it also includes DNA barcoding identifications.

Line 367 - Remove "Overall"
DONE. Changed as suggested.

Line 369 - "temperature" rather than "profile"
DONE. Changed as suggested.

Line 371 - suggest "and" in place of second instance of "confirming"
DONE. Changed as suggested.

Line 380 - "at" instead of "as"
DONE. Changed as suggested.

Line 390 - "by LAMP" rather than "through LAMP" is probably better terminology
DONE. Changed as suggested.

Line 405 - first sentence in section 3.5. This sentence needs rewording. Firstly it reads as "the sensitivity ... was quite sensitive" -suggest "quite high" instead. Also ", detecting" isn't grammatically correct here - suggest ", with as low as...copies/uL detected within 15 minutes"
DONE. Changed as suggested.

Line 407 - generally you refer to copies per uL so spelling out the number of copies here is redundant (if included, put as 100,000 not as text)
DONE. Changed as suggested.

Line 410-411 - sentence starting "The amplification time" currently reads as "the amplification time ... amplified in 8 minutes" - needs rewording to be grammatically

correct. Also "starting DNA" is unclear here - do you mean the highest DNA concentration within the dilution series? Neat DNA concentration prior to dilution?
DONE. Changed as suggested.

Line 416-417 - suggest remove second (redundant) use of "LAMP" in this sentence
DONE. Changed as suggested.

Line 417 - Suggest "Across" rather than "In"
DONE. Changed as suggested.

Line 420 - Suggest rewording for better clarity readability, e.g. "... (NTC) did not amplify in any of the 18 runs, "
DONE. Changed as suggested.

Line 423 - I don't think you have enough evidence to "prove" robustness of the assay yet - suggest "demonstrating" as a better term.
DONE. Changed as suggested.

Line 431 - "The" instead of "This"
DONE. Changed as suggested.

Line 432-434 - Sentence starting "DNA dilutions" reads as "DNA dilutions ... were less sensitive" which doesn't make sense. The sentence is also not grammatically correct - needs to be reworded. Suggested rewording is provided on the attached PDF
DONE. Changed as suggested in the PDF.

Discussion
Line 452 - suggest adding "from other groups" at the end of the sentence
DONE. Changed as suggested.

Line 453 - "and" instead of "which"
DONE. Changed as suggested.

Line 456 - suggest "yielded" rather than "possessed" as a better term here
DONE. Changed as suggested.

Line 469-470 - suggest rewording to avoid repetition
DONE. Changed as suggested.

Line 472 - "rate" is probably a better term than "speed" here
DONE. Changed as suggested.

Line 495 - remove "size"
DONE. Changed to “amount” for clarity.

Reviewer #4: This is the first time I reviewed the manuscript although it has already been under one circle of review. Unfortunately, I was not provided with the Figures and I could not provide any comments on their quality.
Noted

I have seen that both reviewers have provided comments regarding the structure of the Introduction. It is obvious that both reviewers have different writing styles, yet they are right. The Introduction and its paragraphs are not nicely connected to each other. It reads as if they were written separately and placed into the introduction. Although I do not have many suggestions on the paragraphs themselves, the nice flow between them is missing. For example, you describe nicely NGS for scats on the 2nd paragraph and all of the sudden you move to LAMP on the 3rd paragraph. What if you mention at the beginning of the 3rd paragraph something along the lines of “for NGS sampling and samples with low quality DNA, we need to use new sensitive methods that are effective and easy to use in the field. This is LAMP”.
DONE. Linking sentences between paragraphs have been added to the introduction, and other parts of the introduction have been rearranged to improve flow between paragraphs. Additionally, the use of gBlocks as positive controls for LAMP has been

	moved to the Methods section, as it did not fit in well with the introduction.<br><br>There are a few issues with the population and phylogeography analyses of this study. The methodology of many of the outputs described in the Results, are not presented in the "materials and methods".<br>DONE. Changed as suggested (see other comments).<br><br>Abstract<br>Lines 26-30: This section is a bit confusing as there are two sentences where the time for the detection of AHG DNA varies. Is the second time (15 mins) an average time whereas the 25 mins for very low DNA concentrations? You should provide clarifications.<br>DONE. Changed as suggested. The terms assay "sensitivity" and "specificity" are used here again (see comment above) to differentiate between detection thresholds, and species-specificity, respectively.<br><br>Introduction<br>Line 52: Replace "e.g." with "e.g.,". Check this issue throughout the manuscript.<br>DONE. Changed as suggested.<br><br>Methods and Methods<br>Line 158: Was the PowerFecal® DNA kit not as successful as the QIAmp® Fast DNA Stool Mini kit? If that is the case, you should describe the results/comparison.<br>DONE. The reference to the preliminary work using the PowerFecal kit have been removed, as this kit was not used past initial testing.<br><br>Line 183: Leave a space between numbers and units. Check this issue throughout the manuscript.<br>DONE. Changed as suggested.<br><br>Line 188: ND2. This is the first time you use the term for the gene. Please provide the full name.<br>DONE. Changed as suggested.<br><br>Lines 188-197: Here you have used new primers, but you have not provided the complete PCR conditions, nor the volumes and concentrations of the used reagents.<br>DONE. Have clarified that the PCR conditions were as per the previously published study, apart from the annealing temperature being reduced to 56oC.<br><br>Lines 199-201: How were the raw sequences processed? There is no initial information about it and the authors go straight to describe how the obtained a ML tree.<br>DONE. Added extra information about how sequences were processed.<br><br>Line 204: What do the authors mean with "worldwide sequences"? Are they referring to sequences of specimens from around the world or the species' distribution? Please clarify.<br>DONE. Changed as suggested.<br><br>Line 286: Section 2.6 is missing.<br>DONE. Corrected labelling of sections.<br><br>Results<br>Lines 311-313: You should rephrase this sentence as it reads as if you have 12 primers.<br>DONE. This information has been modified and moved to the methods (see above comment).<br><br>Lines 325-326: This is a repetition as the details, and the method has been described in the previous section.<br>DONE. This information has been modified (see above comment).<br><br>Lines 326-327: How was the haplotype network created? You have not described the details in the methods.<br>NOT DONE. Detailed information on how the haplotype network was constructed is

	already in the methods.<br><br>Line 331: “highly divergent lineage (>6% different),” how was this number generated?<br>DONE. This information has been added to the Methods.<br><br>Lines 332-334: This part should move to the discussion. This is not a result.<br>DONE. This has been changed as suggested.<br><br>Lines 347-349: Something is missing in this sentence.<br>DONE. This has been modified as suggested.<br><br>Lines 370-372: Please rephrase the sentence as the use of “confirming” is confusion.<br>DONE. This has been modified as suggested (see comment above).<br><br>Lines 405-407: “The detection sensitivity” ………… “was quite sensitive, detecting”.<br>Please rephrase the sentence.<br>DONE. This has been modified as suggested (see comment above).<br><br>Lines 407-408: “one hundred thousand copies (1 x 105 copies/μL)” Keep only one. It is the same thing.<br>DONE. This information has been modified (see above comment).<br><br>Discussion<br>Line 450: Replace “Table” with “Tables”.<br>DONE. This has been changed as suggested.<br><br>Line 460: “36% different for the section” how did the authors come with this number?<br>DONE. This information has been added to the Methods (see above comment).<br><br>I have also seen that some of the previous reviewers’ comments have not been addressed. For example, the research permit for the samples from Indonesia. The ethics permits and “Cadaver and Tissue Usage” of one country is not the same when that involves samples collected at a different country. An ethics permit is not a research permit.<br>DONE. The preserved samples obtained from overseas were sourced directly from the Western Australian Museum, as stated in the Acknowledgments. The wording has been modified to make it clearer that samples were provided through the WAM, or an animal ethics cadaver and/or tissue notification form.<br><br>Additionally, the suggestion of a previous reviewer about “A comparative discussion with other available molecular tools (e.g., conventional PCR, qPCR, or other isothermal methods) would further highlight the advantages and limitations of the proposed technique, providing readers with a clearer perspective on its applicability.” has not been addressed. The reply that the authors provide in the text, does not address the suggestion. The discussion would actually benefit from it.<br>DONE. A discussion of other molecular approaches, including real-time PCR, compared with LAMP, for molecular diagnostics has been added to the Discussion.
**Additional Information:**	
**Question**	**Response**
**Financial Disclosure**<br><br>Enter a financial disclosure statement that describes the sources of funding for the work included in this submission. Review the submission guidelines for detailed requirements. View published research articles from *PLOS ONE* for specific examples.	Yes

This statement is required for submission and **will appear in the published article** if the submission is accepted. Please make sure it is accurate.<br><br>**Funded studies**<br>Enter a statement with the following details:<br>• Initials of the authors who received each award<br>• Grant numbers awarded to each author<br>• The full name of each funder<br>• URL of each funder website<br>• Did the sponsors or funders play any role in the study design, data collection and analysis, decision to publish, or preparation of the manuscript?<br><br>Did you receive funding for this work?	
Please add funding details.<br>as follow-up to "**Financial Disclosure**<br><br>Enter a financial disclosure statement that describes the sources of funding for the work included in this submission. Review the submission guidelines for detailed requirements. View published research articles from *PLOS ONE* for specific examples.<br><br>This statement is required for submission and **will appear in the published article** if the submission is accepted. Please make sure it is accurate.<br><br>**Funded studies**<br>Enter a statement with the following details:<br>• Initials of the authors who received each award<br>• Grant numbers awarded to each author<br>• The full name of each funder<br>• URL of each funder website<br>• Did the sponsors or funders play any role in the study design, data collection and analysis, decision to publish, or preparation of the manuscript?<br><br>Did you receive funding for this work?"	This study was supported through Murdoch University grant MU-112020, and by the State Government of Victoria (Agriculture Victoria Research). This study was funded by the Gorgon Project. The Gorgon Project is operated by an Australian subsidiary of Chevron and is a joint venture of the Australian subsidiaries of Chevron (47.3%), ExxonMobil (25%), Shell (25%), Osaka Gas (1.25%), MidOcean Energy (1%), and JERA (0.417%). The funders had no role in study design, data collection and analysis, decision to publish, or preparation of the manuscript.

Please select the country of your main research funder (please select carefully as in some cases this is used in fee calculation).<br>as follow-up to "**Financial Disclosure**<br><br>Enter a financial disclosure statement that describes the sources of funding for the work included in this submission. Review the submission guidelines for detailed requirements. View published research articles from *PLOS ONE* for specific examples.<br><br>This statement is required for submission and **will appear in the published article** if the submission is accepted. Please make sure it is accurate.<br><br>**Funded studies**<br>Enter a statement with the following details:<br>• Initials of the authors who received each award<br>• Grant numbers awarded to each author<br>• The full name of each funder<br>• URL of each funder website<br>• Did the sponsors or funders play any role in the study design, data collection and analysis, decision to publish, or preparation of the manuscript?<br><br>Did you receive funding for this work?"	AUSTRALIA - AU
**Competing Interests**<br><br>Use the instructions below to enter a competing interest statement for this submission. On behalf of all authors, disclose any competing interests that could be perceived to bias this work—acknowledging all financial support and any other relevant financial or non-financial competing interests.<br><br>This statement is required for submission and **will appear in the published article** if the submission is accepted. Please make sure it is accurate and that any funding	The authors have declared that no competing interests exist.

Powered by Editorial Manager® and ProduXion Manager® from Aries Systems Corporation

sources listed in your Funding Information later in the submission form are also declared in your Financial Disclosure statement.<br><br>View published research articles from *PLOS ONE* for specific examples.<br><br>**NO authors have competing interests**<br><br>Enter: *The authors have declared that no competing interests exist.*<br><br>**Authors with competing interests**<br><br>Enter competing interest details beginning with this statement:<br><br>*I have read the journal's policy and the authors of this manuscript have the following competing interests: [insert competing interests here]*<br><br>* typeset	
**Ethics Statement**<br><br>Enter an ethics statement for this submission. This statement is required if the study involved:<br><br>• Human participants<br>• Human specimens or tissue<br>• Vertebrate animals or cephalopods<br>• Vertebrate embryos or tissues<br>• Field research<br><br>Write "N/A" if the submission does not require an ethics statement.<br><br>General guidance is provided below. Consult the submission guidelines for detailed instructions. **Make sure that all information entered here is included in the Methods section of the manuscript.**	Scat samples from Barrow Island were sourced by Murdoch University under animal ethics permit number R2859/16. Scats samples from Dampier were provided by Chevron Australia. Tissue samples were provided by both the Western Australian Museum and Helix Molecular Solutions. These tissue samples were obtained from preserved specimens from the WAM, or through a Cadaver and Tissue Usage Notification form.

**Format for specific study types**<br><br>**Human Subject Research (involving human participants and/or tissue)**<br>• Give the name of the institutional review board or ethics committee that approved the study<br>• Include the approval number and/or a statement indicating approval of this research<br>• Indicate the form of consent obtained (written/oral) or the reason that consent was not obtained (e.g. the data were analyzed anonymously)<br><br>**Animal Research (involving vertebrate animals, embryos or tissues)**<br>• Provide the name of the Institutional Animal Care and Use Committee (IACUC) or other relevant ethics board that reviewed the study protocol, and indicate whether they approved this research or granted a formal waiver of ethical approval<br>• Include an approval number if one was obtained<br>• If the study involved *non-human primates*, add *additional details* about animal welfare and steps taken to ameliorate suffering<br>• If anesthesia, euthanasia, or any kind of animal sacrifice is part of the study, include briefly which substances and/or methods were applied<br><br>**Field Research**<br><br>Include the following details if this study involves the collection of plant, animal, or other materials from a natural setting:<br>• Field permit number<br>• Name of the institution or relevant body that granted permission	
**Data Availability**<br><br>Authors are required to make all data underlying the findings described fully available, without restriction, and from the time of publication. PLOS allows rare exceptions to address legal and ethical concerns. See the PLOS Data Policy and FAQ for detailed information.	Yes - all data are fully available without restriction

A Data Availability Statement describing where the data can be found is required at submission. Your answers to this question constitute the Data Availability Statement and **will be published in the article**, if accepted.<br><br>**Important:** Stating 'data available on request from the author' is not sufficient. If your data are only available upon request, select 'No' for the first question and explain your exceptional situation in the text box.<br><br>Do the authors confirm that all data underlying the findings described in their manuscript are fully available without restriction?	
**Describe where the data may be found in full sentences. If you are copying our sample text, replace any instances of XXX with the appropriate details.**<br><br>• If the data are **held or will be held in a public repository**, include URLs, accession numbers or DOIs. If this information will only be available after acceptance, indicate this by ticking the box below. For example: *All XXX files are available from the XXX database (accession number(s) XXX, XXX.*).<br>• If the data are all contained **within the manuscript and/or Supporting Information files**, enter the following: *All relevant data are within the manuscript and its Supporting Information files.*<br>• If neither of these applies but you are able to provide **details of access elsewhere**, with or without limitations, please do so. For example:<br><br>*Data cannot be shared publicly because of [XXX]. Data are available from the XXX Institutional Data Access / Ethics Committee (contact via XXX) for researchers who meet the criteria for access to confidential data.*<br><br>*The data underlying the results presented in the study are available from (include the name of the third party*	All relevant data are within the paper and supporting information files (S1 & S2 files). ND2 DNA sequences from this study have been accessioned in GenBank (PQ390829 - PQ390908).

and contact information or URL).<br>• This text is appropriate if the data are owned by a third party and authors do not have permission to share the data.<br><br>* typeset	
Additional data availability information:	

Manuscript

Click here to access/download;Manuscript;Manuscript_R3.docx

A new molecular tool for detection of the highly invasive gecko, *Hemidactylus frenatus*

Arati Agarwal [1], Melissa L Thomas [2], Yvette Hitchen [3], Paul Doughty [4], Simon J. McKirdy [2], Brendan C. Rodoni [1,5], Mark J. Blacket [1*]

[1] Agriculture Victoria Research, AgriBio - Centre for AgriBioscience, 5 Ring Road, Bundoora, VIC 3083, Australia.

[2] Harry Butler Institute, Murdoch University, Perth, WA 6150, Australia

[3] Helix Molecular Solutions, Leederville, WA 6153, Australia

[4] Western Australia Museum, Terrestrial Zoology, Welshpool, WA 6986, Australia

[5] School of Applied Systems Biology, La Trobe University, Bundoora 3083, Victoria, Australia.

**** Corresponding Author:*** Mark Blacket; email: mark.blacket@agriculture.vic.gov.au

Short title: LAMP assay for detection of *Hemidactylus frenatus*

ABSTRACT

The Common House Gecko, *Hemidactylus frenatus*, also known as the Asian House Gecko (AHG), is the most significant invasive gecko globally. Detecting this species can be challenging because it closely resembles other geckos, and is often not directly observed, being cryptic and nocturnal. Traces such as scats, however, are more readily observed than the animal itself. Here, we developed and tested a new diagnostic mitochondrial ND2 LAMP (Loop-mediated isothermal amplification) assay to detect and distinguish AHG from other geckos. Testing DNA from twenty-five non-target gecko and skink species present in Western Australia demonstrated the species-specificity of the assay. This new molecular assay was found to be highly specific with amplification in under 15 minutes from AHG DNA. Intraspecific variation did not adversely affect the LAMP assay, with all AHG tissue samples successfully amplifying. ~~Including~~ samples from the Cocos (Keeling) Islands territory of Australia which were >6% genetically divergent from mainland Australian samples, representing a genetic group that was previously unknown, referred to here as AHG “clade E”. The assay was found to be highly sensitive, capable of amplifying AHG DNA at very low levels, down to 0.0001 ng/µL of AHG DNA, within 25 minutes. The new LAMP assay has been fully optimised for in-field use, including development and testing of a non-destructive DNA extraction method for in-field extractions from both AHG tissue and scat samples, as well as a gBlock gene fragment for use as a synthetic positive control. The in-field protocols were tested on 100 field collected scats, from multiple lizard species in Western Australia, demonstrating the AHG specificity of the assay, with amplification successful on 79% of AHG scats tested in-field. While the assay was highly effective, scat DNA degradation and inhibitors limited detection in 21% of AHG samples, highlighting preservation challenges. This new assay has already been applied operationally in the field,

providing early detection of AHG, and preventing potential introduction of this species into new areas.

1. Introduction

Non-invasive genetic sampling (NGS) has been widely applied over the last twenty years by wildlife biologists [1]. NGS is when DNA is extracted from animal traces, such as from hair [2], shed skin [3], faecal scats [2, 4], or other sources, without handling, capturing or even observing the individual animal. NGS alleviates many of the difficulties associated with conventional identifications including morphological complexities, cryptic life stages, and globally declining taxonomic expertise. Applications of NGS include detection of rare or endangered species [5], conservation management [6], differentiating between morphologically similar species [1], population estimation (e.g., [7]), and population sex-ratios [8]. NGS has also been applied for monitoring of invasive species [4], however, studies using NGS as an early detection tool for biosecurity purposes remain rare.

One of the primary issues facing NGS of animals, is the quality of trace DNA. Animal faecal scats are one of the most common NGS sources, however scats contain very little host animal DNA and considerable concentrations of inhibitors [1]. The diet of the animal can play a significant role in the success of DNA extraction from scats. For example, insect material in scats is known to have an inhibitory effect on amplification success when DNA concentrations of the target species are low [9]. Furthermore, scat samples are generally collected from wild animals whose scats are likely to have been exposed to a variety of weather conditions which can degrade the DNA prior to the scat being collected. Other factors, such as DNA extraction method and sample preservation can also influence the success of extracting species DNA from scats [1]. These challenges highlight the need for more sensitive molecular techniques capable of detecting low-quality DNA under field conditions.

Loop-mediated isothermal amplification (LAMP) offers a promising alternative to conventional PCR based approaches. LAMP enables rapid amplification of target DNA with high specificity, sensitivity, and robustness under isothermal conditions [10]. Assays can be conducted under field conditions using portable equipment and provide robust results in less than one hour. Importantly, LAMP is relatively tolerant to common inhibitors, is highly efficient, and can sensitively detect low concentrations of target DNA (e.g., [11-14]), making it particularly suitable for use on NGS sources of DNA.

The Common House Gecko (*Hemidactylus frenatus* Dumeril & Bibron), also known as the Asian House Gecko (AHG), is one of the world’s most successful invasive geckos [15, 16]. In parts of its introduced range, *H. frenatus* has invaded natural habitats where impacts on native geckos have been demonstrated [17], or inferred [18, 19]. This species poses an ongoing biosecurity risk; in New Zealand it accounts for up to 44% of all intercepted herpetofauna [20], and on a remote Australian island, it is the most frequently detected gecko intercepted at the border [21]. It presents particular risks to island ecosystems, which are often the last refuges for endangered species [22]. Detecting incursions of this species rapidly is important as populations can quickly achieve high densities that may preclude eradication [18].

Currently, *H. frenatus* detection relies primarily on auditory and visual surveys. Males produce distinctive calls [16], but these are limited to specific behavioural contexts and environmental conditions, with typically only adult males calling in the presence of females, and only during warmer months around sunset and sunrise [16]. Visual surveys also require

expertise to distinguish *H. frenatus* from morphologically similar native geckos (e.g., *Gehyra variagata* in Australia). Similarly, scats from different gecko species can appear almost identical. For these reasons, scat collections, combined with conventional PCR followed by DNA sequencing, are often used to confirm species presence [21]. However, such molecular methods are constrained by relatively long turnaround times (typically 2 to 4 weeks from sample collection to analysis), which limits their use as an early detection tool.

Development of a targeted LAMP assay for *H. frenatus* could provide a rapid, specific and field-deployable molecular tool capable of detecting AHG DNA from scats within one hour. Although LAMP is generally less sensitive than real-time PCR, its speed, simplicity, and inhibitor tolerance make it an attractive option for biosecurity surveillance. LAMP assays have already been developed successfully to aid early detection of several invasive invertebrate species, including fruit flies *Bactrocera tryoni* [23], *B. trivialis* [24], fall armyworm *Spodoptera frugiperda* [14], grape phylloxera *Daktulosphaira vitifoliae* [11], and honeybee ectoparasitic *Varroa* mites [13]. NGS LAMP assays have also been successfully developed and employed to detect traces of two invasive species, including one vertebrate species; shed larval skins of the Khapra beetle *Trogoderma granarium* [12], and traces of DNA of the red fox *Vulpes vulpes* that scavenged on harbour porpoise carcasses [25].

In this study, we developed and validated a novel LAMP assay for the detection of *H. frenatus* (AHG). We used NGS (from small reptile scats) and tissue samples to evaluate DNA assay specificity and sensitivity. Field testing was conducted on scats collected from (i) mainland Australia where *H. frenatus* is established, and (ii) Barrow Island, where the species is currently absent, but represents an ongoing incursion threat.

2. MATERIALS AND METHODS

2.1 Specimens Examined

For developing, optimising, and testing the specificity of our LAMP assay, we used 26 tissue samples from the target species (AHG) together with 54 tissue samples from twenty-five Australian native gecko and skink species which co-occur geographically with AHG in Western Australia (Table 1). Samples were sourced by Chevron Australia, Helix Molecular Solutions (Helix), and the Western Australian Museum (WAM). One hundred and twenty lizard scat samples were used for optimising (n=20) and field validating (n=100) the assay, collected from multiple sites in Western Australia (see section 2.7 and S1 Supplementary Table).

Table 1. Gecko and skink DNA samples (from tissue) used for development and initial testing of AHG LAMP assay. AHG samples are indicated by grey shading. "N/A" indicates no amplification produced with the new AHG LAMP assay, QE = QuickExtract, Qiagen = DNeasy extraction, Helix = Helix Molecular Solutions, WAM = Western Australian Museum, ND2 = mitochondrial ND2 locus.

	**Species**	**Sample**	**Source**	**Extraction method**	**LAMP Time (min)**	**LAMP Anneal (°C)**	**GenBank Accession (ND2)**
1	*Hemidactlyus frenatus*	BS01 Karratha, Australia	Helix	Qiagen	9.25	85.5	PQ390829
2	*Hemidactlyus frenatus*	BS10 Karratha, Australia	Helix	Qiagen	9.00	85.5	PQ390830
3	*Hemidactlyus frenatus*	BS26 Batam, Indonesia	Helix	Qiagen	12.25	85.5	PQ390831
4	*Hemidactlyus frenatus*	BS28 Batam, Indonesia	Helix	Qiagen	15.00	86.2	PQ390832
5	*Hemidactlyus frenatus*	HR18 Intercepted	Helix	Qiagen	8.50	85.3	PQ390833
6	*Hemidactlyus frenatus*	HR19 Intercepted	Helix	Qiagen	8.50	85.2	PQ390834
7	*Hemidactlyus frenatus*	R131793, Kununurra, Australia	WAM	QE	7.25	85.3	PQ390835
8	*Hemidactlyus frenatus*	R131879, Kununurra, Australia	WAM	QE	7.50	85.2	PQ390836
9	*Hemidactlyus frenatus*	R132949, Broome, Australia	WAM	QE	9.75	85.3	PQ390837
10	*Hemidactlyus frenatus*	R132952, Broome, Australia	WAM	QE	10.00	85.3	PQ390838
11	*Hemidactlyus frenatus*	R164914, Truscott Airstrip, Australia	WAM	QE	13.25	85.4	PQ390839
12	*Hemidactlyus frenatus*	R172512, Intercepted	WAM	QE	16.25	85.8	PQ390840
13	*Hemidactlyus frenatus*	R172517, Intercepted	WAM	QE	9.25	85.3	PQ390841
14	*Hemidactlyus frenatus*	R172557, Intercepted	WAM	QE	11.25	85.3	PQ390842
15	*Hemidactlyus frenatus*	R174322, Port Hedland, Australia	WAM	QE	7.50	85.1	PQ390843
16	*Hemidactlyus frenatus*	R174747, Carnarvon, Australia	WAM	QE	7.50	85.3	PQ390844
17	*Hemidactlyus frenatus*	R173044, Cocos (Keeling) Island	WAM	QE	10.75	85.1	PQ390845
18	*Hemidactlyus frenatus*	R173054, Cocos (Keeling) Island	WAM	QE	13.25	85.1	PQ390846
19	*Hemidactlyus frenatus*	R112253, Yamdena Island, Indonesia	WAM	QE	11.75	85.6	PQ390847

20	*Hemidactlyus frenatus*	R109929, Yamdena Island, Indonesia	WAM	QE	13.25	85.6	PQ390848
21	*Hemidactlyus frenatus*	R109377, Banda island, Indonesia	WAM	QE	11.50	85.2	PQ390849
22	*Hemidactlyus frenatus*	R109370, Banda island, Indonesia	WAM	QE	6.50	85.4	PQ390850
23	*Hemidactlyus frenatus*	R109230, Nusa Penida Island, Indonesia	WAM	Qiagen	8.00	85.4	PQ390851
24	*Hemidactlyus frenatus*	R109517, Wokam island, Indonesia	WAM	QE	7.00	85.4	PQ390852
25	*Hemidactlyus frenatus*	R109520, Wokam island, Indonesia	WAM	Qiagen	8.25	85.5	PQ390853
26	*Hemidactlyus frenatus*	R109879, Ambon Island, Indonesia	WAM	QE	6.50	85.4	PQ390854
27	*Gehyra pilbara*	EA01 R165746, Australia	Helix	Qiagen	N/A	N/A	PQ390855
28	*Gehyra pilbara*	EA02 R165753, Australia	Helix	Qiagen	N/A	N/A	PQ390856
29	*Gehyra variegata*	EA03 R154142, Australia	Helix	Qiagen	N/A	N/A	PQ390857
30	*Gehyra variegata*	EA04 R165763, Australia	Helix	Qiagen	N/A	N/A	PQ390858
31	*Heteronotia binoei*	EA07 R154100, Australia	Helix	Qiagen	N/A	N/A	PQ390859
32	*Heteronotia binoei*	EA08 R154114, Australia	Helix	Qiagen	N/A	N/A	PQ390860
33	*Lucasium stenodactylum*	EA09 T20121209.HPZ2-03lucste, Australia	Helix	Qiagen	N/A	N/A	PQ390861
34	*Strophurus jeanae*	EA11 R154145, Australia	Helix	Qiagen	N/A	N/A	PQ390862
35	*Carlia triacantha*	EA12 T20130323.22HFZ2-03CARTRI.01, Australia	Helix	Qiagen	N/A	N/A	PQ390863
36	*Carlia triacantha*	EA13 T20130412.23HPZ2-02CARTRI.01, Australia	Helix	Qiagen	N/A	N/A	PQ390864
37	*Cryptoblepharus plagiocephalus*	EA14 R154110, Australia	Helix	Qiagen	N/A	N/A	PQ390865
38	*Cryptoblepharus plagiocephalus*	EA15 R154113, Australia	Helix	Qiagen	N/A	N/A	PQ390866
39	*Ctenotus grandis*	EA16 R154081, Australia	Helix	Qiagen	N/A	N/A	PQ390867
40	*Ctenotus grandis*	EA17 R154084, Australia	Helix	Qiagen	N/A	N/A	PQ390868
41	*Ctenotus Pantherinus acripes*	EA18 R154151, Australia	Helix	Qiagen	N/A	N/A	PQ390869
42	*Ctenotus Pantherinus acripes*	EA19 R154168, Australia	Helix	Qiagen	N/A	N/A	PQ390870
43	*Ctenotus saxatilis*	EA20 R154092, Australia	Helix	Qiagen	N/A	N/A	PQ390871
44	*Ctenotus saxatilis*	EA21 R154099, Australia	Helix	Qiagen	N/A	N/A	PQ390872
45	*Ctenotus serventyi*	EA22 T20120521.HPZ2A-06.01, Australia	Helix	Qiagen	N/A	N/A	PQ390873
46	*Cyclodomophus melanops*	EA23 R154130, Australia	Helix	Qiagen	N/A	N/A	PQ390874
47	*Cyclodomophus melanops*	EA24 R154160, Australia	Helix	Qiagen	N/A	N/A	PQ390875
48	*Eremiascincus richardsonii*	EA25 R165786, Australia	Helix	Qiagen	N/A	N/A	PQ390876
49	*Eremiascincus richardsonii*	EA26 T20121113.OFZ1-02ERERIC.01, Australia	Helix	Qiagen	N/A	N/A	PQ390877
50	*Eremiascincus isolepis*	EA27 T20120519.HFZ1-03.01, Australia	Helix	Qiagen	N/A	N/A	PQ390878

51	*Eremiascincus isolepis*	EA28 T20120520.HFZ2-02.01, Australia	Helix	Qiagen	N/A	N/A	PQ390879
52	*Lerista bipes*	EA29 R154118, Australia	Helix	Qiagen	N/A	N/A	PQ390880
53	*Lerista bipes*	EA30 R154121, Australia	Helix	Qiagen	N/A	N/A	PQ390881
54	*Lerista clara*	EA31 R154140, Australia	Helix	Qiagen	N/A	N/A	PQ390882
55	*Lerista clara*	EA32 R154166, Australia	Helix	Qiagen	N/A	N/A	PQ390883
56	*Metetia greyii*	EA33 R154144, Australia	Helix	Qiagen	N/A	N/A	PQ390884
57	*Metetia greyii*	EA34 R154173, Australia	Helix	Qiagen	N/A	N/A	PQ390885
58	*Morethia ruficauda*	EA35 R154174, Australia	Helix	Qiagen	N/A	N/A	PQ390886
59	*Notoscincus ornatus*	EA37 R154137, Australia	Helix	Qiagen	N/A	N/A	PQ390887
60	*Notoscincus ornatus*	EA38 R154147, Australia	Helix	Qiagen	N/A	N/A	PQ390888
61	*Proablepharus reginae*	EA39 R154135, Australia	Helix	Qiagen	N/A	N/A	PQ390889
62	*Proablepharus reginae*	EA40 R154136, Australia	Helix	Qiagen	N/A	N/A	PQ390890
63	*Ctenotus duricola*	EA41 T20131114.28HPZ2-01.CTEDUR.01, Australia	Helix	Qiagen	N/A	N/A	PQ390891
64	*Ctenotus duricola*	EA42 T20131106.28HPZ2-04.CTEDUR.01, Australia	Helix	Qiagen	N/A	N/A	PQ390892
65	*Lucasium stenodactylum*	EA43 T20131115.28GPZ1-08.LUCSTE.01, Australia	Helix	Qiagen	N/A	N/A	PQ390893
66	*Morethia ruficauda*	EA44 T20131110.28HPZ2-01.MORRUF.01, Australia	Helix	Qiagen	N/A	N/A	PQ390894
67	*Lerista elegans*	EA45 R165983, Australia	Helix	Qiagen	N/A	N/A	PQ390895
68	*Lerista elegans*	EA46 R165978, Australia	Helix	Qiagen	N/A	N/A	PQ390896
69	*Morethia lineoocellata*	EA47 R165977, Australia	Helix	Qiagen	N/A	N/A	PQ390897
70	*Morethia lineoocellata*	EA48 R123950, Australia	Helix	Qiagen	N/A	N/A	PQ390898
71	*Ctenotus hanloni*	EA49 R157543, Australia	Helix	Qiagen	N/A	N/A	PQ390899
72	*Ctenotus hanloni*	EA50 R157557, Australia	Helix	Qiagen	N/A	N/A	PQ390900
73	*Ctenotus serventyi*	EA51 R102812, Australia	Helix	Qiagen	N/A	N/A	PQ390901
74	*Gehyra* sp.	EA55 R165742, Australia	Helix	Qiagen	N/A	N/A	PQ390902
75	*Gehyra* sp.	EA58 R163105, Australia	Helix	Qiagen	N/A	N/A	PQ390903
76	*Gehyra* sp.	EA59 R163044, Australia	Helix	Qiagen	N/A	N/A	PQ390904
77	*Gehyra* sp.	EA60 R165748, Australia	Helix	Qiagen	N/A	N/A	PQ390905
78	*Gehyra* sp.	EA61 R165749, Australia	Helix	Qiagen	N/A	N/A	PQ390906
79	*Gehyra* sp.	EA63 R165255, Australia	Helix	Qiagen	N/A	N/A	PQ390907
80	*Strophurus jeanae*	FD01 T20131203.29GPZ1-06.STRJEA.01, Australia	Helix	Qiagen	N/A	N/A	PQ390908

2.2 Optimisation of DNA Extraction Methods

2.2.1 Laboratory DNA extractions

A destructive DNA extraction method (i.e., involving complete sample homogenisation) was used on AHG samples to obtain "high-quality" DNA with a high yield, using multiple Qiagen (Germany) DNA extraction kits. DNA was extracted from AHG tissue samples (R109230 and R109520, Table 1) using the DNeasy Blood and Tissue extraction kit (Qiagen) to obtain high yield DNA for downstream application to conduct the AHG LAMP sensitivity test (see Results).

Laboratory DNA extractions from scat samples were optimised using a QIAmp® Fast DNA Stool Mini kit following the manufacturers' protocols. Depending on the size of each scat a half or one-third of the scat was used in the DNA extraction with the remaining fraction stored for later use at -20 ^{o}C. A panel of DNA was destructively extracted from twenty AHG scat samples (collected from Dampier, Western Australia), using the extraction kit, which was found to produce DNA with a reasonably high yield. Two of these AHG scat samples were processed for downstream application to conduct the AHG LAMP sensitivity testing.

2.2.2 In-field compatible DNA extractions

Optimising an in-field compatible DNA extraction procedure is a pre-requisite for conducting the LAMP assay in the field. Non-destructive in-field DNA (i.e., not involving complete sample homogenisation) was extracted from three scat samples (collected from Dampier, WA) using commercial QuickExtract^{TM} DNA extraction (QE) solution 1.0 (Epicentre, USA). This method is rapid and easy to use, providing good quality DNA for use as a template in

the LAMP assay. One hundred microliters of QE solution were pipetted into 6-wells of each Genie strip (OptiGene, UK). Depending on the size of the scat only one-third or half of it was transferred into each individual well and broken using a toothpick (single use to prevent cross contamination) [11], with up to six samples processed simultaneously in the Genie III (OptiGene, UK). The in-field QE DNA extraction protocol used the Genie III as an incubator programmed at 65 ^{o}C for 6 min followed by 98 ^{o}C for 2 min (total = 8 min), following published protocols [11]. To experimentally test the effect of reducing possible inhibitors, two microliters of undiluted QE DNA extracted from scats (1:1) was diluted, 1:10, 1:20 and 1:50, in water for use as a template in the LAMP assay.

Additional, non-destructive in-field DNA samples were extracted from 18 AHG tissue (WAM) samples using fifty microliters of QE solution (Table 1). Tissue samples were removed from ethanol and air dried on paper towel for approximately one minute before being transferred into each well of the Genie strip using a toothpick (single use, to prevent cross contamination). Each sample was immersed in QE, with up to six samples processed simultaneously in the Genie III, as above. All DNA samples were stored at -20 ^{o}C for future use.

2.3 Assessment of mitochondrial ND2 DNA sequence variation

Lizard species identifications were confirmed through DNA barcoding of the mitochondrial NADH dehydrogenase subunit 2 (ND2) locus, following published PCR conditions [26], with the PCR annealing temperature reduced to 56 ^{o}C. Most AHG ND2 sequences generated in the current study were obtained using the new AHG LAMP primer “AHG_F3” (Table 2) in combination with the existing primer M1123R, 5’- GCTTAATTAAAGTGTYTGAGTTGC-

3' [26] (~450 bp amplicon), for *Hemidactylus frenatus*. ND2 was amplified from other lizard species using a new 'general' primer "GeckoND2-F" designed to be applicable to this suite of small lizard species 5'- MCAAACMCGAAAAATYATRGC-3', in combination with M1123R (~570 bp amplicon). PCR's consisted of 40 cycles with an annealing temperature of 53 ^{o}C. PCR amplicons were sequenced commercially by Macrogen Inc. (Macrogen, Seoul, Korea) or Australian Genome Research Facility (AGRF) in dual directions.

DNA sequence electropherograms were analysed using the software MEGA7 [27]. Forward and reverse sequences were paired, and a consensus sequence was generated. Genetic similarity of DNA sequences of AHG and non-target lizard species was examined using a maximum likelihood tree constructed in Mega7 [27]. Pairwise sequence differences (p-distances) between species were generated in Mega7 [27] to assess percentage sequence divergences between species. Relationships among AHG samples were also examined through a haplotype network. DNA sequence variation in AHG specimens was examined through generation of a median joining haplotype network ($\varepsilon = 0$) [28], constructed in PopArt [29], using all new AHG sequences obtained here (Table 1, S1 Supplementary Table) and from sequences of specimens from around the world obtained from GenBank [30].

2.4 Development and evaluation of AHG LAMP assay

2.4.1 AHG dataset and primer design

AHG LAMP primers were developed for a section of the mitochondrial ND2 locus, that has been commonly used for DNA barcoding identification of geckos [26, 30]. This locus has been well characterised from multiple *Hemidactylus* species, including AHG which is known

to comprise multiple genetically distinct ND2 clades [30]. Fifty-four ND2 DNA sequences for non-target gecko and skink species that co-occur in Western Australia (samples 27 to 80, Table 1) were generated (as in section 2.3). These were combined with thirty-one DNA sequences of AHG (same region of ND2) downloaded from GenBank, mostly derived from [30]. The combined ND2 alignment was used to design eight novel LAMP primer regions for six primers to target AHG (Table 2), including two inner primers (FIP and BIP, each comprised of two primer regions, F1+F2 and B1+B2 respectively), two outer primers (F3 and B3), and two loop primers Floop and Bloop. For all primers the GC content (%), predicted melting temperature (Tm), and potential secondary structure (hairpins or dimers) were analysed using the Integrated DNA Technologies (IDT) online OligoAnalyzer Tool [31] using the qPCR parameter sets.

2.4.2 LAMP primer ratio optimisation

The primer ratio (F3/B3: FIP/BIP: Floop/Bloop) for this assay was optimised following published protocols using multiple ratios of primers [23]. Optimised primer master mix was prepared by adding the specified amount of each of the six primers in a 1:6:3 ratio. A 100 µL volume of primer master mix 1:6:3 (F3/B3: FIP/BIP: Floop/Bloop) was prepared by adding 10 µL (10 µM) each of F3/B3, 6 µL (100 µM) each of FIP/BIP, 3 µL (100 µM) each of Floop/Bloop and 62 µL of Ultrapure water (Invitrogen, Australia).

2.4.3 LAMP assay conditions

The AHG LAMP assay was performed following published protocols [14]. Each LAMP reaction was conducted in a total volume of 25 µL containing 14 µL of Isothermal Master Mix (Iso-001, OptiGene, UK), 10 µL of the primer master mix (as in section 2.4.2) and 1 µL

of template DNA. A one microliter disposable plastic loop was used to add in-field DNA extract, ensuring that a fresh loop was used for each sample. The use of disposable plastic loops has been shown to be an effective method for transferring template DNA in LAMP reactions [23]. These loops reduce the risk of cross contamination and eliminate the need for pipettes when LAMP is used outside a laboratory, a necessity for effective field deployment.

Table 2. AHG LAMP primer and amplicon sequences (gBlock) and parameters. The F2 and B2 primer regions of FIP and BIP are bold and underlined. Lowercase letters in the gBlock indicate extra "ccc" added between LAMP primer sites to increase the overall Tm of the amplicon.

**Primers**	**Sequence 5'-3'**	**Primer length (bp)**	**Predicted Tm, annealing temperature (°C)**	**Degeneracy of primer (fold)**
AHG_F3	CRCCACAGCCATATTCAYA	19	55.9	4
AHG_B3	GGTAAGCTTGCGGCRAG	17	59.0	2
AHG_FIP	CCAAGTGACATAAGGGTAAYTATTA**CACCACAACCAARACCATTC**	45	76.4	4
AHG_BIP	CTAACAGGRTTYATGCCAAAAT**CGCYGATKGGYAGGAG**	38	73.5	32
AHG_Floop	GGGTTAGGGCTGGTGATAKTA	21	66.9	2
AHG_Bloop	KACTTATCATTAAAGACCTYGC	22	46.1	4
gBlock Fragment	cccCACCACAGCCATATTCACACACCACAACCAAGACCATTCcccTACT ATCACCAGCCCTAACCCcccTAATAATTACCCTTATGTCACTTGGcccCT AACAGGGTTTATGCCAAAATcccGACTTATCATTAAAGACCTTGCcccC TCCTACCAATCGGCGcccCTCGCCGCAAGCTTACCccc	186	N/A	N/A

High-quality DNA (Qiagen) extracted from AHG tissue samples was used as a positive control for optimising the LAMP assay. A no template control (NTC) was included in each test to detect reagent contamination. All the LAMP assays were run in the Genie III at 65 °C for 25 min followed by an annealing curve analysis from 98 °C to 73 °C with ramping at 0.05 °C/s. The total run time was approximately 35 minutes. The amplification and anneal derivative temperature profiles (anneal derivative) were visualised on the Genie III screen to ensure that amplification occurred as expected. Positive results were further confirmed through the annealing step resulting in a single product peak at a specific temperature, while the negative (NTC) and non-target lizard DNA remained as a flat line. Results were analysed using a PC version of the software Genie Explorer version 2.0.7.11 in the blue channel.

2.4.4 Evaluation of a gBlock gene fragment for AHG LAMP assay

Gene Fragment (gBlocks) are targeted synthetic oligonucleotides originally developed to be used as standards in real-time PCR reactions [32] but are equally applicable to LAMP assays as controls to monitor assay performance as synthetic positive controls [14]. To develop a consistent positive control for use in the LAMP assay, a 186 bp gBlock gene fragment (Integrated DNA Technologies, Iowa, USA), modified from the complete fragment used for the assay, was designed for use as a synthetic DNA positive control. This synthetic fragment consisted solely of concatenated LAMP primers separated by runs of "ccc", to increase the overall Tm of the fragment (Table 2). To evaluate detection sensitivity, the copy number was determined, and a ten-fold serial dilution (1:10) of the gBlock was prepared as outlined in [11]. Sensitivity of the serially diluted gBlock was tested in the Genie III following the same AHG LAMP assay amplification conditions (as in section 2.4.3) with the amplification time increased from 25 minutes to 35 minutes, with this longer time included to allow detection of the gBlock present at very low concentrations. Following this, another LAMP run was

conducted to determine the best dilution to be used as a positive control in the LAMP assay. The same five-fold serial dilution (10 ng/µL to 3.2 x 10^{-3} ng/µL) of high-quality AHG tissue DNA (R109520, used previously) with up to six dilutions was used as a template to compare amplification time with a standard amount of one million copies (1 x 10^{5} copies/µL) of gBlock.

2.5 Analytic sensitivity of the AHG LAMP assay

To test the sensitivity of the LAMP assay a five-fold serial dilution of high-quality DNA extract of two biological replicates of AHG scat samples (collected from Dampier, Western Australia) and two biological replicates of AHG tissue samples (R109230 and R109520, Table 1) were prepared using Ultrapure water (Invitrogen, Life Technologies, Australia). Starting DNA concentrations of all four samples were quantified using a Qubit 2.0 Fluorometer (Invitrogen, Life Technologies, Australia) following the manufacturers protocol. All four AHG DNA samples were serially diluted (total eight dilutions) from 10 ng/µL to 1.28 x 10^{-4} ng/µL (1:1 to 1:78125). Sensitivity of the LAMP assay was tested using the serially diluted DNA in the Genie III following the same AHG LAMP assay amplification conditions (as in section 2.4.3). The time of amplification and anneal derivative temperature were recorded for all 8 dilutions. To further explore any differences between DNA obtained from AHG tissue compared with DNA obtained from scats LAMP amplification times and anneal derivative temperature were compared using Student's T-tests in Excel (365 MSO, Version 2503).

2.6 Specimens examined for field testing and lab validation of field samples

In total, 108 samples were tested for the AHG LAMP assay in the field. These samples consisted of 100 scats (S1 Supplementary Table) collected from multiple Western Australian field locations: Barrow Island (n = 49), Dampier (n = 17), Karratha (n = 33) and Perth (n = 1); as well as eight tissue samples collected from Karratha, Australia (n = 4) and Batam, Indonesia (n = 4). DNA was extracted from both scats and tissue samples using the in-field QE DNA extraction method using the Genie III. The AHG LAMP assay was performed (as in section 2.4.3). Amplification time and anneal derivative temperature of all scats, tissues, gBlock (1 x 10^6 copies/µL) synthetic positive control, and negative no template control, were recorded.

All 108 non-destructive QE DNA extracts, and 100 vials containing the other two-thirds / half of the field collected lizard scats were shipped to the laboratory on dry ice. These were then processed for AHG LAMP assay in the laboratory to validate AHG positive samples. Note that 1 µL loops were used to transfer template into master-mix for field testing whereas 1 µL pipette tips were used for adding template in the laboratory. High-quality DNA was extracted from the 100 dry lizard scat samples using the QIAamp® Fast DNA Stool Mini kit (Qiagen, Australia). These DNA extracts were used for further confirmation for species identification through ND2 DNA barcoding (S1 Supplementary Table) using methods outlined above (section 2.3), and primers GeckoND2-F / M1123R.

3. RESULTS

3.1 AHG LAMP assay design and optimisation

Novel LAMP primers were developed to target a 231 bp amplicon for AHG for the mitochondrial ND2 locus (Table 2, Figure 1). Assessment of the primer ratio (F3/B3: FIP/BIP: Floop/Bloop) for this assay found the optimal ratio to be 1:6:3, with final concentrations of 0.4 µM, 2.4 µM and 1.2 µM for the F3/B3, FIP/BIP and Floop/Bloop primers respectively.

Figure 1. AHG LAMP primer alignment. Arrows indicate primer direction; Loop primers are underlined; FIP (5'-3') is made by combining F1 (reverse compliment) and F2; BIP (5'-3') is made by combining B1 (reverse compliment) and B2. Grey-shading indicates AHG, the target species, while non-target gecko species are unshaded. AHG DNA sequences were obtained from [30], while non-target DNA sequences were newly generated. AHG group letters (A-D) are from [30].

3.2 DNA barcoding of specimens tested in the LAMP assay

Species-level identifications of lizard samples examined in the current study were confirmed through DNA barcoding of the ND2 locus (Table 3, Figure 2, S1 Supplementary Table). Relationships among AHG samples were also examined using a haplotype network (Figure 3), which indicated that most Australian AHG samples were very closely related, with a small number of Australian and intercepted specimens being more genetically divergent. Interestingly, some of the Cocos Island (Australian territory) samples matched the most common Australian haplotype found, while others belonged to a highly divergent lineage (>6% different), that had not previously been genetically characterised ("Group E" on Figure 3).

Figure 2. Maximum-likelihood tree of relationships between *Hemidactylus frenatus* (AHG, in bold, GenBank reference sequences) with lizard species collected from Western Australia.

Figure 3. Haplotype network of all AHG sequences obtained from GenBank and generated in the current study. Letters refer to previously recognised genetic groups of AHG (following clade naming system in [30]), apart from 'E' (Cocos Island, territory of Australia), which is newly reported here. Australian samples examined in the current study are shown in green. Red samples were intercepted in transit to Barrow Island, Australia.

3.3 DNA extractions

High-quality DNA extracted using the QIAamp® Fast DNA Stool Mini kit yielded enough DNA from AHG scats for downstream application, while the non-destructive DNA extraction method from scats and tissues using QuickExtract™ was found to be suitable for conducting LAMP assays in the field. All in-field QE DNA extracts from tissue samples, n=18 (Table 1) amplified within the expected time range 10 ± 2.8 minutes (average ± SD). The anneal derivative temperature for all the samples was 85.3 ± 0.2 ^{o}C.

Additionally, three in-field QE DNA extracts from scat samples were tested both undiluted (1:1) and diluted at 1:10, 1:20 and 1:50 (Figure 4 & S2 Supplementary Data). All four DNA dilutions amplified within the expected time range; with 1:1 in 11.75 ± 1.75 minutes, 1:10 in 12.3 ± 1.9 minutes, 1:20 in 16 ± 5.2 minutes and 1:50 in 11.9 ± 0.5 minutes. From these results we recommend using DNA at 1:1, because amplification times were not substantially shorter following dilution. All the samples tested produced an anneal derivative temperature at ~85.5 ^{o}C, which was not affected by DNA dilution.

Figure 4. Optimal method for obtaining in-field DNA extracts in the field. **a)** Scats, 1/3 approx. 3 x 3 mm (left), 1/2 approx. 3 x 5 mm (right). **b)** The same scats in 1.5 ml tubes, pre-DNA extraction. **c)** Extracted DNA (100 µL QuickExtract^{TM}) (1:1) subsequently diluted (1:10, 1:20 and 1:50) using water.

3.4 Performance of the LAMP assay

The AHG LAMP assay produced amplification from AHG DNA from tissue samples in an average of 10.0 ± 2.7 minutes (Figure 5a and 5c, Table 1), with an anneal derivative temperature at 85.4 ± 0.2 ^{o}C (Figure 5b and 5d, Table 1); with amplification in under 25 minutes considered as positive. The non-template control in all the LAMP runs tested did not amplify confirming the absence of primer dimer, and that there were no false positive amplifications due to primer interactions or reagent contamination (Figure 5a and 5c).

Figure 5. Amplification and anneal derivative temperature profiles for the optimised AHG LAMP assay using 1:6:3 primer ratio. **a)** Positive amplification profile of seven AHG tissue samples within 6 to 13 minutes (in-field QuickExtract DNA extracts). Negative, no amplification (flat line, blue). **b)** Anneal derivative temperature is ~85.4 ^{o}C for all 7 AHG tissue DNA. **c)** Positive amplification profile of 7 AHG scat samples within 10 to 14 minutes (high-quality Qiagen DNA extracts). Negative, no amplification (flat line, blue). **d)** Anneal derivative temperature for scat and tissue samples are the same at 85.4 ^{o}C. **e)** Amplification profile of five-fold dilution of AHG tissue DNA (R109520) up to 6 dilutions ranging from 10 ng/µL to 3.2 x 10^{-3} ng/µL (amplification time from highest to lowest DNA concentration was 8 to 12 minutes) and gBlock 1 x 10^5 copies/µL, at 10 minutes (pink). Negative, no

amplification (blue). **f)** Anneal derivative profiles of LAMP amplicons with similar peaks, 85.4 ^{o}C for AHG DNA and 85.5 ^{o}C for gBlock (pink).

"Blind" sample LAMP testing of the 108 tissue and scat samples was conducted in-field producing positive amplification only from AHG tissue and scats (n=38, S1 Supplementary Table). Subsequent laboratory testing showed eight additional AHG scats (21% of total scats identified as AHG) were not detected through the initial in-field DNA extractions by LAMP, with these samples requiring laboratory high-quality DNA extractions to produce enough DNA for secondary LAMP testing or DNA sequencing and barcoding identification (Table 3, S1 Supplementary Table). Generally, 45% of the field collected lizard scats could not be amplified in the laboratory by PCR for DNA barcoding. Importantly, none of the non-target lizard scats (five other species) amplified in the field or laboratory, including all forty-nine scat samples tested from Barrow Island (S1 Supplementary Table).

Table 3. Field validation of AHG LAMP assay on lizard scat samples. AHG samples indicated by grey shading. Species identifications were made through positive LAMP amplification and/or subsequent laboratory DNA barcoding of ND2 (see S1 Supplementary Table).

**Species**	**Collected Mainland, Australia**	**Collected Barrow Island, Australia**	**LAMP Positive (in field)**	**LAMP Negative (in field)**
Hemidactlyus frenatus	38	0	30	8
Cryptoblepharus plagiocephalus	0	3	0	3
Gehyra pilbara	1	11	0	12
Gehyra sp.	1	0	0	1
Gehyra variegata	0	5	0	5
Heteronotia binoei	1	0	0	1

3.5 Evaluation of AHG gBlock gene fragment

The detection sensitivity of AHG gBlock gene fragment evaluated for templates ranging from ~100 million copies down to ~10 copies was quite high, with as low as 1 x 10 copies/µL detected within 15 minutes. From the amplification profile 100,000 copies (1 x 10^5 copies/µL) of gBlock amplified in 10 minutes which equates to ~2 ng/µL of AHG DNA (Figure 5e). Based on the sensitivity test of AHG gBlock 1 x 10^6 copies/µL of gBlock amplified in 8 minutes. The neat DNA concentration, prior to dilution (10 ng/µL), amplified in 8 minutes (Figure 5e). Hence the recommended gBlock dilution would be 1 x 10^6 copies/µL for use as synthetic positive control for future LAMP runs. The anneal derivative

profiles of LAMP amplicons show two very similar peaks, 85.4 ^{o}C for AHG DNA and 85.5 ^{o}C for gBlock (Figure 5f).

The gBlock 1 x 10^6 copies/µL was used in all the LAMP runs as a synthetic positive control to monitor the performance of the assay in the field. Across eighteen LAMP runs the time for positive amplification was consistent, amplifying in 12 ± 0.6 minutes with an anneal derivative temperature of 85 ± 0.3 ^{o}C. Furthermore, the negative control which had no DNA template (NTC) did not amplify in any of the eighteen LAMP runs conducted, showing a flat line both in the amplification and anneal derivative profiles confirming the absence of contaminants in the reagent mix. The results of AHG LAMP assay from both the field testing and laboratory assays showed similar results, demonstrating the robustness of the assay.

3.6 Sensitivity of LAMP assay

The sensitivity of the AHG LAMP assay was tested on five-fold serial dilutions of two biological replicates of each AHG tissue and scat DNA. The time to amplify DNA increased as DNA template concentrations reduced (Figure 6). All eight DNA dilutions from tissue samples produced positive amplification curves with the highest DNA concentration (10 ng/µL) amplifying in 8 minutes compared with the scat DNA which amplified in 11 ± 1.4 minutes (Figure 6). The LAMP test was sensitive in detecting DNA from tissue samples down to the lowest level 1.28 x 10^{-4} ng/µL (0.28 picogram) of DNA in 22 minutes. Sensitivity was lower for DNA dilutions of scat samples, with amplification up to only four dilutions (8 x 10^{-2} ng/µL) in 14 minutes (Figure 6). T-tests (S2 Supplementary Data) showed a significant ($p < 0.02$) difference between amplification times for DNA derived from tissue

and from scats, while there was no significant difference observed for their anneal derivative profiles ($p > 0.25$).

Figure 6. AHG LAMP sensitivity test for a five-fold serial dilution of DNA samples from two AHG tissue (R109230 and R109520) (circles) and two AHG scat (from Dampier, Western Australia) (triangles). Amplification times are shown for all 4 DNA samples with DNA concentration ranging from highest (10 ng/µL) to lowest (1.28 x 10^{-4} ng/µL). All eight DNA dilutions of tissue samples amplified (10 ng/µL DNA amplified in 8 minutes and 1.28 x 10^{-4} ng/µL in 22 minutes). DNA dilutions of scat samples only amplified up to four dilutions (10 ng/µl DNA amplified in 10 and 12 minutes and 8 x 10^{-2} ng/µL in 14 minutes).

4. DISCUSSION

This study designed and optimised a new LAMP assay for the detection of an invasive vertebrate species. The assay was successfully applied to both tissue and scat AHG samples (Tables 1 and 3). These samples represented much of the known AHG genetic diversity (compared with ND2 sequences from [30]), with some specimens from AHG "Group E" being more than 6% genetically divergent from other groups (Figure 3). AHG is known to possess highly distinct genetic lineages, and likely represents a species complex [30, 33]. The presence of two highly divergent genetic lineages amongst our samples indicates that there have likely been at least two independent introduction events to the Cocos (Keeling) Islands.

The new LAMP assay performed well on all samples tested including those from "Group E", allowing all positive AHG samples to be rapidly identified, apart from a small number of

field-collected scat samples which yielded low amounts of DNA. Unfortunately, we did not have access to specimens of any congeneric species to experimentally test the performance of the AHG LAMP assay on other *Hemidactylus* species. Another closely related species, *Hemidactylus platyurus*, occurs north of Australia, however this species (represented by GenBank EU268352) is 36% different for the section of ND2 used in the LAMP assay, so would unlikely amplify.

We designed and optimised a synthetic DNA positive control (gBlock) for use in the AHG LAMP assays following [11]. This synthetic DNA is beneficial in: (i) providing a consistent control to allow tracking of the performance of LAMP assays across runs, and (ii) providing a relatively high amount of control DNA compared with DNA extractions from gecko scat specimens. Scat samples in general produce very low DNA yields [1] due to the small amount of DNA contained within scats. DNA is present at low levels in scat samples compared to blood or tissue samples [34], and can be further reduced by unfavourable environmental conditions with DNA degrading over time [35]. Therefore, the freshest samples should be used when possible. If the scats are not assayed immediately, they can be preserved frozen (approx. -20 ^{o}C) to reduce the rate of DNA degradation. For field collected scat samples, the LAMP assay reduces the risk associated with storage of scats, by providing an in-situ assay that can be completed immediately as required.

When working with scats, the risk of cross contamination is increased due to the low amount of target DNA contained within scats [34]. To reduce this risk, additional precautions should be taken when field collecting scats. The items used to collect samples should be disposable or washed and sterilised between each sample to avoid cross-contamination. Similarly, the

amplicons produced during LAMP assays could result in potential cross contamination between samples if precautions, such as not opening LAMP reaction tubes post amplification, are not followed. For this reason, disposable items should also be used to avoid transferring amplified DNA into the pre-amplification working space, and negative no-template controls should always be included to test for possible LAMP reagent contamination [23].

Amplification of the LAMP assay was found to become slower in a predictable manner as DNA template concentrations reduced. At low DNA concentration of AHG tissue <0.001 ng/µl, the average LAMP amplification time was less than 15 minutes (Figure 6). Similarly, the average LAMP amplification time of gecko scat DNA at low concentrations, <1 ng/µl was also less than 15 minutes (Figure 6). The difference between the lower amplification threshold of tissue and scat samples is likely due to the presence of non-target DNA (e.g., bacteria, digested food etc.) in the scat samples overinflating the overall DNA quantification measurements, with scats also known to have degraded DNA and amplification inhibitors [1, 35].

The amount of the scat used in DNA extraction also appears to influence the quality and quantity of DNA extracted. If whole scat size were used (rather than the recommended one half or third), amplification times were found to increase, likely due to amplification inhibitors such as humic acid present in the scats, which is known to slow LAMP reactions [36]. Other molecular diagnostic approaches, such as DNA barcoding and real-time PCR, are known to be even more sensitive to the presence of inhibitors and are unlikely to amplify from crude (scat) DNA extracts [1, 36]. This, combined with the ease of use, and ability to

rapidly amplify and detect positive targets in portable laboratory / in-field compatible devices are the major advantages LAMP has over other molecular approaches [36].

The LAMP assay outlined here provides a reliable screening tool for the detection of AHG in the field, detecting 100% of AHG tissue samples, and 79% of field scat samples from traces (scats) of AHG. However, as when using any molecular approach, a positive AHG detection should always be verified, particularly if the detection is in an unusual or previously undocumented location. Verification can be performed through using standard DNA barcoding methods, as conducted here. Our study provides new ND2 DNA barcode sequences for an additional twenty-five gecko and skink species present in Western Australia, which greatly improves resources available for molecular identification of these small lizards in Australia. However, verification through DNA barcoding is relatively slow, requiring a turnaround time of days to weeks, compared with LAMP which can produces results in approximately one hour. Therefore, it may be preferable to verify positive LAMP results by increased sampling efforts, through LAMP confirmation of additional samples from the area the detection was made.

The AHG LAMP assay was operationalised in 2022 and is now widely used by Chevron Australia in their biosecurity operations in Australia. This new tool provides accurate rapid diagnosis of intercepted AHG (e.g., Table 1). In August 2023, a scat was collected that was suspected of originating from an AHG, detected on a half-height container during a quarantine inspection at the border of Barrow Island. Following in-situ analysis of the scat using the new AHG LAMP assay the scat was identified as originating from an AHG. A rapid decision was made to reject the cargo and vessel from Barrow Island. Rejecting cargo

and vessels from any port is an expensive operation that is usually met with resistance from stakeholders. The AHG LAMP assay has reduced the subjectivity associated with this decision, which ultimately enhanced cooperation from stakeholders, leading to a quick response. The scat was subsequently sent to Perth for validation of identification through DNA barcoding (as in section 2.3) with DNA sequencing results confirming the LAMP assay identification of an AHG with 100% confidence.

ACKNOWLEDGEMENTS

Scat samples from Barrow Island were sourced by Murdoch University under animal ethics permit number R2859/16. Scats samples from Dampier were provided by Chevron Australia. Tissue samples were provided by both the Western Australian Museum and Helix Molecular Solutions. These tissue samples were obtained from preserved specimens from the WAM, or through a Cadaver and Tissue Usage Notification form.

S1 Supplementary Table. Samples tested in-field: n=100 scats, n=8 tissue samples. Species identify determined through positive LAMP result and/or DNA barcoding.

S2 Supplementary Data. T-test comparisons of LAMP amplification times and anneal derivatives from scat and tissue DNA samples. LAMP optimisation runs for proportion of scat sample extracted, and scat DNA template dilutions in water (1:1, 1:10, 1:50).

Data Availability Statement: All relevant data are within the paper and supporting information files (S1 Supplementary Table & S2 Supplementary Data). ND2 DNA sequences from this study have been accessioned in GenBank (PQ390829 - PQ390908).

Funding: This study was supported through Murdoch University grant MU-112020, and by the State Government of Victoria (Agriculture Victoria Research). This study was funded by the Gorgon Project. The Gorgon Project is operated by an Australian subsidiary of Chevron and is a joint venture of the Australian subsidiaries of Chevron (47.3%), ExxonMobil (25%), Shell (25%), Osaka Gas (1.25%), MidOcean Energy (1%), and JERA (0.417%). The funders had no role in study design, data collection and analysis, decision to publish, or preparation of the manuscript.

Competing interests: The authors declare that no competing interests exist.

AUTHOR CONTRIBUTIONS

Conceptualization: Melissa Thomas.

Data curation: Arati Agarwal, Mark Blacket.

Formal analysis: Arati Agarwal, Mark Blacket.

Funding acquisition: Brendan Rodoni, Mark J. Blacket, Simon McKirdy, Melissa Thomas.

Investigation: Arati Agarwal, Mark Blacket, Yvette Hitchen

Methodology: Arati Agarwal, Mark Blacket, Yvette Hitchen

Resources: Yvette Hitchen, Paul Doughty

Supervision: Arati Agarwal, Melissa Thomas, Yvette Hitchen**,** Mark Blacket.

Writing – original draft: Arati Agarwal, Melissa Thomas, Mark Blacket.

Writing – review & editing: Arati Agarwal, Melissa Thomas, Yvette Hitchen, Paul Doughty, Simon McKirdy, Brendan Rodoni, Mark Blacket.

REFERENCES

[1] Waits LP, & Paetkau D. Noninvasive genetic sampling tools for wildlife biologists: A review of applications and recommendations for accurate data collection. Journal of Wildlife Management. 2005; 69, 1419-1433.

[2] Croose E, Birks JDS, O'Reilly C, Turner P, Martin J, & Macleod ET. Sample diversity adds value to non-invasive genetic assessment of a pine marten (*Martes martes*) population in Galloway Forest, southwest Scotland. Mammal Research. 2016; 61, 131-139.

[3] Rajpoot A, Kumar VP, Bahuguna A, & Rasaily SS. Preliminary genetic documentation of snake species through shed skin from Uttarakhand, India: A non-invasive genetic sampling approach. Journal of Wildlife and Biodiversity. 2021; 5; 81-91.

[4] Berry O, Sarre SD, Farrington L, & Aitken N. Faecal DNA detection of invasive species: the case of feral foxes in Tasmania. Wildlife Research. 2007; 34, 1-7.

[5] Waits LP. Using noninvasive genetic sampling to detect and estimate abundance of rare wildlife species. In: THOMAS, W. L. (ed.) Sampling rare or elusive species: concepts, designs, and techniques for estimating population parameters. Washington, D. C., U.S.A.: Island Press 2004.

[6] Piggott MP, & Taylor AC. Remote collection of animal DNA and its applications in conservation management and understanding the population biology of rare and cryptic species. Wildlife Research. 2003; 30, 1-13.

[7] Pennell MW, Stansbury CR, Waits LP, & Miller CR. Capwire: a R package for estimating population census size from non-invasive genetic sampling. Molecular Ecology Resources. 2013; 13, 154-157.

[8] Baumgardt JA, Goldberg CS, Reese KP, Connelly JW, Musil DD, Garton EO, & Waits LP. A method for estimating population sex ratio for sage-grouse using noninvasive genetic samples. Molecular Ecology Resources. 2013; 13, 393-402.

[9] Idaghdour Y, Broderick D, Korrida A. Faeces as a source of DNA for molecular studies in a threatened population of great bustards. Conservation Genetics. 2003; 4:789–792.

[10] Notomi T, Okayama H, Masubuchi H, Yonekawa T, Watanabe K, Amino N, & Hase T. Loop-mediated isothermal amplification of DNA. Nucleic Acids Research. 2000; 28, e63.

[11] Agarwal A, Cunningham JP, Valenzuela I, & Blacket MJ, A diagnostic LAMP assay for the destructive grapevine insect pest, phylloxera (*Daktulosphaira vitifoliae*). Scientific Reports. 2020; 10:1-10.

[12] Rako L, Agarwal A, & Blacket MJ. A LAMP (Loop-mediated isothermal amplification) test for rapid identification of Khapra beetle (*Trogoderma granarium*), Pest Management Science. 2021; 77: 5509-5521.

[13] Rako L, Agarwal A, Eow L, Roberts JMK, Rodoni BC & Blacket MJ. LAMP (Loop-mediated isothermal amplification) assay for rapid identification of Varroa mites, Scientific Reports. 2023; 13: 11931.

[14] Agarwal A, Rako L, Schutze MK, Starkie ML, Tay WT, Rodoni BC, & Blacket MJ. A diagnostic LAMP assay for rapid identification of an invasive plant pest, fall armyworm *Spodoptera frugiperda* (Lepidoptera: Noctuidae). Scientific Reports. 2022; 12(1): 1116.

[15] Hoskin CJ, The invasion and potential impact of the Asian House Gecko (*Hemidactylus frenatus*) in Australia. Austral Ecology. 2011; 36, 240-251.

[16] Hopkins JM, Higgie M, & Hoskin CJ. Calling behaviour in the invasive Asian house gecko (*Hemidactylus frenatus*) and implications for early detection. Wildlife Research. 2020; 48, 152-162.

[17] Cole NC, Jones CG, & Harris S. The need for enemy-free space: The impact of an invasive gecko on island endemics. Biological Conservation. 2005; 125, 467-474.

[18] Barnett LK, Phillips BL, & Hoskin CJ. Going feral: Time and propagule pressure determine range expansion of Asian house geckos into natural environments. Austral Ecology. 2017; 42, 165-175.

[19] Barnett LK, Phillips BL, Heath ACG, Coates A, & Hoskin CJ. The impact of parasites during range expansion of an invasive gecko. Parasitology. 2018; 145, 1400-1409.

[20] Chapple DG, Knegtmans J, Kikillus H, & Van Winkel D. Biosecurity of exotic reptiles and amphibians in New Zealand: building upon Tony Whitaker's legacy. Journal of the Royal Society of New Zealand. 2016; 46, 66-84.

[21] Scott JK, McKirdy SJ, Van der Merwe J, Green R, Burbidge AA, Pickles G, Hardie DC, Morris K, Kendrick PG, Thomas ML, Horton KL, O'Connor SM, Downs J, Stoklosa R, Lagdon R, Marks B, Nairn M, & Mengersen K. Zero-tolerance biosecurity protects high-conservation-value island nature reserve. Scientific Reports. 2017; 7: 772.

[22] Van der Merwe J. Barrow Island quarantine: beyond best practice. Abu Dhabi International Petroleum Exhibition and Conference. Abu Dhabi: Society of Petroleum Engineers. 2015.

[23] Blacket MJ, Agarwal A, Zheng L, Cunningham JP, Britton D, Schneider I, & Rodoni BC. A LAMP assay for detection of *Bactrocera tryoni* Queensland fruit fly (Diptera: Tephiritidae). Scientific Reports. 2020; 10: 9554.

[24] Starkie ML, Fowler EV, Zhu X, et al. Loop-mediated isothermal amplification (LAMP) assays for detection of the New Guinea fruit fly *Bactrocera trivialis* (Drew) (Diptera: Tephritidae). Scientific Reports. 2022; 12, 12602.

[25] Heers T, van Neer A, Becker A, Grilo ML, Siebert U, Abdulmawjood A. Loop-mediated isothermal amplification (LAMP) assay - A rapid detection tool for identifying red fox (Vulpes vulpes) DNA in the carcasses of harbour porpoises (*Phocoena phocoena*). PLoS One. 2017; 12(9):e0184349.

[26] Sistrom MJ, Hutchinson MN, Hutchinson RG, & Donnellan SC. Molecular phylogeny of Australian *Gehyra* (Squamata: Gekkonidae) and taxonomic revision of *Gehyra variegata* in south-eastern Australia. Zootaxa. 2009; 2277, 14-32.

[27] Kumar S, Stecher G, and Tamura K. MEGA7: Molecular Evolutionary Genetic Analysis version 7.0 for bigger datasets. Molecular Biology and Evolution. 2016; 33: 1870-1874.

[28] Bandelt H, Forster P, & R¨ohl A. Median-joining networks for inferring intraspecific phylogenies. Molecular Biology and Evolution. 1999; 16(1), 3748.

[29] Leigh JW, & Bryant D. Popart: full-feature software for haplotype network construction. Methods in Ecology and Evolution. 2015; 6, 1110–1116.

[30] Agarwal I, Jablonski D, & Bauer AM. The identity and probable origin of the *Hemidactylus* geckos of the Maldives. Herpetological Journal. 2019; 29: 230-236.

[31] OligoAnalyzer Tool. https://sg.idtdna.com/calc/analyzer

[32] Conte J, Potoczniak MJ, Tobe SS, Using synthetic oligonucleotides as standards in probe-based qPCR. BioTechniques. 2018; 64: 177-179.

[33] Rocha S, Trinks A, Harris DJ, Larson G and Cheke AS. The Global and Western Indian Ocean Dispersal of House Geckos From Asia Using Historical and Mitochondrial DNA Perspectives. Frontiers in Ecology and Evolution. 2022; 10, 791762.

[34] Taberlet P, Waits LP, & Luikart G. Noninvasive genetic sampling: look before you leap. Trends in Ecology & Evolution. 1999; 14, 323-327.

[35] Piggott MP. Effect of sample age and season of collection on the reliability of microsatellite genotyping of faecal DNA. Wildlife Research. 2004; 31, 485-493.

[36] Nwe MK, Jangpromma N, & Taemaitree L. Evaluation of molecular inhibitors of loop-mediated isothermal amplification (LAMP). 2024; 14, 5916.

Figure
Click here to access/download;Figure;Figure 1.tif

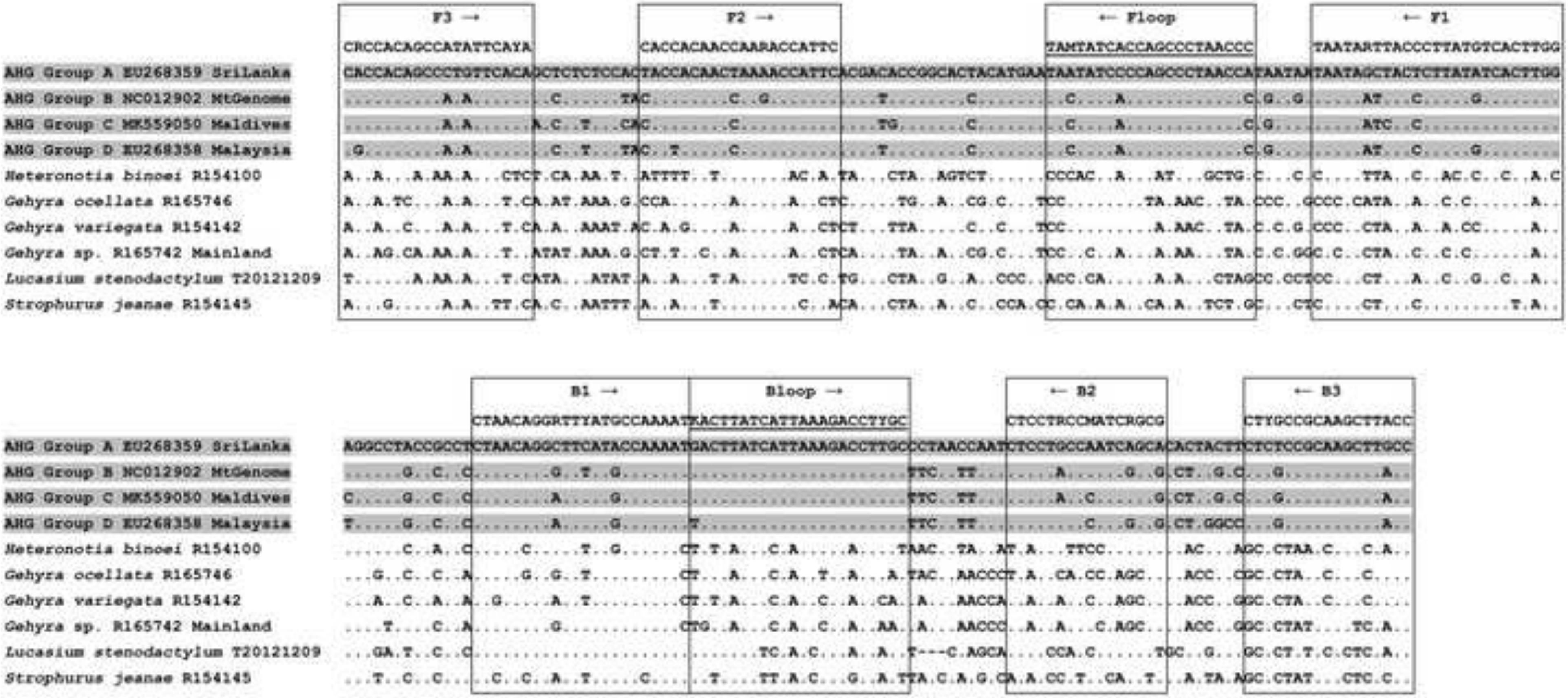

OFFICIAL

Figure Click here to access/download;Figure;Figure 2.tif

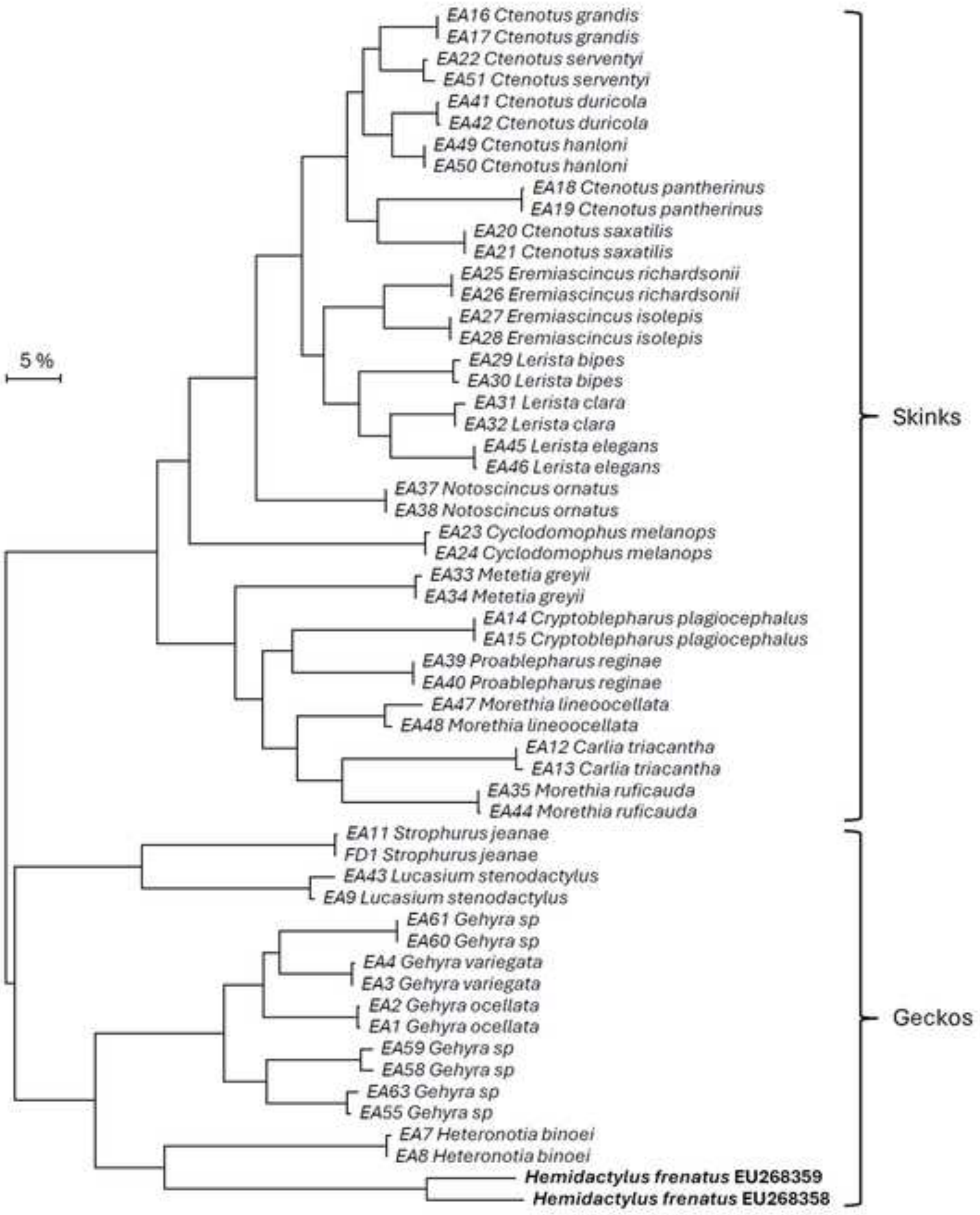

 Click here to access/download;Figure;Figure 3.tif

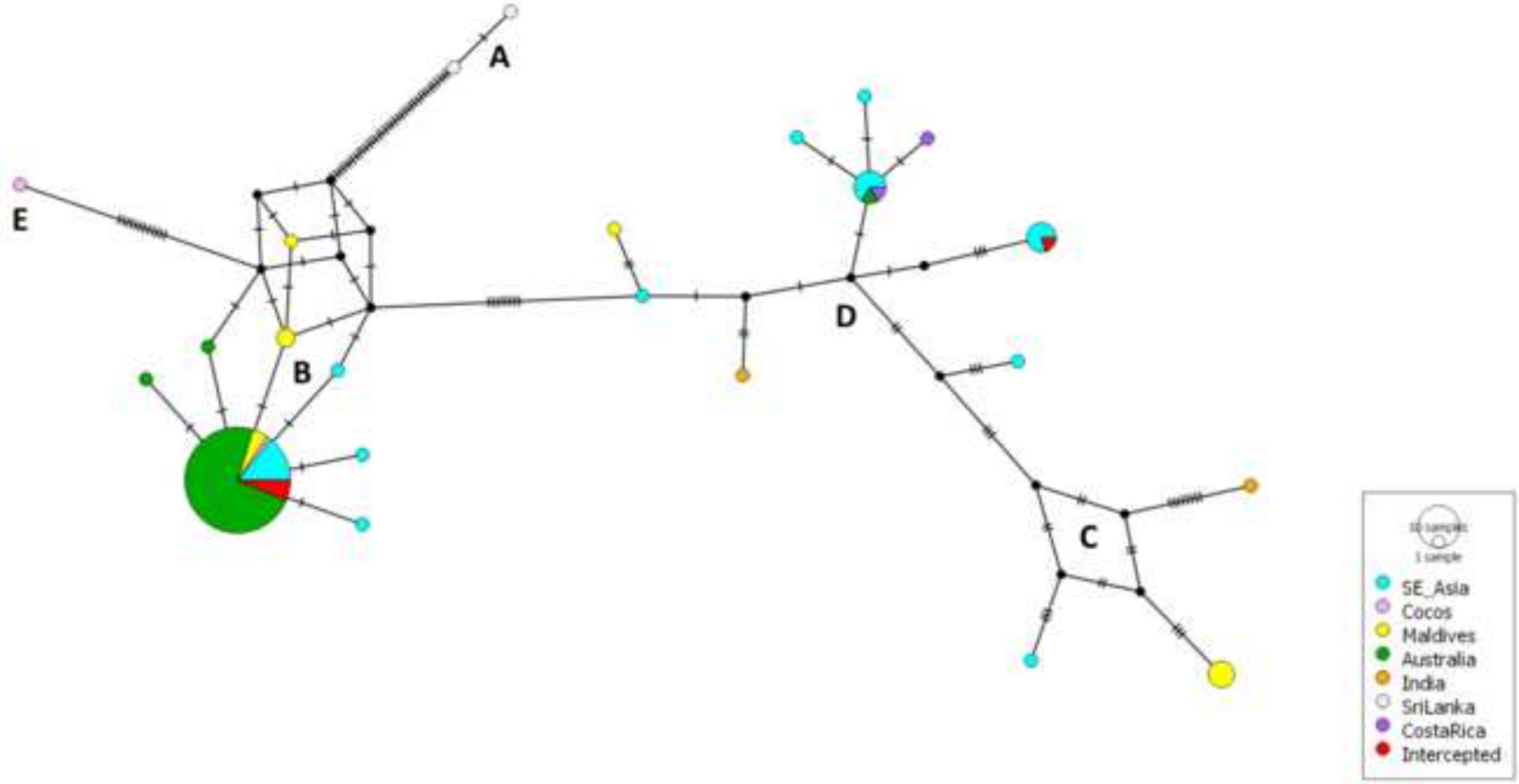

 Click here to access/download;Figure;Figure 4.tif

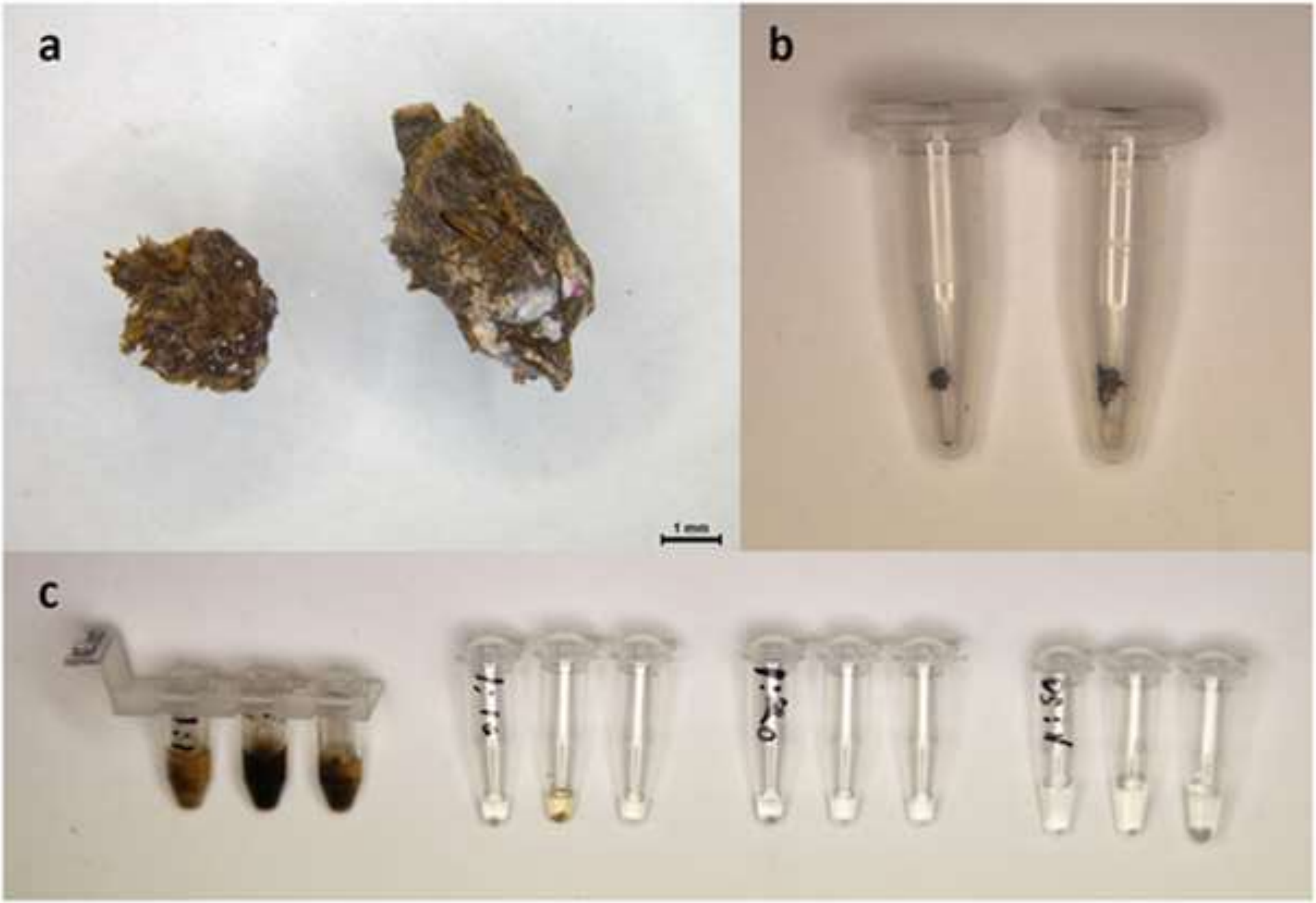

Figure

Click here to access/download;Figure;Figure 5.tif

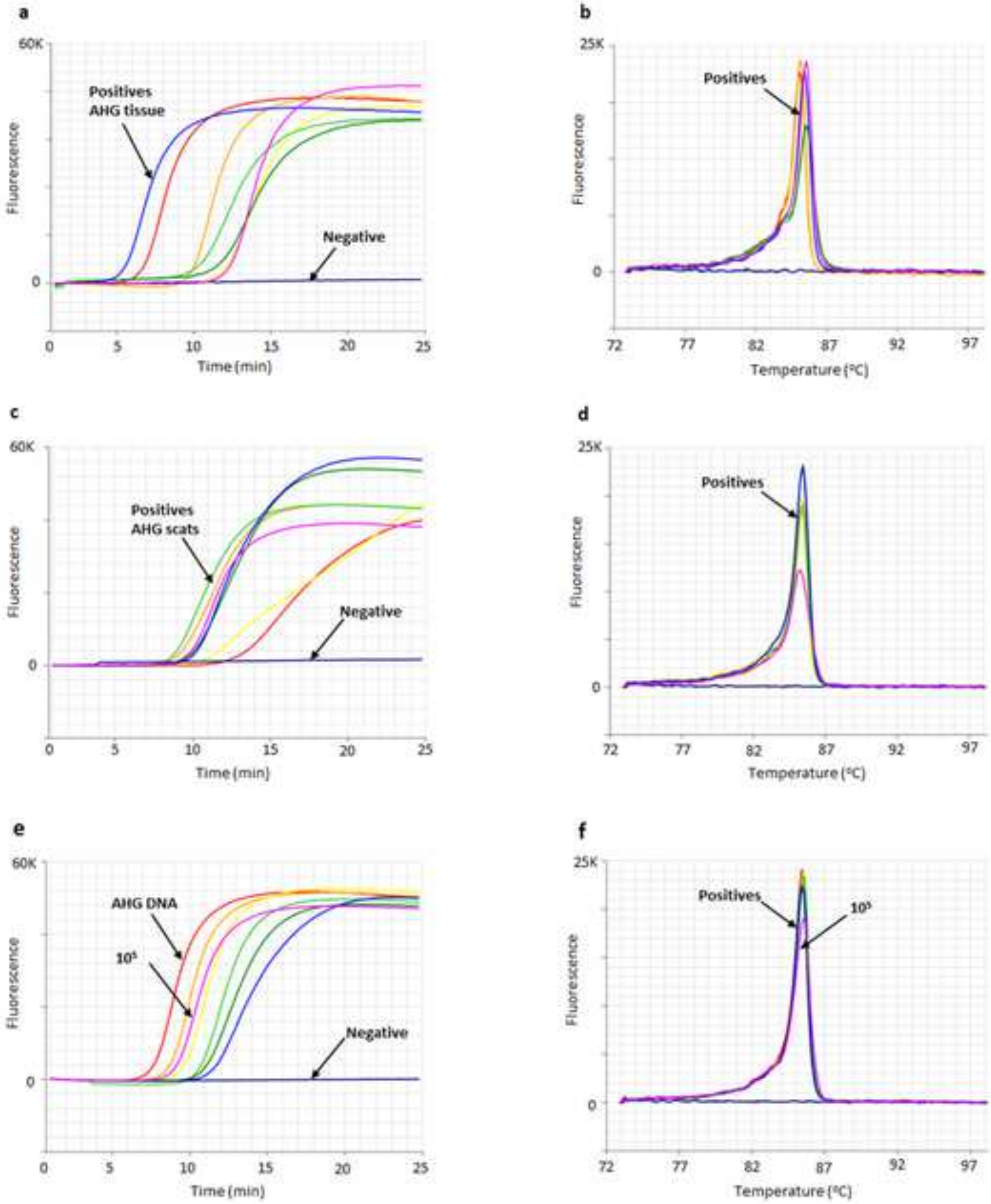

 Click here to access/download;Figure;Figure 6.tif

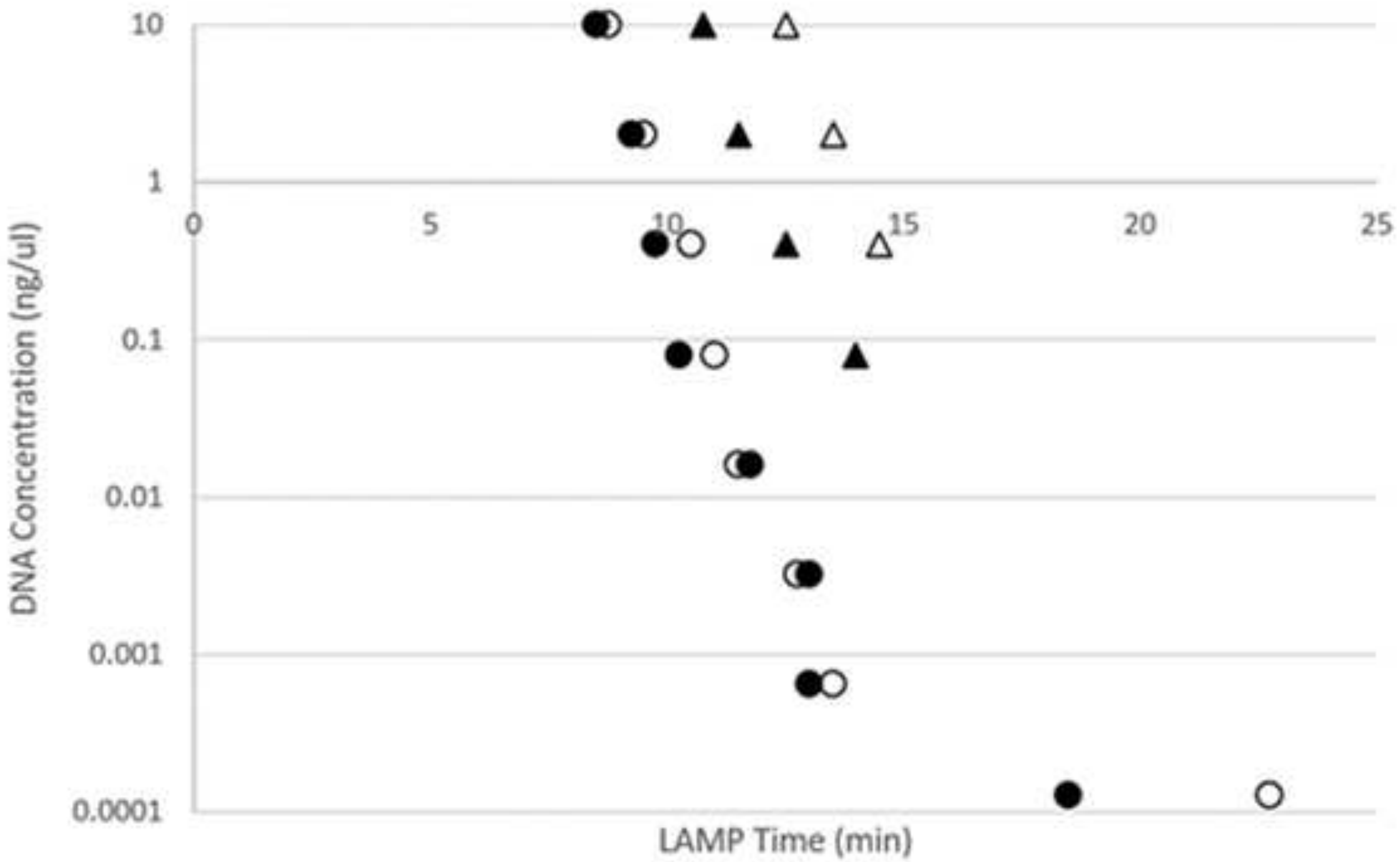

OFFICIAL

A new molecular tool for detection of the highly invasive gecko, *Hemidactylus frenatus*

Arati Agarwal [1], Melissa L Thomas [2], Yvette Hitchen [3], Paul Doughty [4], Simon J. McKirdy [2], Brendan C. Rodoni [1,5], Mark J. Blacket [1*]

[1] Agriculture Victoria Research, AgriBio - Centre for AgriBioscience, 5 Ring Road, Bundoora, VIC 3083, Australia.

[2] Harry Butler Institute, Murdoch University, Perth, WA 6150, Australia

[3] Helix Molecular Solutions, Leederville, WA 6153, Australia

[4] Western Australia Museum, Terrestrial Zoology, Welshpool, WA 6986, Australia

[5] School of Applied Systems Biology, La Trobe University, Bundoora 3083, Victoria, Australia.

**** Corresponding Author:*** Mark Blacket; email: mark.blacket@agriculture.vic.gov.au

Short title: LAMP assay for detection of *Hemidactylus frenatus*

ABSTRACT

The Common House Gecko, *Hemidactylus frenatus*, also known as the Asian House Gecko (AHG), is the most significant invasive gecko globally. Detecting this species can be ~~difficult~~ challenging ~~as~~ because it ~~is similar in appearance to many~~closely resembles other geckos, and is often not directly observed, being cryptic and nocturnal. Traces such as scats, however, are more readily observed than the animal itself. Here, we developed and tested a new diagnostic mitochondrial ND2 LAMP (Loop-mediated isothermal amplification) assay to detect and distinguish AHG from other geckos. ~~The assay was found to be highly sensitive, capable of amplifying very low levels, down to 0.0001 ng/μL of AHG DNA, within 25 minutes.~~ Testing DNA from twenty-five non-target gecko and skink species present in Western Australia demonstrated ~~confirmed~~ the species-specificity of the assay. This new molecular assay was found to be highly specific with amplification in under 15 minutes from AHG DNA. Intraspecific variation did not adversely affect the LAMP assay, with all AHG tissue samples successfully amplifying. Including samples from the Cocos (Keeling) Islands territory of Australia which were >6% genetically divergent from mainland Australian samples, representing a genetic group that was previously unknown, referred to here as AHG “clade E”. The assay was found to be highly sensitive, capable of amplifying AHG DNA at very low levels, down to 0.0001 ng/μL of AHG DNA, within 25 minutes. The new LAMP assay has been fully optimised for in-field use, including development and testing of a non-destructive DNA extraction method for in-field extractions from both AHG tissue and scat samples, as well as a gBlock gene fragment for use as a synthetic positive control. The in-field protocols were tested on 100 field collected scats, from multiple lizard species in Western Australia, demonstrating ~~confirming~~ the AHG specificity of the assay, with amplification successful on 79% of AHG scats tested in-field. While the assay was highly effective~~highly effective~~, scat DNA degradation and inhibitors limited detection in 21% of AHG samples, highlighting

preservation challenges. This new assay has already been applied operationally in the field, providing early detection of AHG, and preventing potential introduction of this species into new areas.

1. Introduction

Non-invasive genetic sampling (NGS) has been widely applied over the last twenty years by wildlife biologists [1]. NGS is when DNA is extracted from animal traces, such as from hair [2], shed skin [3], faecal scats [2, 4], or other sources, without handling, capturing or even observing the individual animal. NGS alleviates many of the difficulties associated with conventional ~~morphological~~ identifications including morphological complexities, cryptic life stages, and globally declining taxonomic expertise. Applications of NGS include detection of rare or endangered species [5], conservation management [6], differentiating between morphologically similar species [1], population estimation (e.g., [7]), and population sex-ratios [8]. NGS has also been applied for monitoring of invasive species [4], however, studies using NGS as an early detection tool for biosecurity purposes remain rare.

One of the primary issues facing NGS of animals, is the quality of trace DNA. Animal faecal scats are one of the most common NGS sources, however scats contain very little host animal DNA and considerable concentrations of inhibitors [1]. The diet of the animal can play a significant role in the success of DNA extraction from scats. For example, insect material in scats is known to have an inhibitory effect on amplification success when DNA concentrations of the target species are low [9]. Furthermore, scat samples are generally collected from wild animals whose scats are likely to have been exposed to a variety of weather conditions which can degrade the DNA prior to the scat being collected. Other factors, such as DNA extraction method and sample preservation can also influence the success of extracting species DNA from scats [1]. These challenges highlight the need for more sensitive molecular techniques capable of detecting low-quality DNA under field conditions.

Loop-mediated isothermal amplification (L~~OO~~AMP) offers a promising alternative to conventional PCR based approaches. LAMP enables rapid amplification of target DNA with high specificity, sensitivity, and robustness under isothermal conditions [10]. ~~For NGS when DNA quality is low, there is a need for new sensitive methods that can effectively and easily be used in the field for rapid molecular diagnostics. LAMP (Loop-mediated isothermal amplification (LAMP) is~~ is ~~a novel molecular method that can amplify the assay target DNA with high specificity, sensitivity, and rapidity under isothermal conditions [10].~~ Assays can be conducted under field conditions ~~on~~ using portable ~~devices~~ equipment and provide ~~providing~~ robust results in less than one hour. ~~Furthermore~~Importantly, LAMP is ~~not~~ relatively tolerant to common ~~particularly sensitive to~~ inhibitors, ~~and~~ is highly efficient, and can sensitively ~~enabling sensitive detection~~detect low concentrations of target DNA (e.g., [11-14~~3~~]), making it ~~potentially~~ particularly suitable for use on NGS sources of DNA~~ such as scats~~. ~~Assay positive controls can be provided by Gene Fragment (gBlocks), being targeted synthetic oligonucleotides which were originally developed to be used as standards in real-time PCR reactions [14], but are equally applicable to LAMP assays as controls to monitor assay performance as synthetic positive controls [15].~~

~~Invasive species can cause catastrophic impacts on their introduced environment [15], and rapid early detection can be critical to stopping potential exotic incursions. Many recent targeted~~~~Invasive species can cause catastrophic impacts on their introduced environment [16]. To enable successful management of invasive species, early detection and robust identification of the species is required. Targeted LAMP assays have been~~being ~~developed to aid early detection of numerous invasive invertebrate species from insect specimens, including fruit flies~~ *~~Bactrocera tryoni~~* ~~[17]~~[16], *~~B. trivialis~~* ~~[18]~~[17]~~, fall armyworm~~

~~*Spodoptera frugiperda* [15]~~[~~14~~]~~, grape phylloxera *Daktulosphaira vitifoliae* [11], and honeybee ectoparasitic *Varroa* mites [13]. However, for many other invasive species, particularly those that are cryptic in nature, signs of the animal are more often detected before the animal itself. NGS LAMP assays have been successfully developed and employed to detect traces of an invasive invertebrate species, from shed larval skins of the Khapra beetle *Trogoderma granarium* [12], and to confirm the the red fox *Vulpes vulpes* [19], as the cause of vertebrate predator scavenging.~~

The Common House Gecko (*Hemidactylus frenatus* Dumeril & Bibron), also known as the ~~the Common House Gecko, otherwise known as the~~ Asian House Gecko (AHG), is one of the world's ~~the~~ most ~~significant~~ successful invasive geckos ~~globally~~ [15~~20~~, 16~~21~~]. In parts of its introduced range, *H. frenatus* has invaded natural habitats where impacts on native geckos have been demonstrated [17~~22~~], or inferred [18~~23~~, 19~~24~~]. This species ~~remains~~ poses an ongoing ~~a constant~~ biosecurity ~~threat~~risk; ~~for example,~~ in New Zealand it ~~can make up to~~accounts for up to 44% of all intercepted herpetofauna ~~intercepts~~ [20~~25~~], ~~while~~ and on a remote Australian island, it is the most ~~common~~ frequently detected gecko ~~species~~ intercepted at the border [21~~26~~]. It ~~is~~ presents particular~~rly~~ ~~threatening~~ risks to island ecosystems, which are ~~are~~ often the last refuges for endangered species [22~~27~~]. Detecting incursions of this species ~~quickly~~ rapidly is important as populations can ~~rapidly~~ quickly achieve high densities that may preclude eradication [18~~23~~].

Currently, ~~the primary method for detecting~~ *H. frenatus* detection relies primarily on ~~is through~~ auditory ~~means, or~~and visual surveys ~~for the animal (or traces of)~~. Males produce distinctive calls ~~*Hemidactylus frenatus* is one of a minority of lizards that use acoustic~~

communication and has a very conspicuous call [1621], but these are limited to specific behavioural contexts and environmental conditions, with typically only adult males calling in the presence of females, and only during warmer months .around sunset and sunrise However, calls are not an ideal candidate for early detection of incursions as they are uttered exclusively by males, primarily by adult males under specific circumstances, with calls being more common in warmer months, at sunrise and before sunset, and in the presence of females [1621]. Visual surveys also require expertise to distinguish for *H. frenatus* from morphologically similar native geckos require expert knowledge as because morphological differences between *H. frenatus* and some native geckos (e.g., *Gehyra variagata* in Australia) can be difficult to differentiate under field conditions. Similarly, scats from different gecko species of geckos can appear almost identical in appearance. For these reasons, scat collections, combined with conventional PCR followed by DNA sequencing, is are often employed used to confirm species presence to identify the presence/absence of this species [2126]. UnfortunatelyHowever, such molecular methods, this molecular method are constrained by has a relatively long turnaround times (approxtypically. 2 to 4 weeks for from sample collection to analysis), which limits their sample delivery, processing, and analysis) for use as an early detection tool.

Development of a targeted LAMP assay for *H. frenatus* could provide a

Formatted: Font: Italic

If a targeted LAMP assay was available, it could enable rapid, specific and field-deployable molecular tool capable of detecting AHG DNA from scats within one hour. detection of AHG from scat DNA, potentially outperforming traditional methods in field settings. A LAMP assay for detection of AHG would provide an alternative rapid, within one hour, accurate

~~identification tool, that a~~Although LAMP is generally less sensitive ~~compared~~ than ~~with~~ real-time PCR, its speed, simplicity, and inhibitor tolerance make it an attractive option for biosecurity surveillance. ~~could be used to amplify from crude (scat) DNA extracts efficiently.~~

LAMP assays have already been developed successfully to aid early detection of several invasive invertebrate species, including fruit flies *Bactrocera tryoni* [23~~16~~], *B. trivialis* [24~~17~~], fall armyworm *Spodoptera frugiperda* [14], grape phylloxera *Daktulosphaira vitifoliae* [11], and honeybee ectoparasitic *Varroa* mites [13]. NGS LAMP assays have also been successfully developed and employed to detect traces of two invasive species, including one vertebrate species; shed larval skins of the Khapra beetle *Trogoderma granarium* [12], and traces of DNA of the red fox *Vulpes vulpes* that scavenged on harbour porpoise carcasses [25~~19~~].

In th~~e present~~is study, we developed and validated a ~~new~~ novel LAMP assay for the detection of ~~the~~ *H. frenatus* (AHG). We used NGS (from small reptile scats) and ~~gecko~~ tissue samples ~~as sources of~~to evaluate DNA ~~and tested the~~assay specificity and sensitivity ~~performance of the new assay~~. ~~Additionally, f~~Field testing ~~of the assay~~ was conducted on ~~lizard~~ scats collected from (i) mainland Australia where *H. frenatus* is established, ~~a known population of AHG occurs,~~ and (ii) ~~offshore on~~ Barrow Island, where ~~AHG it~~the species is currently absent, but ~~AHG it~~represents an ongoing ~~continues to be an~~ incursion threat.

Formatted: Font: Italic

2. MATERIALS AND METHODS

2.1 Specimens Examined

For developing, optimising, and testing the specificity of our LAMP assay, we used 26 tissue samples from the target species (AHG) together with 54 tissue samples from twenty-five Australian native gecko and skink species which co-occur geographically with AHG in Western Australia (Table 1). Samples were sourced by Chevron Australia, Helix Molecular Solutions (Helix), and the Western Australian Museum (WAM). One hundred and twenty lizard scat samples were used for optimising (n=20) and field validating (n=100) the assay, collected from multiple sites in Western Australia (see section 2.7~~below~~ and S1 Supplementary Table).

Table 1. Gecko and skink DNA samples (from tissue) used for development and initial testing of AHG LAMP assay. AHG samples are indicated by grey shading. “N/A” indicates no amplification produced with the new AHG LAMP assay, QE = QuickExtract, Qiagen = DNeasy extraction, Helix = Helix Molecular Solutions, WAM = Western Australian Museum, ND2 = mitochondrial ND2 locus.

	Species	Sample	Source	Extraction method	LAMP Time (min)	LAMP Anneal (ºC)	GenBank Accession (ND2)
1	*Hemidactlyus frenatus*	BS01 Karratha, Australia	Helix	Qiagen	9.25	85.5	PQ390829
2	*Hemidactlyus frenatus*	BS10 Karratha, Australia	Helix	Qiagen	9.00	85.5	PQ390830
3	*Hemidactlyus frenatus*	BS26 Batam, Indonesia	Helix	Qiagen	12.25	85.5	PQ390831
4	*Hemidactlyus frenatus*	BS28 Batam, Indonesia	Helix	Qiagen	15.00	86.2	PQ390832
5	*Hemidactlyus frenatus*	HR18 Intercepted	Helix	Qiagen	8.50	85.3	PQ390833
6	*Hemidactlyus frenatus*	HR19 Intercepted	Helix	Qiagen	8.50	85.2	PQ390834
7	*Hemidactlyus frenatus*	R131793, Kununurra, Australia	WAM	QE	7.25	85.3	PQ390835
8	*Hemidactlyus frenatus*	R131879, Kununurra, Australia	WAM	QE	7.50	85.2	PQ390836
9	*Hemidactlyus frenatus*	R132949, Broome, Australia	WAM	QE	9.75	85.3	PQ390837
10	*Hemidactlyus frenatus*	R132952, Broome, Australia	WAM	QE	10.00	85.3	PQ390838
11	*Hemidactlyus frenatus*	R164914, Truscott Airstrip, Australia	WAM	QE	13.25	85.4	PQ390839
12	*Hemidactlyus frenatus*	R172512, Intercepted	WAM	QE	16.25	85.8	PQ390840
13	*Hemidactlyus frenatus*	R172517, Intercepted	WAM	QE	9.25	85.3	PQ390841
14	*Hemidactlyus frenatus*	R172557, Intercepted	WAM	QE	11.25	85.3	PQ390842
15	*Hemidactlyus frenatus*	R174322, Port Hedland, Australia	WAM	QE	7.50	85.1	PQ390843
16	*Hemidactlyus frenatus*	R174747, Carnarvon, Australia	WAM	QE	7.50	85.3	PQ390844
17	*Hemidactlyus frenatus*	R173044, Cocos (Keeling) Island	WAM	QE	10.75	85.1	PQ390845
18	*Hemidactlyus frenatus*	R173054, Cocos (Keeling) Island	WAM	QE	13.25	85.1	PQ390846
19	*Hemidactlyus frenatus*	R112253, Yamdena Island, Indonesia	WAM	QE	11.75	85.6	PQ390847

20	*Hemidactlyus frenatus*	R109929, Yamdena Island, Indonesia	WAM	QE	13.25	85.6	PQ390848
21	*Hemidactlyus frenatus*	R109377, Banda island, Indonesia	WAM	QE	11.50	85.2	PQ390849
22	*Hemidactlyus frenatus*	R109370, Banda island, Indonesia	WAM	QE	6.50	85.4	PQ390850
23	*Hemidactlyus frenatus*	R109230, Nusa Penida Island, Indonesia	WAM	Qiagen	8.00	85.4	PQ390851
24	*Hemidactlyus frenatus*	R109517, Wokam island, Indonesia	WAM	QE	7.00	85.4	PQ390852
25	*Hemidactlyus frenatus*	R109520, Wokam island, Indonesia	WAM	Qiagen	8.25	85.5	PQ390853
26	*Hemidactlyus frenatus*	R109879, Ambon Island, Indonesia	WAM	QE	6.50	85.4	PQ390854
27	*Gehyra pilbara*	EA01 R165746, Australia	Helix	Qiagen	N/A	N/A	PQ390855
28	*Gehyra pilbara*	EA02 R165753, Australia	Helix	Qiagen	N/A	N/A	PQ390856
29	*Gehyra variegata*	EA03 R154142, Australia	Helix	Qiagen	N/A	N/A	PQ390857
30	*Gehyra variegata*	EA04 R165763, Australia	Helix	Qiagen	N/A	N/A	PQ390858
31	*Heteronotia binoei*	EA07 R154100, Australia	Helix	Qiagen	N/A	N/A	PQ390859
32	*Heteronotia binoei*	EA08 R154114, Australia	Helix	Qiagen	N/A	N/A	PQ390860
33	*Lucasium stenodactylum*	EA09 T20121209.HPZ2-03lucste, Australia	Helix	Qiagen	N/A	N/A	PQ390861
34	*Strophurus jeanae*	EA11 R154145, Australia	Helix	Qiagen	N/A	N/A	PQ390862
35	*Carlia triacantha*	EA12 T20130323.22HFZ2-03CARTRI.01, Australia	Helix	Qiagen	N/A	N/A	PQ390863
36	*Carlia triacantha*	EA13 T20130412.23HPZ2-02CARTRI.01, Australia	Helix	Qiagen	N/A	N/A	PQ390864
37	*Cryptoblepharus plagiocephalus*	EA14 R154110, Australia	Helix	Qiagen	N/A	N/A	PQ390865
38	*Cryptoblepharus plagiocephalus*	EA15 R154113, Australia	Helix	Qiagen	N/A	N/A	PQ390866
39	*Ctenotus grandis*	EA16 R154081, Australia	Helix	Qiagen	N/A	N/A	PQ390867
40	*Ctenotus grandis*	EA17 R154084, Australia	Helix	Qiagen	N/A	N/A	PQ390868
41	*Ctenotus Pantherinus acripes*	EA18 R154151, Australia	Helix	Qiagen	N/A	N/A	PQ390869
42	*Ctenotus Pantherinus acripes*	EA19 R154168, Australia	Helix	Qiagen	N/A	N/A	PQ390870
43	*Ctenotus saxatilis*	EA20 R154092, Australia	Helix	Qiagen	N/A	N/A	PQ390871
44	*Ctenotus saxatilis*	EA21 R154099, Australia	Helix	Qiagen	N/A	N/A	PQ390872
45	*Ctenotus serventyi*	EA22 T20120521.HPZ2A-06.01, Australia	Helix	Qiagen	N/A	N/A	PQ390873
46	*Cyclodomophus melanops*	EA23 R154130, Australia	Helix	Qiagen	N/A	N/A	PQ390874
47	*Cyclodomophus melanops*	EA24 R154160, Australia	Helix	Qiagen	N/A	N/A	PQ390875
48	*Eremiascincus richardsonii*	EA25 R165786, Australia	Helix	Qiagen	N/A	N/A	PQ390876
49	*Eremiascincus richardsonii*	EA26 T20121113.OFZ1-02ERERIC.01, Australia	Helix	Qiagen	N/A	N/A	PQ390877
50	*Eremiascincus isolepis*	EA27 T20120519.HFZ1-03.01, Australia	Helix	Qiagen	N/A	N/A	PQ390878

51	*Eremiascincus isolepis*	EA28 T20120520.HFZ2-02.01, Australia	Helix	Qiagen	N/A	N/A	PQ390879
52	*Lerista bipes*	EA29 R154118, Australia	Helix	Qiagen	N/A	N/A	PQ390880
53	*Lerista bipes*	EA30 R154121, Australia	Helix	Qiagen	N/A	N/A	PQ390881
54	*Lerista clara*	EA31 R154140, Australia	Helix	Qiagen	N/A	N/A	PQ390882
55	*Lerista clara*	EA32 R154166, Australia	Helix	Qiagen	N/A	N/A	PQ390883
56	*Metetia greyii*	EA33 R154144, Australia	Helix	Qiagen	N/A	N/A	PQ390884
57	*Metetia greyii*	EA34 R154173, Australia	Helix	Qiagen	N/A	N/A	PQ390885
58	*Morethia ruficauda*	EA35 R154174, Australia	Helix	Qiagen	N/A	N/A	PQ390886
59	*Notoscincus ornatus*	EA37 R154137, Australia	Helix	Qiagen	N/A	N/A	PQ390887
60	*Notoscincus ornatus*	EA38 R154147, Australia	Helix	Qiagen	N/A	N/A	PQ390888
61	*Proablepharus reginae*	EA39 R154135, Australia	Helix	Qiagen	N/A	N/A	PQ390889
62	*Proablepharus reginae*	EA40 R154136, Australia	Helix	Qiagen	N/A	N/A	PQ390890
63	*Ctenotus duricola*	EA41 T20131114.28HPZ2-01.CTEDUR.01, Australia	Helix	Qiagen	N/A	N/A	PQ390891
64	*Ctenotus duricola*	EA42 T20131106.28HPZ2-04.CTEDUR.01, Australia	Helix	Qiagen	N/A	N/A	PQ390892
65	*Lucasium stenodactylum*	EA43 T20131115.28GPZ1-08.LUCSTE.01, Australia	Helix	Qiagen	N/A	N/A	PQ390893
66	*Morethia ruficauda*	EA44 T20131110.28HPZ2-01.MORRUF.01, Australia	Helix	Qiagen	N/A	N/A	PQ390894
67	*Lerista elegans*	EA45 R165983, Australia	Helix	Qiagen	N/A	N/A	PQ390895
68	*Lerista elegans*	EA46 R165978, Australia	Helix	Qiagen	N/A	N/A	PQ390896
69	*Morethia lineoocellata*	EA47 R165977, Australia	Helix	Qiagen	N/A	N/A	PQ390897
70	*Morethia lineoocellata*	EA48 R123950, Australia	Helix	Qiagen	N/A	N/A	PQ390898
71	*Ctenotus hanloni*	EA49 R157543, Australia	Helix	Qiagen	N/A	N/A	PQ390899
72	*Ctenotus hanloni*	EA50 R157557, Australia	Helix	Qiagen	N/A	N/A	PQ390900
73	*Ctenotus serventyi*	EA51 R102812, Australia	Helix	Qiagen	N/A	N/A	PQ390901
74	*Gehyra* sp.	EA55 R165742, Australia	Helix	Qiagen	N/A	N/A	PQ390902
75	*Gehyra* sp.	EA58 R163105, Australia	Helix	Qiagen	N/A	N/A	PQ390903
76	*Gehyra* sp.	EA59 R163044, Australia	Helix	Qiagen	N/A	N/A	PQ390904
77	*Gehyra* sp.	EA60 R165748, Australia	Helix	Qiagen	N/A	N/A	PQ390905
78	*Gehyra* sp.	EA61 R165749, Australia	Helix	Qiagen	N/A	N/A	PQ390906
79	*Gehyra* sp.	EA63 R165255, Australia	Helix	Qiagen	N/A	N/A	PQ390907
80	*Strophurus jeanae*	FD01 T20131203.29GPZ1-06.STRJEA.01, Australia	Helix	Qiagen	N/A	N/A	PQ390908

2.2 Optimisation of DNA Extraction Methods

2.2.1 Laboratory DNA extractions

A destructive DNA extraction method (i.e., involving complete sample homogenisation) was used on AHG samples to obtain "high-quality" DNA with a high yield, using multiple Qiagen (Germany) DNA extraction kits. DNA was extracted from AHG tissue samples (R109230 and R109520, Table 1) using the DNeasy Blood and Tissue extraction kit (Qiagen) to obtain high yield DNA for downstream application to conduct the AHG LAMP sensitivity test (see Resultsbelow).

 LInitial laboratory DNA extractions from scat samples were optimised using a two kits from Qiagen, QIAmp® PowerFecal® DNA kit and QIAmp® Fast DNA Stool Mini kit following the manufacturers' protocols.

Depending on the size of each scat a half or one-third of the scat was used in the DNA extraction with the remaining fraction other half stored for later use at -20 ^{o}C. A panel of DNA was destructively extracted from twenty AHG scat samples (collected from Dampier, Western Australia), using the QIAmp® Fast DNA Stool Mini extraction kit, which was found to produce DNA with a reasonably high yield. Two of these AHG scat samples were processed for downstream application to conduct the AHG LAMP sensitivity testing.

2.2.2 In-field compatible DNA extractions

Optimising an in-field compatible DNA extraction procedure is a pre-requisite for conducting the LAMP assay in the field. Non-destructive in-field DNA (i.e., not involving complete sample homogenisation) was extracted from three scat samples (collected from Dampier, WA) using commercial QuickExtract^{TM} DNA extraction (QE) solution 1.0 (Epicentre, USA). This method is rapid and easy to use, providing good quality DNA for use as a template in the LAMP assay. One hundred microliters of QE solution were pipetted into 6-wells of each Genie strip (OptiGene, UK). Depending on the size of the scat only one-third or half of it was transferred into each individual well and broken using a toothpick (single use to prevent cross contamination) [11], with up to six samples processed simultaneously in the Genie III (OptiGene, UK). The in-field QE DNA extraction protocol used the Genie III as an incubator programmed at 65 °C for 6 min followed by 98 °C for 2 min (total ~~run time~~ = 8 min), following published protocols [11]. To experimentally test the effect of reducing possible inhibitors, two microliters of undiluted QE DNA extracted from scats (1:1) was diluted, 1:10, 1:20 and 1:50, in water for use as a template in the LAMP assay.

Additional, non-destructive in-field DNA samples were extracted from 18 AHG tissue (WAM) samples using fifty microliters of QE solution (Table 1). Tissue samples were removed from ethanol and air dried on paper towel for approximately one minute before being transferred into each well of the Genie strip using a toothpick (single use, to prevent cross contamination). Each sample was immersed in QE, with up to six samples processed simultaneously in the Genie III, as above. All DNA samples were stored at -20 °C for future use.

2.3 Assessment of mitochondrial ND2 DNA sequence variation

Lizard species identifications were confirmed through DNA barcoding of the mitochondrial NADH dehydrogenase subunit 2 (ND2) locus, following published PCR conditions [26~~8~~], with the PCR annealing temperature reduced to 56 ºC. Most AHG ND2 sequences generated in the current study were obtained using the new AHG LAMP primer "AHG_F3" (Table 2) in combination with the existing primer M1123R, 5'-GCTTAATTAAAGTGTYTGAGTTGC-3' [26~~8~~] (~450 bp amplicon), for *Hemidactylus frenatus*.~~;~~ ~~with~~ ND2 was amplified from other lizard species using a new 'general' primer "GeckoND2-F" designed to be applicable to this suite of small lizard species 5'-MCAAACMCGAAAAATYATRGC-3', in combination with M1123R (~570 bp amplicon).~~,~~ ~~with~~ PCR's consisted~~ing~~ of 40 cycles with an annealing temperature of 53 ºC. PCR amplicons were sequenced commercially by Macrogen Inc. (Macrogen, Seoul, Korea) or Australian Genome Research Facility (AGRF) ~~AGRF (Australian Genome Research Facility)~~ in dual directions.

DNA sequence electropherograms were analysed using the software MEGA7 [27]. Forward and reverse sequences were paired, and a consensus sequence was generated. Genetic similarity of DNA sequences of AHG and non-target lizard species was examined using a maximum likelihood tree constructed in Mega7 [27~~32~~]. Pairwise sequence differences (p-distances) between species were generated in Mega7 [27] to assess percentage sequence divergences between species. Relationships among AHG samples were also examined through a haplotype network. DNA sequence variation in AHG specimens was examined through generation of a median joining haplotype network (ε = 0) [28~~9~~], constructed in PopArt [29~~30~~], using all new AHG sequences obtained here (Table 1, S1 Supplementary Table) and from sequences of specimens from around the world ~~worldwide sequences~~ obtained from GenBank [30~~1~~].

2.4 Development and evaluation of AHG LAMP assay

2.4.1 AHG dataset and primer design

AHG LAMP primers were developed for a section of the mitochondrial ND2 locus, that has been commonly used for DNA barcoding identification of geckos [26~~8~~, 30~~1~~]. This locus has been well characterised from multiple *Hemidactylus* species, including AHG which is known to ~~include~~ comprise multiple genetically distinct ND2 clades [30~~1~~]. Fifty-four ND2 DNA sequences for non-target gecko and skink species that co-occur in Western Australia (samples 27 to 80, Table 1) were generated (as in section 2.3)~~, as above~~. These were combined with thirty-one DNA sequences of AHG (same region of ND2) downloaded from GenBank, mostly derived from [30~~1~~]. The combined ND2 alignment was used to design eight novel LAMP primer regions ~~and~~ for six primers to target AHG (Table 2)~~.~~, including two inner primers (FIP and BIP, each comprised of two primer regions, F1+F2 and B1+B2 respectively), two outer primers (F3 and B3), and two loop primers Floop and Bloop. For all primers the GC content (%), predicted melting temperature (Tm), and potential secondary structure (hairpins or dimers) were analysed using the Integrated DNA Technologies (IDT) online OligoAnalyzer Tool ~~[33~~[31] using the qPCR parameter sets.

2.4.2 LAMP primer ratio optimisation

The primer ratio (F3/B3: FIP/BIP: Floop/Bloop) for this assay was optimised following published protocols using multiple ratios of primers ~~[17]~~[23]. Optimised primer master mix was prepared by adding the specified amount of each of the six primers in a 1:6:3 ratio. A 100 µL volume of primer master mix 1:6:3 (F3/B3: FIP/BIP: Floop/Bloop) was prepared by

adding 10 µL (10 µM) each of F3/B3, 6 µL (100 µM) each of FIP/BIP, 3 µL (100 µM) each of Floop/Bloop and 62 µL of Ultrapure water (Invitrogen, Australia).

2.4.3 LAMP assay conditions

The AHG LAMP assay was performed following published protocols ~~[15]~~[14]. Each LAMP reaction was conducted in a total volume of 25 µL containing 14 µL of Isothermal Master Mix (Iso-001, OptiGene, UK), 10 µL of the primer master mix (as in section 2.4.2) ~~mentioned above~~ and 1 µL of template DNA. A one microliter disposable plastic loop was used to add in-field DNA extract, ensuring that a fresh loop was used for each sample. The use of disposable plastic loops has been shown to be an effective method for transferring template DNA in LAMP reactions ~~[17]~~[23]. These loops reduce the risk of cross contamination and eliminate the need for pipettes when LAMP is used outside a laboratory, a necessity for effective field deployment.

Table 2. AHG LAMP primer and amplicon sequences (gBlock) and parameters. The F2 and B2 primer regions of FIP and BIP are bold and underlined. Lowercase letters in the gBlock indicate extra “ccc” added between LAMP primer sites to increase the overall Tm of the amplicon.

**Primers**	**Sequence 5'-3'**	**Primer length (bp)**	**Predicted Tm, annealing temperature (°C)**	**Degeneracy of primer (fold)**
AHG_F3	CRCCACAGCCATATTCAYA	19	55.9	4
AHG_B3	GGTAAGCTTGCGGCRAG	17	59.0	2
AHG_FIP	CCAAGTGACATAAGGGTAAYTATTA**<u>CACCACAACCAARACCATTC</u>**	45	76.4	4
AHG_BIP	CTAACAGGRTTYATGCCAAAAT**<u>CGCYGATKGGYAGGAG</u>**	38	73.5	32
AHG_Floop	GGGTTAGGGCTGGTGATAKTA	21	66.9	2
AHG_Bloop	KACTTATCATTAAAGACCTYGC	22	46.1	4
gBlock Fragment	cccCACCACAGCCATATTCACACACCACAACCAAGACCATTCcccTACT ATCACCAGCCCTAACCCcccTAATAATTACCCTTATGTCACTTGGcccCT AACAGGGTTTATGCCAAAATcccGACTTATCATTAAAGACCTTGCcccC TCCTACCAATCGGCGcccCTCGCCGCAAGCTTACCccc	186	N/A	N/A

High-quality DNA (Qiagen) extracted from AHG tissue samples was used as a positive control for optimising the LAMP assay. A no template control (NTC) was included in each test to detect reagent contamination. All the LAMP assays were run in the Genie III at 65 °C for 25 min followed by an annealing curve analysis from 98 °C to 73 °C with ramping at 0.05 °C/s. The total run time ~~being~~ was approximately 35 minutes. The amplification and anneal derivative temperature ~~curves~~ profiles (anneal derivative) were visualised on the Genie III screen to ensure that amplification occurred as expected. Positive results were further confirmed through the annealing step resulting in a single product peak at a specific temperature, while the negative (NTC) and non-target lizard DNA remained as a flat line. Results were analysed using a PC version of the software Genie Explorer version 2.0.7.11 in the blue channel.

2.4.4 Evaluation of a gBlock gene fragment for AHG LAMP assay

Gene Fragment (gBlocks) are targeted synthetic oligonucleotides originally developed to be used as standards in real-time PCR reactions [32] but are equally applicable to LAMP assays as controls to monitor assay performance as synthetic positive controls [14]. To develop a consistent positive control for use in the LAMP assay, a 186 bp gBlock gene fragment (Integrated DNA Technologies, Iowa, USA), modified from the complete fragment used for the assay, was designed for use as a synthetic DNA positive control. This synthetic fragment consisted solely of concatenated LAMP primers separated by runs of "ccc", to increase the overall Tm of the fragment (Table 2). To evaluate detection sensitivity, the copy number was determined, ~~copy number~~ and a ten-fold serial dilution (1:10) of the gBlock was prepared as outlined in [11]. Sensitivity of the serially diluted gBlock was tested in the Genie III following the same AHG LAMP assay amplification conditions ~~as mentioned above~~(as in section 2.4.3) with the ~~run~~ amplification time increased from 25 minutes to 35 minutes, with

Formatted: Don't adjust space between Latin and Asian text, Don't adjust space between Asian text and numbers

this longer amplification time included to allow detection of the gBlock present at very low concentrations. Following this, another LAMP run was conducted to determine the best dilution to be used as a positive control in the LAMP assay. The same five-fold serial dilution (10 ng/µL to 3.2 x 10^{-3} ng/µL) of high-quality AHG tissue DNA (R109520, used previously) with up to six dilutions was used as a template to compare amplification time with a standard amount of one million copies (1 x 10^{5} copies/µL) of gBlock.

2.5 Analytic sensitivity of the AHG LAMP assay

To test for the sensitivity of the LAMP assay a five-fold serial dilution of high-quality DNA extract of two biological replicates of AHG scat samples (collected from Dampier, Western Australia) and two biological replicates of AHG tissue samples (R109230 and R109520, Table 1) were prepared using Ultrapure water (Invitrogen, Life Technologies, Australia). Starting DNA concentrations of all four samples were quantified using a Qubit 2.0 Fluorometer (Invitrogen, Life Technologies, Australia) following the manufacturers protocol. All four AHG DNA samples were serially diluted (total eight dilutions) from 10 ng/µL to 1.28 x 10^{-4} ng/µL (1:1 to 1:78125). Sensitivity of the LAMP assay was tested using the serially diluted DNA in the Genie III following the same AHG LAMP assay amplification conditions (as in section 2.4.3), as above. The time of amplification and anneal derivative temperature were recorded for all 8 dilutions. To further explore any differences between DNA obtained from AHG tissue compared with DNA obtained from scats LAMP amplification times and anneal derivative temperatures were compared using Student's T-tests in Excel (365 MSO, Version 2503).

2.67 Specimens examined for field testing and lab validation of field samples

In total, 108 samples were tested for the AHG LAMP assay in the field. These samples consisted of 100 scats (S1 Supplementary Table) collected from multiple Western Australian field locations: Barrow Island (n = 49), Dampier (n = 17), Karratha (n = 33) and Perth (n = 1); as well as eight tissue samples collected from Karratha, Australia (n = 4) and Batam, Indonesia (n = 4). DNA was extracted from both scats and tissue samples using the in-field QE DNA extraction method using the Genie III. The AHG LAMP assay was performed (as in section 2.4.3)~~as outlined above~~. Amplification time and anneal derivative temperature of all scats, tissues, gBlock (1 x 10^6 copies/µL) synthetic positive control, and negative no template control, were recorded.

All 108 non-destructive QE DNA extracts, and 100 vials containing the other two-thirds / half of the field collected lizard scats were shipped to the laboratory on dry ice. These were then processed for AHG LAMP assay in the laboratory to validate AHG positive samples. Note that 1 µL loops were used to transfer template into master-mix for field testing whereas 1 µL pipette tips were used for adding template in the laboratory. High-quality DNA was extracted from the 100 dry lizard scat samples using the QIAamp® Fast DNA Stool Mini kit (Qiagen, Australia). These DNA extracts were used for further confirmation for species identification through ND2 DNA barcoding (S1 Supplementary Table) using methods~~the method~~ outlined above (~~as~~ section 2.3~~described above~~), ~~using~~ and primers GeckoND2-F / M1123R.

3. RESULTS

3.1 AHG LAMP assay design and optimisation

Novel LAMP primers were developed to target a 231 bp amplicon for AHG for the mitochondrial ND2 locus (Table 2, Figure 1). ~~The AHG LAMP assay consists of six primers (Table 2), two inner primers (FIP and BIP), two outer primers (F3 and B3) and two loop primers Floop and Bloop (Table 2, Figure 1).~~ Assessment of the primer ratio (F3/B3: FIP/BIP: Floop/Bloop) for this assay found the optimal ratio to be 1:6:3, with final concentrations of 0.4 µM, 2.4 µM and 1.2 µM for the F3/B3, FIP/BIP and Floop/Bloop primers respectively.

Figure 1. AHG LAMP primer alignment. Arrows indicate primer direction; Loop primers are underlined; FIP (5'-3') is made by combining F1 (reverse compliment) and F2; BIP (5'-3') is made by combining B1 (reverse compliment) and B2. Grey-shading indicates AHG, the target species, while non-target gecko species are unshaded. AHG DNA sequences were obtained from [30~~1~~], while non-target DNA sequences were newly generated. AHG group letters (A-D) are from [30~~1~~].

3.2 DNA barcoding of specimens tested in the LAMP assay

Species-level identifications of lizard samples examined in the current study were confirmed through DNA barcoding of the ND2 locus (Table~~s 2 and~~ 3, Figure 2, S1 Supplementary Table). Relationships among AHG samples were also examined using a haplotype network (Figure 3), which indicated that most Australian AHG samples were very closely related, with a small number of Australian and intercepted specimens being more genetically divergent. Interestingly, some of the Cocos Island (Australian territory) samples matched the most common Australian haplotype found, while others belonged to a highly divergent lineage (>6% different), that had not previously been genetically characterised ("Group E" on

Figure 3). ~~The presence of these two highly divergent genetic lineages indicates that there have likely been at least two introduction events to the Cocos Islands.~~

Figure 2. Maximum-likelihood tree of relationships between *Hemidactylus frenatus* (AHG, in bold, GenBank reference sequences) with lizard species collected from Western Australia.

Figure 3. Haplotype network of all AHG sequences obtained from GenBank and generated in the current study. Letters refer to previously recognised genetic groups of AHG (following clade naming system in [30~~1~~]), apart from 'E' (Cocos Island, territory of Australia), which is newly reported here. Australian samples examined in the current study are shown in green. Red samples were intercepted in transit to Barrow Island, Australia.

3.3 DNA extractions

High-quality DNA extracted using the QIAamp® Fast DNA Stool Mini kit yielded enough DNA from AHG scats for downstream application, while t~~. T~~he non-destructive DNA extraction method from scats and tissues using QuickExtract™ was found to be suitable for conducting LAMP assays in the field. All in-field QE DNA extracts from tissue samples, n=18 (Table 1) amplified within the expected time range 10 ± 2.8 minutes (average ± SD). The anneal derivative temperature for all the samples was 85.3 ± 0.2 ^{o}C.

Additionally, three in-field QE DNA extracts from scat samples were tested both undiluted (1:1) and diluted at 1:10, 1:20 and 1:50 (Figure 4 & S2 Supplementary Data). All four DNA dilutions amplified within the expected time range; with 1:1 in 11.75 ± 1.75 minutes, 1:10 in 12.3 ± 1.9 minutes, 1:20 in 16 ± 5.2 minutes and 1:50 in 11.9 ± 0.5 minutes. From these

results we recommend using DNA at 1:1, because~~as~~ amplification times were not substantially shorter following dilution. All the samples tested produced an anneal derivative ~~curve~~ temperature at ~85.5 °C, which was not affected by DNA dilution.

Figure 4. Optimal method for obtaining in-field DNA extracts in the field. **a)** Scats, 1/3 approx. 3 x 3 mm (left), 1/2 approx. 3 x 5 mm (right). **b)** The same scats in 1.5 ml tubes, pre-DNA extraction. **c)** Extracted DNA (100 µL QuickExtract™) (1:1) subsequently diluted (1:10, 1:20 and 1:50) using water.

3.4 Performance of the LAMP assay

T~~Overall, t~~he AHG LAMP assay produced amplification from AHG DNA from tissue samples in an average of 10.0 ± 2.7 minutes (Figure 5a and 5c, Table 1), with an anneal derivative ~~profile~~ temperature at 85.4 ± 0.2 °C (Figure 5b and 5d, Table 1); with amplification in under 25 minutes considered as positive. The non-template control in all the LAMP runs tested did not amplify confirming the absence of primer dimer, ~~confirming~~ and that there were no false positive amplifications due to primer interactions or reagent contamination (Figure 5a and 5c).

Figure 5. Amplification and anneal derivative temperature profiles for the optimised AHG LAMP assay using 1:6:3 primer ratio. **a)** Positive amplification profile of seven AHG tissue samples within 6 to 13 minutes (in-field QuickExtract DNA extracts). Negative, no amplification (flat line, blue). **b)** Anneal derivative temperature is ~85.4 °C for all 7 AHG tissue DNA. **c)** Positive amplification profile of 7 AHG scat samples within 10 to 14 minutes (high-quality Qiagen DNA extracts). Negative, no amplification (flat line, blue). **d)** Anneal

derivative temperature for scat and tissue samples are the same ~~as~~ at 85.4 ^{o}C. **e)** Amplification profile of five-fold dilution of AHG tissue DNA (R109520) up to 6 dilutions ranging from 10 ng/µL to 3.2 x 10^{-3} ng/µL (amplification time from highest to lowest DNA concentration was 8 to 12 minutes) and gBlock 1 x 10^{5} copies/µL, at 10 minutes (pink). Negative, no amplification (blue). **f)** Anneal derivative profiles of LAMP amplicons with similar peaks, 85.4 ^{o}C for AHG DNA and 85.5 ^{o}C for gBlock (pink).

"Blind" sample LAMP testing of the 108 tissue and scat samples was conducted in-field producing positive amplification only from AHG tissue and scats (n=38, S1 Supplementary Table). Subsequent laboratory testing showed eight additional AHG scats (21% of total scats identified as AHG) were not detected through the initial in-field DNA extractions ~~through~~ by LAMP, with these samples requiring laboratory high-quality DNA extractions to produce enough DNA for secondary LAMP testing or DNA sequencing and barcoding identification (Table 3, S1 Supplementary Table). Generally, 45% of the field collected lizard scats could not be amplified in the laboratory by PCR for DNA barcoding. Importantly, none of the non-target lizard scats (five other species) amplified in the field or laboratory, including all forty-nine scat samples tested from Barrow Island (S1 Supplementary Table).

Table 3. Field validation of AHG LAMP assay on lizard scat samples. AHG samples indicated by grey shading. Species identifications were made through positive LAMP amplification and/or subsequent laboratory DNA barcoding of ND2 (see S1 Supplementary Table).

**Species**	**Collected Mainland, Australia**	**Collected Barrow Island, Australia**	**LAMP Positive (in field)**	**LAMP Negative (in field)**
Hemidactlyus frenatus	38	0	30	8
Cryptoblepharus plagiocephalus	0	3	0	3
Gehyra pilbara	1	11	0	12
Gehyra sp.	1	0	0	1
Gehyra variegata	0	5	0	5
Heteronotia binoei	1	0	0	1

3.5 Evaluation of AHG gBlock gene fragment

The detection sensitivity of AHG gBlock gene fragment evaluated for templates ranging from ~100 million copies down to ~10 copies was quite ~~sensitive~~high, ~~detecting~~ with as low as 1 x 10 copies/µL detected within 15 minutes. From the amplification profile ~~one hundred thousand~~100,000 copies (1 x 10^5 copies/µL) of gBlock amplified in 10 minutes which equates to ~2 ng/µL of AHG DNA (Figure 5e). Based on the sensitivity test of AHG gBlock 1 x 10^6 copies/µL of gBlock amplified in 8 minutes. The ~~amplification time of~~ neat DNA concentration, prior to dilution ~~starting DNA amount of the dilution series (was~~ 10 ng/µL), ~~of DNA which~~ amplified in 8 minutes (Figure 5e). Hence the recommended gBlock dilution would be 1 x 10^6 copies/µL for use as synthetic positive control for future LAMP runs. The

anneal derivative profiles of LAMP amplicons show two very similar peaks, 85.4 °C for AHG DNA and 85.5 °C for gBlock (Figure 5f).

The gBlock 1 x 10^6 copies/µL was used in all the LAMP runs as a synthetic positive control to monitor the performance of the ~~LAMP~~ assay in the field. ~~In~~ Across eighteen LAMP runs the time for positive amplification was consistent, amplifying in 12 ± 0.6 minutes with an anneal derivative temperature of 85 ± 0.3 °C. Furthermore, the negative control which had no DNA template (NTC) did not amplify in any of the ~~in all~~ eighteen LAMP runs conducted, ~~did not amplify~~ showing a flat line both in the amplification and anneal derivative profile ~~curve~~s confirming the absence of contaminants in the reagent mix. The results of AHG LAMP assay from both the field testing and laboratory assays showed similar results, demonstrating ~~proving~~ the robustness of the assay.

3.6 Sensitivity of LAMP assay

The sensitivity of the AHG LAMP assay was tested on five-fold serial dilutions of two biological replicates of each AHG tissue and scat DNA. The time to amplify DNA increased as DNA template concentrations reduced (Figure 6). All eight DNA dilutions from tissue samples produced positive amplification curves with the highest DNA concentration (10 ng/µL) amplifying in 8 minutes compared with the scat DNA which amplified in 11 ± 1.4 minutes (Figure 6). Th~~eis~~ LAMP test was sensitive in detecting DNA from tissue samples down to the lowest level 1.28 x 10^{-4} ng/µL (0.28 picogram) of DNA in 22 minutes. Sensitivity was lower for DNA dilutions of scat samples ~~were less sensitive~~, with amplification ~~only amplifying~~ up to only four dilutions (8 x 10^{-2} ng/µL) in 14 minutes (Figure 6). T-tests (S2 Supplementary Data) showed a significant ($p < 0.02$) difference between

amplification times for DNA derived from tissue and from scats, while there was no significant difference observed for their anneal derivative profiles~~s~~ (p >0.25).

Figure 6. AHG LAMP sensitivity test for a five-fold serial dilution of DNA samples from two AHG tissue (R109230 and R109520) (circles) and two AHG scat (from Dampier, Western Australia) (triangles). Amplification times are shown for all 4 DNA samples with DNA concentration ranging from highest (10 ng/µL) to lowest (1.28 x 10^{-4} ng/µL). All eight DNA dilutions of tissue samples amplified (10 ng/µL DNA amplified in 8 minutes and 1.28 x 10^{-4} ng/µL in 22 minutes). DNA dilutions of scat samples only amplified up to four dilutions (10 ng/µl DNA amplified in 10 and 12 minutes and 8 x 10^{-2} ng/µL in 14 minutes).

4. DISCUSSION

This study designed and optimised a new LAMP assay for the detection of an invasive vertebrate species. The assay was successfully applied to both tissue and scat AHG samples (Tables 1 and 3). These samples represented much of the known AHG genetic diversity (compared with ND2 sequences from [30~~1~~]), with some specimens from AHG “Group E” being more than 6% genetically divergent from other groups (Figure 3). AHG is known to possess highly distinct genetic lineages, ~~which~~ and likely represents a species complex [30~~1~~, 33~~4~~]. ~~Interestingly, the~~The presence of ~~these~~ two highly divergent genetic lineages amongst our samples indicates that there have likely been at least two independent introduction events to the Cocos (Keeling) Islands.

~~Notably, However, the~~T~~t~~he new LAMP assay performed well on all samples tested including those from “Group E”, allowing all positive AHG samples to be rapidly identified, apart from a small number of field-collected scat samples which yielded ~~possessed~~ low amounts of DNA. ~~The presence of these two highly divergent genetic lineages indicates that there have likely been at least two introduction events to the Cocos Islands.~~Unfortunately, we did not have access to specimens of any congeneric species to experimentally test the performance of the AHG LAMP assay on other *Hemidactylus* species. Another closely related species, *Hemidactylus platyurus,* occurs north of Australia, however this species (represented by GenBank EU268352) is 36% different for the section of ND2 used in the LAMP assay, so would ~~be~~ unlikely ~~to~~ amplify.

We designed and optimised a synthetic DNA positive control (gBlock) for use in the AHG LAMP assays following [11]. This synthetic DNA is beneficial in: (i) providing a consistent control to allow tracking of the performance of LAMP assays across runs, and (ii) providing a relatively high amount of control DNA compared with DNA extractions from gecko scat specimens. Scat samples in general produce very low DNA yields [1] due to the small amount of DNA contained within scats. DNA is present at low levels in scat samples compared to blood or tissue samples ~~[3~~[34~~5~~], ~~with scat DNA~~and can be further reduced by unfavourable environmental conditions~~,~~ with DNA degrading over time ~~[3~~[35~~6~~]. Therefore, the freshest samples should be used when possible. If the scats are not assayed immediately, they can be preserved frozen (approx. -20 °C) to reduce the ~~speed~~ rate of DNA degradation. For field collected scat samples, the LAMP assay reduces the risk associated with storage of scats, by providing an in-situ assay that can be completed immediately as required.

When working with scats, the risk of cross contamination is increased due to the low amount of target DNA contained within scats ~~[3~~[34~~5~~]. To reduce this risk, additional precautions should be taken when field collecting scats. The items used to collect samples should be disposable or washed and sterilised between each sample to avoid cross-contamination. Similarly, the amplicons produced during LAMP assays could result in potential cross contamination between samples if precautions, such as not opening LAMP reaction tubes post amplification, are not followed. For this reason, disposable items should also be used to avoid transferring amplified DNA into the pre-amplification working space, and negative no-template controls should always be included to test for possible LAMP reagent contamination ~~[17]~~[23].

Amplification of the LAMP assay was found to become slower in a predictable manner as DNA template concentrations reduced. At low DNA concentration of AHG tissue <0.001 ng/μ~~u~~l, the average LAMP amplification time was less than 15 minutes (Figure 6). Similarly, the average LAMP amplification time of gecko scat DNA at low concentrations, <1 ng/μ~~u~~l was also less than 15 minutes (Figure 6). The difference between the lower amplification threshold of tissue and scat samples is likely due to the presence of non-target DNA (e.g., bacteria, digested food etc.) in the scat samples overinflating the overall DNA quantification measurements, with scats also known to have degraded DNA and amplification inhibitors [1, 35].

The ~~size~~ amount of the scat used in DNA extraction also appears to influence the quality and quantity of DNA extracted. If whole scat size were used (rather than the recommended one half or third), amplification times were found to increase, likely due to amplification

inhibitors such as humic acid present in the scats, which is known to slow LAMP reactions [3[367]. Other molecular diagnostic approaches, such as DNA barcoding and real-time PCR, are known to be even more sensitive to the presence of inhibitors, and are unlikely to be able to amplify from crude (scat) DNA extracts [1, 36]. This, combined with the ease of use, and ability to rapidly amplify and detect positive targets in portable laboratory / in-field compatible devices are the major advantages LAMP has over other molecular approaches [36].

The LAMP assay outlined here provides a reliable screening tool for the detection of AHG in the field, detecting 100% of AHG tissue samples, and 79% of field scat samples from traces (scats) of AHG. However, as when using any molecular approach, a positive AHG detection should always be verified, particularly if the detection is in an unusual or previously undocumented location. Verification can be performed through using standard DNA barcoding methods, as conducted here. Our study provides new ND2 DNA barcode sequences for an additional twenty-five gecko and skink species present in Western Australia, which greatly improves resources available for molecular identification of these small lizards in Australia. However, verification through DNA barcoding is relatively slow, requiring a turnaround time of days to weeks, compared with LAMP which can produces results in approximately one hour. Therefore, it may be preferable to verify positive LAMP results by increased sampling efforts, through LAMP confirmation of additional samples from the area the detection was made.

The AHG LAMP assay was operationalised in 2022 and is now widely used by Chevron Australia in their biosecurity operations in Australia. This new tool provides accurate rapid

diagnosis of intercepted AHG (e.g., Table 1). In August 2023, a scat was collected that was suspected of originating from an AHG, detected on a half-height container during a quarantine inspection at the border of Barrow Island. Following in-situ analysis of the scat using the new AHG LAMP assay the scat was identified as originating from an AHG. A rapid decision was made to reject the cargo and vessel from Barrow Island. Rejecting cargo and vessels from any port is an expensive operation that is usually met with resistance from stakeholders. The AHG LAMP assay has reduced the subjectivity associated with this decision, which ultimately enhanced cooperation from stakeholders, leading to a quick response. The scat was subsequently sent to Perth for validation of identification through DNA barcoding (~~as above~~as in section 2.3) with DNA sequencing results confirming the LAMP assay identification of an AHG with 100% confidence.

ACKNOWLEDGEMENTS

Scat samples from Barrow Island were sourced by Murdoch University under animal ethics permit number R2859/16. Scats samples from Dampier were provided by Chevron Australia. Tissue samples were provided by both the Western Australian Museum and Helix Molecular Solutions. These tissue samples were obtained from preserved specimens from the WAM, or through a Cadaver and Tissue Usage Notification for~~Tissue samples were provided by both the Western Australian Museum~~m~~ and Helix Molecular Solutions, under a Cadaver and Tissue Usage Notification form, and/or the above permit~~.

S1 Supplementary Table. Samples tested in-field: n=100 scats, n=8 tissue samples. Species identify determined through positive LAMP result and/or DNA barcoding.

S2 Supplementary Data. T-test comparisons of LAMP amplification times and anneal derivatives from scat and tissue DNA samples. LAMP optimisation runs for proportion of scat sample extracted, and scat DNA template dilutions in water (1:1, 1:10, 1:50).

Data Availability Statement: All relevant data are within the paper and supporting information files (S1 Supplementary Table & S2 Supplementary Data). ND2 DNA sequences from this study have been accessioned in GenBank (PQ390829 - PQ390908).

Funding: This study was supported through Murdoch University grant MU-112020, and by the State Government of Victoria (Agriculture Victoria Research). This study was funded by the Gorgon Project. The Gorgon Project is operated by an Australian subsidiary of Chevron and is a joint venture of the Australian subsidiaries of Chevron (47.3%), ExxonMobil (25%), Shell (25%), Osaka Gas (1.25%), MidOcean Energy (1%), and JERA (0.417%). The funders had no role in study design, data collection and analysis, decision to publish, or preparation of the manuscript.

Competing interests: The authors declare that no competing interests exist.

AUTHOR CONTRIBUTIONS

Conceptualization: Melissa Thomas.

Data curation: Arati Agarwal, Mark Blacket.

Formal analysis: Arati Agarwal, Mark Blacket.

Funding acquisition: Brendan Rodoni, Mark J. Blacket, Simon McKirdy, Melissa Thomas.

Investigation: Arati Agarwal, Mark Blacket, Yvette Hitchen

Methodology: Arati Agarwal, Mark Blacket, Yvette Hitchen

Resources: Yvette Hitchen, Paul Doughty

Supervision: Arati Agarwal, Melissa Thomas, Yvette Hitchen, Mark Blacket.

Writing – original draft: Arati Agarwal, Melissa Thomas, Mark Blacket.

Writing – review & editing: Arati Agarwal, Melissa Thomas, Yvette Hitchen, Paul Doughty, Simon McKirdy, Brendan Rodoni, Mark Blacket.

REFERENCES

[1] Waits LP, & Paetkau D. Noninvasive genetic sampling tools for wildlife biologists: A review of applications and recommendations for accurate data collection. Journal of Wildlife Management. 2005; 69, 1419-1433.

[2] Croose E, Birks JDS, O'Reilly C, Turner P, Martin J, & Macleod ET. Sample diversity adds value to non-invasive genetic assessment of a pine marten (*Martes martes*) population in Galloway Forest, southwest Scotland. Mammal Research. 2016; 61, 131-139.

[3] Rajpoot A, Kumar VP, Bahuguna A, & Rasaily SS. Preliminary genetic documentation of snake species through shed skin from Uttarakhand, India: A non-invasive genetic sampling approach. Journal of Wildlife and Biodiversity. 2021; 5; 81-91.

[4] Berry O, Sarre SD, Farrington L, & Aitken N. Faecal DNA detection of invasive species: the case of feral foxes in Tasmania. Wildlife Research. 2007; 34, 1-7.

[5] Waits LP. Using noninvasive genetic sampling to detect and estimate abundance of rare wildlife species. In: THOMAS, W. L. (ed.) Sampling rare or elusive species: concepts, designs, and techniques for estimating population parameters. Washington, D. C., U.S.A.: Island Press 2004.

[6] Piggott MP, & Taylor AC. Remote collection of animal DNA and its applications in conservation management and understanding the population biology of rare and cryptic species. Wildlife Research. 2003; 30, 1-13.

[7] Pennell MW, Stansbury CR, Waits LP, & Miller CR. Capwire: a R package for estimating population census size from non-invasive genetic sampling. Molecular Ecology Resources. 2013; 13, 154-157.

[8] Baumgardt JA, Goldberg CS, Reese KP, Connelly JW, Musil DD, Garton EO, & Waits LP. A method for estimating population sex ratio for sage-grouse using noninvasive genetic samples. Molecular Ecology Resources. 2013; 13, 393-402.

[9] Idaghdour Y, Broderick D, Korrida A. Faeces as a source of DNA for molecular studies in a threatened population of great bustards. Conservation Genetics. 2003; 4:789–792.

[10] Notomi T, Okayama H, Masubuchi H, Yonekawa T, Watanabe K, Amino N, & Hase T. Loop-mediated isothermal amplification of DNA. Nucleic Acids Research. 2000; 28, e63.

[11] Agarwal A, Cunningham JP, Valenzuela I, & Blacket MJ, A diagnostic LAMP assay for the destructive grapevine insect pest, phylloxera (*Daktulosphaira vitifoliae*). Scientific Reports. 2020; 10:1-10.

[12] Rako L, Agarwal A, & Blacket MJ. A LAMP (Loop-mediated isothermal amplification) test for rapid identification of Khapra beetle (*Trogoderma granarium*), Pest Management Science. 2021; 77: 5509-5521.

[13] Rako L, Agarwal A, Eow L, Roberts JMK, Rodoni BC & Blacket MJ. LAMP (Loop-mediated isothermal amplification) assay for rapid identification of Varroa mites, Scientific Reports. 2023; 13: 11931.

~~[14] Conte J, Potoczniak MJ, Tobe SS, Using synthetic oligonucleotides as standards in probe-based qPCR. BioTechniques. 2018; 64: 177-179.~~

[14~~5~~] Agarwal A, Rako L, Schutze MK, Starkie ML, Tay WT, Rodoni BC, & Blacket MJ. A diagnostic LAMP assay for rapid identification of an invasive plant pest, fall armyworm *Spodoptera frugiperda* (Lepidoptera: Noctuidae). Scientific Reports. 2022; 12(1): 1116.

[16] Doherty TS, Glen AS, Nimmo DG, Ritchie EG, & Dickman CR. Invasive predators and global biodiversity loss. Proceedings of the National Academy of Sciences. 2016; 113, 11261-11265.

[15] Hoskin CJ, The invasion and potential impact of the Asian House Gecko (*Hemidactylus frenatus*) in Australia. Austral Ecology. 2011; 36, 240-251.

[16] Hopkins JM, Higgie M, & Hoskin CJ. Calling behaviour in the invasive Asian house gecko (*Hemidactylus frenatus*) and implications for early detection. Wildlife Research. 2020; 48, 152-162.

[17] Cole NC, Jones CG, & Harris S. The need for enemy-free space: The impact of an invasive gecko on island endemics. Biological Conservation. 2005; 125, 467-474.

[18] Barnett LK, Phillips BL, & Hoskin CJ. Going feral: Time and propagule pressure determine range expansion of Asian house geckos into natural environments. Austral Ecology. 2017; 42, 165-175.

[19] Barnett LK, Phillips BL, Heath ACG, Coates A, & Hoskin CJ. The impact of parasites during range expansion of an invasive gecko. Parasitology. 2018; 145, 1400-1409.

[20] Chapple DG, Knegtmans J, Kikillus H, & Van Winkel D. Biosecurity of exotic reptiles and amphibians in New Zealand: building upon Tony Whitaker's legacy. Journal of the Royal Society of New Zealand. 2016; 46, 66-84.

[21] Scott JK, McKirdy SJ, Van der Merwe J, Green R, Burbidge AA, Pickles G, Hardie DC, Morris K, Kendrick PG, Thomas ML, Horton KL, O'Connor SM, Downs J, Stoklosa R, Lagdon R, Marks B, Nairn M, & Mengersen K. Zero-tolerance biosecurity protects high-conservation-value island nature reserve. Scientific Reports. 2017; 7: 772.

[22] Van der Merwe J. Barrow Island quarantine: beyond best practice. Abu Dhabi International Petroleum Exhibition and Conference. Abu Dhabi: Society of Petroleum Engineers. 2015.

[~~1~~23~~7~~] Blacket MJ, Agarwal A, Zheng L, Cunningham JP, Britton D, Schneider I, & Rodoni BC. A LAMP assay for detection of *Bactrocera tryoni* Queensland fruit fly (Diptera: Tephiritidae). Scientific Reports. 2020; 10: 9554.

[~~1~~24~~8~~] Starkie ML, Fowler EV, Zhu X, et al. Loop-mediated isothermal amplification (LAMP) assays for detection of the New Guinea fruit fly *Bactrocera trivialis* (Drew) (Diptera: Tephritidae). Scientific Reports. 2022; 12, 12602.

~~[14] Conte J, Potoczniak MJ, Tobe SS, Using synthetic oligonucleotides as standards in probe-based qPCR. BioTechniques. 2018; 64: 177-179.~~

[25~~19~~] Heers T, van Neer A, Becker A, Grilo ML, Siebert U, Abdulmawjood A. Loop-mediated isothermal amplification (LAMP) assay - A rapid detection tool for identifying red fox (Vulpes vulpes) DNA in the carcasses of harbour porpoises (*Phocoena phocoena*). PLoS One. 2017; 12(9):e0184349.

~~[20] Hoskin CJ, The invasion and potential impact of the Asian House Gecko (*Hemidactylus frenatus*) in Australia. Austral Ecology. 2011; 36, 240-251.~~

~~[21] Hopkins JM, Higgie M, & Hoskin CJ. Calling behaviour in the invasive Asian house gecko (*Hemidactylus frenatus*) and implications for early detection. Wildlife Research. 2020; 48, 152-162.~~

~~[22] Cole NC, Jones CG, & Harris S. The need for enemy-free space: The impact of an invasive gecko on island endemics. Biological Conservation. 2005; 125, 467-474.~~

~~[23] Barnett LK, Phillips BL, & Hoskin CJ. Going feral: Time and propagule pressure determine range expansion of Asian house geckos into natural environments. Austral Ecology. 2017; 42, 165-175.~~

~~[24] Barnett LK, Phillips BL, Heath ACG, Coates A, & Hoskin CJ. The impact of parasites during range expansion of an invasive gecko. Parasitology. 2018; 145, 1400-1409.~~

~~[25] Chapple DG, Knegtmans J, Kikillus H, & Van Winkel D. Biosecurity of exotic reptiles and amphibians in New Zealand: building upon Tony Whitaker's legacy. Journal of the Royal Society of New Zealand. 2016; 46, 66-84.~~

~~[26] Scott JK, McKirdy SJ, Van der Merwe J, Green R, Burbidge AA, Pickles G, Hardie DC, Morris K, Kendrick PG, Thomas ML, Horton KL, O'Connor SM, Downs J, Stoklosa R, Lagdon R, Marks B, Nairn M, & Mengersen K. Zero-tolerance biosecurity protects high-conservation-value island nature reserve. Scientific Reports. 2017; 7: 772 .~~

~~[27] Van der Merwe J. Barrow Island quarantine: beyond best practice. Abu Dhabi International Petroleum Exhibition and Conference. Abu Dhabi: Society of Petroleum Engineers. 2015.~~

[~~28~~26] Sistrom MJ, Hutchinson MN, Hutchinson RG, & Donnellan SC. Molecular phylogeny of Australian *Gehyra* (Squamata: Gekkonidae) and taxonomic revision of *Gehyra variegata* in south-eastern Australia. Zootaxa. 2009; 2277, 14-32.

[27] Kumar S, Stecher G, and Tamura K. MEGA7: Molecular Evolutionary Genetic Analysis version 7.0 for bigger datasets. Molecular Biology and Evolution. 2016; 33: 1870-1874.

[~~29~~28] Bandelt H, Forster P, & R¨ohl A. Median-joining networks for inferring intraspecific phylogenies. Molecular Biology and Evolution. 1999; 16(1), 3748.

[~~30~~29] Leigh JW, & Bryant D. Popart: full-feature software for haplotype network construction. Methods in Ecology and Evolution. 2015; 6, 1110–1116.

[~~31~~30] Agarwal I, Jablonski D, & Bauer AM. The identity and probable origin of the *Hemidactylus* geckos of the Maldives. Herpetological Journal. 2019; 29: 230-236.

~~[32] Kumar S, Stecher G, and Tamura K. MEGA7: Molecular Evolutionary Genetic Analysis version 7.0 for bigger datasets. Molecular Biology and Evolution. 2016; 33: 1870-1874.~~

~~[33~~[31] OligoAnalyzer Tool. https://sg.idtdna.com/calc/analyzer

[~~1~~32]~~4]~~ Conte J, Potoczniak MJ, Tobe SS, Using synthetic oligonucleotides as standards in probe-based qPCR. BioTechniques. 2018; 64: 177-179.

[3~~4~~34] Rocha S, Trinks A, Harris DJ, Larson G and Cheke AS. The Global and Western Indian Ocean Dispersal of House Geckos From Asia Using Historical and Mitochondrial DNA Perspectives. Frontiers in Ecology and Evolution. 2022; 10, 791762.

[~~35~~34] Taberlet P, Waits LP, & Luikart G. Noninvasive genetic sampling: look before you leap. Trends in Ecology & Evolution. 1999; 14, 323-327.

[~~36~~35] Piggott MP. Effect of sample age and season of collection on the reliability of microsatellite genotyping of faecal DNA. Wildlife Research. 2004; 31, 485-493.

~~[3~~[36~~7~~] Nwee~~ew~~ MK, Jangpromma N, & Taemaitree L. Evaluation of molecular inhibitors of loop-mediated isothermal ~~amplifcation~~amplification (LAMP). 2024; 14, 5916.

Formatted: Not Highlight

Formatted: Not Highlight

Response To Reviewers - PONE-D-25-13902R2

Reviewer #2: The authors have addressed all the comments raised in the previous version of the manuscript. In the present state, the manuscript is acceptable for the publication.

Thank you.

Reviewer #3: This manuscript describes development of a novel tool for detection of an invasive species. The work is technically sound and worthy of publication. The manuscript is generally well written, but there are some corrections and clarifications needed to meet the standard for publication. I have provided comments and suggested changes on the attached PDF and summarise the recommendations here:

General comments

Check usage of commas, these are sometimes missing where appropriate to use and in other cases are included inappropriately. I have made suggested changes on the PDF but please check throughout

DONE

The term 'in-field' is sometimes spelled as "infield" which isn't the intended meaning - make sure "in-field" is used consistently

DONE

There are several sentences with repeated use of the same or very similar terms. While not strictly incorrect, these can be jarring to read, and I'd recommend rewording to avoid repetition where possible. I have commented on examples in the PDF

DONE

The word "as" is used a lot in the text, with various meanings. While its meaning is usually clear from the context, I'd prefer to see "because" used instead of "as" where appropriate. I have highlighted one example in the PDF but there are several. This is just a stylistic preference, but it can help readability and clarity.

DONE

In several places reference is made to "see below" or "as above" or similar. it would be better to refer to the specific relevant section in each case.

DONE

There are some long sentences that are difficult to follow. I have suggested splitting these into multiple shorter sentences

DONE

The LAMP anneal derivative temperature is sometimes referred to as just "anneal derivative" - make consistent (including "temperature" makes most sense)

DONE

I have not reviewed figures because they are not visible within this submission or via the reviewer log in

Noted

Executive summary

Lines 28 and 39 - I would prefer the authors use a term such as demonstrated/demonstrating or supported/supporting rather than confirmed/confirming when referring to the assay specificity. While the level of validation reported here is acceptable, I'd be hesitant to say that specificity is "confirmed" without much wider application of the assay

DONE. Changed as suggested.

Line 29 - 'specific' doesn't appear to make sense here - should this be 'sensitive'?

NOT DONE. For LAMP assays it is common practice to refer to testing an assay against a species-panel as “specificity testing”, whilst testing against known DNA concentrations (e.g. a dilution series) to determine the limits of detection is referred to as “sensitivity testing”. We have used these terms throughout this paper.

Line 40 - Sentence starting "While highly effective," - This reads as though degradation was effective. You could just leave this out, otherwise I'd suggest saying "While the assay was highly effective,"

DONE. Changed as suggested.

Introduction

Line 49 - repetition of "morphological"

DONE. Deleted as suggested.

Line 115 - suggest inserting "potentially" before "outperforming". Molecular methods weren't formally compared with traditional methods here, so this is speculative.

DONE. Added as suggested.

Line 124-125 - unnecessary repetition of "AHG".

DONE. Replaced 2 x “AHG” with “it”.

Line 136 "see below" - add specific reference

DONE. This note is not referring to other studies, but refers to the present work, where further details of the collection sites for our study are provided. Specific reference to the relevant section has been added (as comment above).

M&M

Line 153 - protocol should be protocols

DONE. Changed as suggested.

Line 156 - "remaining fraction" may be a better term rather than "other half" (given the fraction was 2/3 for some samples)

DONE. Changed as suggested.

Lines 156, 164 - You may need to define what you mean by destructive (vs non-destructive as used below) in this context. In my experience, "destructive" is when the organism is destroyed to provide the DNA sample, so I wouldn't think of scat

extractions as being "destructive", but there may be different definitions of the term. I'm also unclear how this destructive sampling (line 156) is different from the non-destructive sampling in the next section (line 164).

DONE. Added definitions, at first mention in text. “Destructive extraction (i.e., involving complete sample homogenisation)”; “Non-destructive extraction (i.e., not involving complete sample homogenisation)”.

Paragraph starting line 178 - Similarly to comment above, I think this needs a bit more context in terms of what you mean by "non-destructive" - e.g. do you mean the samples were (or replicated?) tissue naturally shed from the animal? Saying "in-field" when you were using museum samples also seems inconsistent/confusing. I assume you mean you used museum samples to simulate in-field extraction? Suggest re-wording to clarify.

DONE. As in above comment (i.e., terms are now defined in text). “Non-destructive” extraction applies here as well as the tissue samples were not homogenised.

Line 188 - Sentence starting "Most AHG" is very long - suggest splitting into 2-3 sentences

DONE. Changed as suggested.

Line 196-197 - put as "Australian Genome Research Facility (AGRF)"

DONE. Changed as suggested.

Line 212 - "comprise" may be a better term than "include" here

DONE. Changed as suggested.

Line 223 - the abbreviations used for the primer pairs should be defined. I'd suggest doing this within section 2.4.1 (suggest after sentence finishing at line 217). but it could be done at the start of section 2.4.2 or even in the intro if preferred. Currently this information is presented in the results (section 3.1) but would be better placed in the methods.

DONE. Changed as suggested. Added primer definitions here and removed from section 3.1.

Line 239 - as above, define F2 and B2

DONE. Changed as suggested. Added a reference to “FIP and BIP being comprised of two primer regions, F1+F2 and B1+B2 respectively” to the above definitions.

Line 247 - use "was" instead of "being" to make this sentence grammatically correct

DONE. Changed as suggested.

Line 259-260 - reads as "copy number...was prepared" Suggest you mean something like "copy number was determined, and..."

DONE. Changed as suggested.

Line 262 - add specific reference

DONE. “… conditions as mentioned above…” refers to the present study, not a previously published study. Specific reference to the relevant section has been added (as comment above).

Line 262-263 - The term "run time" appears to have a different meaning here from that in line 247 leading to some confusion about what is meant. Suggest rewording to clarify.

DONE. Changed as suggested. Have deleted 1 x “run time” in text, and changed another to “amplification time”, for clarity. “Run time” is now only used to refer to the complete LAMP assay amplification + annealing time.

Line 272 - remove "for"

DONE. Changed as suggested.

Line 281, 293, 305 - add specific references

DONE. As above, these sentences all provide directions to refer to other sections of the present study, not a previously published study. Specific reference to the relevant section has been added (as comment above).

Line 304-305 - suggest "and" in place of second instance of "using" to avoid repetition

DONE. Changed as suggested.

Results

Line 311-313 - this sentence provides a definition of the primers that is needed (and better placed) in the methods

DONE. Changed as suggested (see above comment).

Line 326 - reference to Table 2 here implies that Table 2 shows barcoding results, but rather it shows the primers that were used - the cross-reference needs clarification.

DONE. Deleted “Table 2”, making it clear that Table 3 (and Fig 2) provide the DNA barcoding results. have also added a reference to “S1 Supplementary Table” here, as it also includes DNA barcoding identifications.

Line 367 - Remove "Overall"

DONE. Changed as suggested.

Line 369 - "temperature" rather than "profile"

DONE. Changed as suggested.

Line 371 - suggest "and" in place of second instance of "confirming"

DONE. Changed as suggested.

Line 380 - "at" instead of "as"

DONE. Changed as suggested.

Line 390 - "by LAMP" rather than "through LAMP" is probably better terminology

DONE. Changed as suggested.

Line 405 - first sentence in section 3.5. This sentence needs rewording. Firstly it reads as "the sensitivity ... was quite sensitive" -suggest "quite high" instead. Also ", detecting" isn't grammatically correct here - suggest ", with as low as...copies/uL detected within 15 minutes"

DONE. Changed as suggested.

Line 407 - generally you refer to copies per uL so spelling out the number of copies here is redundant (if included, put as 100,000 not as text)

DONE. Changed as suggested.

Line 410-411 - sentence starting "The amplification time" currently reads as "the amplification time ... amplified in 8 minutes" - needs rewording to be grammatically correct. Also "starting DNA" is unclear here - do you mean the highest DNA concentration within the dilution series? Neat DNA concentration prior to dilution?

DONE. Changed as suggested.

Line 416-417 - suggest remove second (redundant) use of "LAMP" in this sentence

DONE. Changed as suggested.

Line 417 - Suggest "Across" rather than "In"

DONE. Changed as suggested.

Line 420 - Suggest rewording for better clarity readability, e.g. "... (NTC) did not amplify in any of the 18 runs, "

DONE. Changed as suggested.

Line 423 - I don't think you have enough evidence to "prove" robustness of the assay yet - suggest "demonstrating" as a better term.

DONE. Changed as suggested.

Line 431 - "The" instead of "This"

DONE. Changed as suggested.

Line 432-434 - Sentence starting "DNA dilutions" reads as "DNA dilutions ... were less sensitive" which doesn't make sense. The sentence is also not grammatically

correct - needs to be reworded. Suggested rewording is provided on the attached PDF

DONE. Changed as suggested in the PDF.

Discussion

Line 452 - suggest adding "from other groups" at the end of the sentence

DONE. Changed as suggested.

Line 453 - "and" instead of "which"

DONE. Changed as suggested.

Line 456 - suggest "yielded" rather than "possessed" as a better term here

DONE. Changed as suggested.

Line 469-470 - suggest rewording to avoid repetition

DONE. Changed as suggested.

Line 472 - "rate" is probably a better term than "speed" here

DONE. Changed as suggested.

Line 495 - remove "size"

DONE. Changed to “amount” for clarity.

Reviewer #4: This is the first time I reviewed the manuscript although it has already been under one circle of review. Unfortunately, I was not provided with the Figures and I could not provide any comments on their quality.

Noted

I have seen that both reviewers have provided comments regarding the structure of the Introduction. It is obvious that both reviewers have different writing styles, yet they are right. The Introduction and its paragraphs are not nicely connected to each other. It reads as if they were written separately and placed into the introduction. Although I do not have many suggestions on the paragraphs themselves, the nice flow between them is missing. For example, you describe nicely NGS for scats on the 2nd paragraph and all of the sudden you move to LAMP on the 3rd paragraph. What if you mention at the beginning of the 3rd paragraph something along the lines of “for NGS sampling and samples with low quality DNA, we need to use new sensitive methods that are effective and easy to use in the field. This is LAMP”.

DONE. Linking sentences between paragraphs have been added to the introduction, and other parts of the introduction have been rearranged to improve flow between paragraphs. Additionally, the use of gBlocks as positive controls for LAMP has been moved to the Methods section, as it did not fit in well with the introduction.

There are a few issues with the population and phylogeography analyses of this study. The methodology of many of the outputs described in the Results, are not presented in the “materials and methods”.

DONE. Changed as suggested (see other comments).

Abstract

Lines 26-30: This section is a bit confusing as there are two sentences where the time for the detection of AHG DNA varies. Is the second time (15 mins) an average time whereas the 25 mins for very low DNA concentrations? You should provide clarifications.

DONE. Changed as suggested. The terms assay “sensitivity” and “specificity” are used here again (see comment above) to differentiate between detection thresholds, and species-specificity, respectively.

Introduction

Line 52: Replace “e.g.” with “e.g.,”. Check this issue throughout the manuscript.

DONE. Changed as suggested.

Methods and Methods

Line 158: Was the PowerFecal® DNA kit not as successful as the QIAmp® Fast DNA Stool Mini kit? If that is the case, you should describe the results/comparison.

[OFFICIAL]

DONE. The reference to the preliminary work using the PowerFecal kit have been removed, as this kit was not used past initial testing.

Line 183: Leave a space between numbers and units. Check this issue throughout the manuscript.

DONE. Changed as suggested.

Line 188: ND2. This is the first time you use the term for the gene. Please provide the full name.

DONE. Changed as suggested.

Lines 188-197: Here you have used new primers, but you have not provided the complete PCR conditions, nor the volumes and concentrations of the used reagents.

DONE. Have clarified that the PCR conditions were as per the previously published study, apart from the annealing temperature being reduced to 56oC.

Lines 199-201: How were the raw sequences processed? There is no initial information about it and the authors go straight to describe how the obtained a ML tree.

DONE. Added extra information about how sequences were processed.

Line 204: What do the authors mean with “worldwide sequences”? Are they referring to sequences of specimens from around the world or the species’ distribution? Please clarify.

DONE. Changed as suggested.

Line 286: Section 2.6 is missing.

DONE. Corrected labelling of sections.

Results

Lines 311-313: You should rephrase this sentence as it reads as if you have 12 primers.

DONE. This information has been modified and moved to the methods (see above comment).

Lines 325-326: This is a repetition as the details, and the method has been described in the previous section.

DONE. This information has been modified (see above comment).

Lines 326-327: How was the haplotype network created? You have not described the details in the methods.

NOT DONE. Detailed information on how the haplotype network was constructed is already in the methods.

Line 331: “highly divergent lineage (>6% different),” how was this number generated?

DONE. This information has been added to the Methods.

Lines 332-334: This part should move to the discussion. This is not a result.

DONE. This has been changed as suggested.

Lines 347-349: Something is missing in this sentence.

DONE. This has been modified as suggested.

Lines 370-372: Please rephrase the sentence as the use of “confirming” is confusion.

DONE. This has been modified as suggested (see comment above).

Lines 405-407: “The detection sensitivity” ………… “was quite sensitive, detecting”. Please rephrase the sentence.

DONE. This has been modified as suggested (see comment above).

Lines 407-408: “one hundred thousand copies (1 x 105 copies/μL)” Keep only one. It is the same thing.

[OFFICIAL]

DONE. This information has been modified (see above comment).

Discussion

Line 450: Replace “Table” with “Tables”.

DONE. This has been changed as suggested.

Line 460: “36% different for the section” how did the authors come with this number?

DONE. This information has been added to the Methods (see above comment).

I have also seen that some of the previous reviewers’ comments have not been addressed. For example, the research permit for the samples from Indonesia. The ethics permits and “Cadaver and Tissue Usage” of one country is not the same when that involves samples collected at a different country. An ethics permit is not a research permit.

DONE. The preserved samples obtained from overseas were sourced directly from the Western Australian Museum, as stated in the Acknowledgments. The wording has been modified to make it clearer that samples were provided through the WAM, or an animal ethics cadaver and/or tissue notification form.

Additionally, the suggestion of a previous reviewer about “A comparative discussion with other available molecular tools (e.g., conventional PCR, qPCR, or other isothermal methods) would further highlight the advantages and limitations of the proposed technique, providing readers with a clearer perspective on its applicability.” has not been addressed. The reply that the authors provide in the text, does not address the suggestion. The discussion would actually benefit from it.

DONE. A discussion of other molecular approaches, including real-time PCR, compared with LAMP, for molecular diagnostics has been added to the Discussion.
