# Supplementary material for: A new molecular tool for detection of the highly invasive gecko, Hemidactylus frenatus
Source: PLoS One. 2026 Feb 4;21(2):e0338377. doi: 10.1371/journal.pone.0338377 (PMC12871987; doi:10.1371/journal.pone.0338377)
Supplement: Attachment — Submitted filename: PONE-D-25-13902_reviewer_notes.docx [file pone.0338377.s003.docx]

Reviews on *a new molecular tool for detecting the highly invasive gecko, Hemidactylus frenatus, were* submitted to *PLOS ONE*.

The LAMP assay for *H. frenatus* is a novel application of NGS for a vertebrate invader, building on prior work with invertebrates and predators (e.g., Refs [12, 19]). However, the manuscript could better articulate how this tool advances beyond existing methods (e.g., PCR-based scat barcoding) in cost, speed, or accessibility. The writing is clear, but technical details (e.g., primer design, scat DNA challenges) are dense and may obscure key findings for a broad audience. Simplifying or reorganizing some sections could enhance readability. The commitment to open data (GenBank accessions PQ390829-PQ390908) is commendable, aligning with *PLOS ONE*’s policy. Ensure all supporting data (e.g., S1 Table) are comprehensive and well-documented.

Lines 20-41: Add a sentence on limitations (e.g., scat DNA degradation) to balance the narrative, e.g., “While highly effective, scat DNA degradation limited detection in 21% of AHG samples, highlighting preservation challenges.”

Lines 66-87: The LAMP method’s advantages are well-explained, with relevant citations. However, the vertebrate example (red fox, Ref [19]) is underexplored—contrast its context (e.g., predator scat) with *H. frenatus* (small reptile scat) to justify this study’s novelty.

Lines 89-113: The ecological and biosecurity threat of *H. frenatus* is convincingly argued. The critique of auditory/visual surveys is valid but could quantify their limitations (e.g., detection rates from Ref [21]).

Lines 109-113: The comparison to PCR needs data—how does LAMP’s 1-hour turnaround compare to PCR’s 2-4 weeks in practice? Authors can explicitly state the hypothesis, e.g., “We hypothesized that a mitochondrial ND2 LAMP assay would enable rapid, specific detection of *H. frenatus* from scat DNA, outperforming traditional methods in field settings.”

Lines 125-131: Sample sourcing is transparent, but the rationale for selecting 25 non-target species is vague—were these chosen for morphological similarity, sympatry, or availability? Please clarify the text or Table 2 caption. Also, please state any research/ethics permit for collecting the samples and conducting the study.

Lines 140-153: The dual extraction approach (lab vs. field) is well-executed. However, scat size variation (half vs. one-third) introduces potential bias—did this affect DNA yield? Quantify or test this.

Lines 166-168 QE method optimization lacks justification for dilution choices (1:10, 1:20, 1:50)—why these ratios? Report initial trials or cite a precedent.

Lines 192: ND2 barcoding confirms species identity, but the haplotype network’s purpose is unclear until Results. Move its description to Section 3.2 for coherence.

Lines 201-209: Primer design is detailed, but the choice of ND2 over other loci (e.g., COI) isn’t justified—explain its variability or conservation in *H. frenatus*.

Lines 212-217: The 1:6:3 primer ratio is optimized, but raw optimization data are missing. Please include them in the S1 Table or justify them via Ref [17].

Lines 250-252: The gBlock evaluation is robust, but the 35-minute run time contradicts the abstract’s 25-minute claim. Conciliate this discrepancy.

Lines 265-267: Sensitivity testing is thorough, but scat vs. tissue differences (line 267) need statistical analysis (e.g., t-test on amplification times) to confirm significance.

Lines 285-286: Field validation is strong, but the switch from loops to pipettes in lab testing (line 285) introduces a variable—did this affect results? Test or discuss. Even if minimal, add a subsection on statistical methods to clarify data analysis (e.g., means ± SD).

Lines 294-305: Primer optimization is concise, but Figure 1’s alignment is redundant without sequence variation data—simplify or move to S1 Table.

Lines 308-326: The haplotype network (Figure 3) reveals a new Cocos Island lineage (“Group E”), a significant finding. Highlight its biosecurity implications (e.g., undetected invasion risk). The ML tree (Figure 2) is cluttered. Reduce non-target taxa to key comparators.

Lines 337: Please clarify the sentences. What did you suggest of the use of 1:1 dilution?

Lines 374-376: Non-target specificity is convincing, but Barrow Island’s 49 negatives could be a false adverse risk—discuss assay limits in Discussion.

Lines 405-423: Sensitivity differences (tissue vs. scat) are striking (22 vs. 14 minutes at low concentrations)—quantify DNA degradation or inhibitor effects to explain this in the discussion.

Lines 425-437: The assay’s success across *H. frenatus* diversity is a key strength. However, the lack of congeneric testing (e.g., *H. platyurus*) is a gap—acknowledge as a limitation or test if feasible.

Lines 438-448: gBlock utility is well-argued, but scat preservation advice is generic tailored to *H. frenatus* (e.g., arid vs. humid climates). Please explain more.

Lines 450-456: Contamination precautions are practical but overly essential—elevate with LAMP-specific risks (e.g., amplicon carryover).

Lines 464-467: Sensitivity analysis is insightful, but the scat size effect needs data—cross-reference S1 Table or test experimentally.

Lines 473-474: The 79% scat detection rate is contextualized, but verification advice is vague—specify barcoding turnaround time vs. LAMP’s speed.

Lines 479-490: The Barrow Island case study is a highlight—quantify cost/time savings to strengthen impact claims.

Lines 492-501: Livestock applications are speculative—focus on *H. frenatus* or cite LAMP disease studies to justify extrapolation. Add a paragraph on ecological implications (e.g., *H. frenatus* displacement of native geckos) to tie back to biosecurity goals.

Check again the relevance of each reference you sited in this manuscript to the study; e.g., [31] for ND2 sequences are heavily relied upon—diversify with other *H. frenatus* genetic studies if available.
