# Supplementary material for: A new molecular tool for detection of the highly invasive gecko, Hemidactylus frenatus
Source: PLoS One. 2026 Feb 4;21(2):e0338377. doi: 10.1371/journal.pone.0338377 (PMC12871987; doi:10.1371/journal.pone.0338377)
Supplement: Attachment — Submitted filename: ResponseToReviewers_PONE-D-25-13902_R1.docx [file pone.0338377.s004.docx]

**Response to Reviewers**

***Journal Requirements:***

When submitting your revision, we need you to address these additional requirements.

1. Please ensure that your manuscript meets PLOS ONE's style requirements, including those for file naming. The PLOS ONE style templates can be found at

https://journals.plos.org/plosone/s/file?id=wjVg/PLOSOne_formatting_sample_main_body.pdf and

https://journals.plos.org/plosone/s/file?id=ba62/PLOSOne_formatting_sample_title_authors_affiliations.pdf

**DONE.**

2. In the online submission form, you indicated that your data is available only on request from a third party. Please note that your Data Availability Statement is currently missing the name contact details for the third party, such as an email address or a link to where data requests can be made. Please update your statement with the missing information.

**DONE. All data has been supplied in the manuscript, the Supplementary files, & GenBank.**

3. Please include captions for your Supporting Information files at the end of your manuscript, and update any in-text citations to match accordingly. Please see our Supporting Information guidelines for more information: http://journals.plos.org/plosone/s/supporting-information.

**DONE. “S1 Supplementary Table” legend is included at end of manuscript. An additional “S2 Supplementary Data” file has now been added. This additional file includes the T-test data (tissue verses scat comparison) as well as the scat DNA optimisation results (portion extracted and dilution comparisons), as noted below.**

4. Please review your reference list to ensure that it is complete and correct. If you have cited papers that have been retracted, please include the rationale for doing so in the manuscript text, or remove these references and replace them with relevant current references. Any changes to the reference list should be mentioned in the rebuttal letter that accompanies your revised manuscript. If you need to cite a retracted article, indicate the article’s retracted status in the References list and also include a citation and full reference for the retraction notice.

**DONE.**

***Additional Editor Comments:***

1. Please ensure that table numbers are updated to match their order of appearance in the text.

**DONE. Table 1 and Table 2 have been renumbered to match the order in the text.**

2. Table 2, please include the abbreviations for Helix and WAM in the table legend, as all necessary information for interpreting the table should be provided.

**DONE. Added to legend (Table 1).**

3. Table 2, please include numbering for each row to facilitate the identification of the 26 AHG tissue samples and the 54 tissue samples from other gecko and skink species.

**DONE. Note, these numbers are also now used in the text to refer to non-target lizards (i.e., samples 27 to 80, Table 1).**

4. Please include an approximate weight for the half and one-third portions of scat used in the DNA extraction.

**DONE. Scats were not weighed for this study. Scat weight would depend heavily on the water content of scats. Instead, an indication of scat size has been added to the legend in Figure 4 (1/3 scat = approx. 3 x 3 mm, ½ scat = approx. 3x 5 mm).**

5. Please review the title of the last column in Table 1, as the text is overlapping and unreadable (Degener...)

**DONE. All data is present in the Table. The text issue appears to have occurred during PDF generation.**

6. In Discussion section, include limitations of the study.

**DONE. Additional discussion of limitations has been added (as outlined in the specific comments below).**

7. In Discussion section, compare your results to other published articles.

**DONE. References have been added throughout the Discussion.**

***Reviewer #1 Comments:***

The manuscript is technically sound, with experiments conducted rigorously using established molecular techniques, appropriate controls (gBlock, NTCs, non-target species), sufficient replication (multiple samples/dilutions), and adequate sample sizes for its aims. The data strongly support the conclusions, with clear evidence of the assay’s performance and application. Minor weaknesses—variable scat sizes, lack of statistical tests, and untested congeners—do not undermine the study’s validity but suggest areas for refinement. The conclusions are appropriately drawn, with limitations acknowledged, ensuring scientific integrity.

The descriptive approach is partially appropriate given the study’s focus on tool development and validation. The assay’s performance is convincingly demonstrated through means, SDs, and success rates, aligning with the objective of establishing functionality. However, the lack of inferential statistics limits the ability to rigorously confirm observed differences (e.g., tissue vs. scat sensitivity), which could strengthen claims about assay robustness.

The statistical analysis is technically appropriate but not rigorously applied. The descriptive statistics (means ± SD, percentages) effectively support the assay’s sensitivity (0.0001 ng/μL), specificity (100% non-target exclusion), and field utility (79% scat detection), aligning with the study’s goals. However, the absence of inferential statistics limits the ability to statistically validate observed differences, reducing rigor. For PLOS ONE, where methodological soundness is key, the current approach is sufficient for a methods paper, as the data’s quality and presentation carry the conclusions. Adding basic tests (e.g., t-tests) would enhance rigor without altering the study’s core findings. The analysis is adequate but could be improved with minimal statistical tests to confirm key trends, particularly for sensitivity and field performance comparisons.

The authors have made all data underlying their findings fully available, as required by PLOS ONE. The combination of: (1) Detailed tables (1-3) and figures (1-6) in the manuscript; (2) A comprehensive S1 Table (assumed to include raw field data); (3) Publicly accessible ND2 sequences in GenBank (PQ390829-PQ390908), (4) meets the policy’s standards for transparency and replicability.

Minor gaps (e.g., raw dilution times, scat metadata) are likely addressed in S1 Table, though confirmation requires reviewing the file. If S1 Table lacks these specifics, a slight revision (e.g., adding a supplementary table with dilution and scat details) would ensure completeness. The data availability is robust and compliant, with all critical findings supported by accessible data. Any small omissions are non-critical and can be easily rectified if needed.

I recommend acceptance with minor revisions. The study is methodologically sound, with significant practical value for biosecurity. To enhance its impact and meet PLOS ONE’s standards, please address the following:

(a) Clarify scat DNA limitations (e.g., 21% failure rate) in the abstract and Discussion.

(b) Justify methodological choices (e.g., ND2, scat size, dilutions) with data or citations.

(c) Quantify assay advantages over PCR (e.g., time, cost) in the Introduction and Discussion.

(d) Streamline figures (e.g., Figure 2) and ensure consistency (e.g., 25 vs. 35 minutes).

(e) Highlight the Cocos Island “Group E” finding as a novel contribution.

These revisions will strengthen the manuscript’s clarity, rigor, and appeal to a broad readership.

**DONE. The above suggestions are covered by the specific changes listed above and below.**

***Reviewer Comments:***

The LAMP assay for *H. frenatus* is a novel application of NGS for a vertebrate invader, building on prior work with invertebrates and predators (e.g., Refs [12, 19]). However, the manuscript could better articulate how this tool advances beyond existing methods (e.g., PCR-based scat barcoding) in cost, speed, or accessibility. The writing is clear, but technical details (e.g., primer design, scat DNA challenges) are dense and may obscure key findings for a broad audience. Simplifying or reorganizing some sections could enhance readability. The commitment to open data (GenBank accessions PQ390829-PQ390908) is commendable, aligning with *PLOS ONE*’s policy. Ensure all supporting data (e.g., S1 Table) are comprehensive and well-documented.

Lines 20-41: Add a sentence on limitations (e.g., scat DNA degradation) to balance the narrative, e.g., “While highly effective, scat DNA degradation limited detection in 21% of AHG samples, highlighting preservation challenges.”

**DONE. Note, also added a reference to DNA inhibitors here (see comment below).**

Lines 66-87: The LAMP method’s advantages are well-explained, with relevant citations. However, the vertebrate example (red fox, Ref [19]) is underexplored—contrast its context (e.g., predator scat) with *H. frenatus* (small reptile scat) to justify this study’s novelty.

**DONE. Text has been updated.**

Lines 89-113: The ecological and biosecurity threat of *H. frenatus* is convincingly argued. The critique of auditory/visual surveys is valid but could quantify their limitations (e.g., detection rates from Ref [21]).

**DONE. Additional details added regarding the limitation of using auditory signals as they are only commonly made under very specific conditions.**

Lines 109-113: The comparison to PCR needs data—how does LAMP’s 1-hour turnaround compare to PCR’s 2-4 weeks in practice? Authors can explicitly state the hypothesis, e.g., “We hypothesized that a mitochondrial ND2 LAMP assay would enable rapid, specific detection of H. frenatus from scat DNA, outperforming traditional methods in field settings.”

**DONE. Added as suggested.**

Lines 125-131: Sample sourcing is transparent, but the rationale for selecting 25 non-target species is vague—were these chosen for morphological similarity, sympatry, or availability? Please clarify the text or Table 2 caption.

**DONE. Clarified that these species of lizard co-occur in Western Australia. Added to the text.**

Also, please state any research/ethics permit for collecting the samples and conducting the study.

**DONE. Extra ethics details have been provided in the Acknowledgments.**

Lines 140-153: The dual extraction approach (lab vs. field) is well-executed. However, scat size variation (half vs. one-third) introduces potential bias—did this affect DNA yield? Quantify or test this.

**DONE. This issue is covered in the Discussion, with partial scats (1/2 or 1/3) amplifying more reliably than whole scats (with experimental optimisation data now provided in file S2). (Note, covered in the next point below regarding dilution reducing inhibitors shows this as well).**

Lines 166-168 QE method optimization lacks justification for dilution choices (1:10, 1:20, 1:50)—why these ratios? Report initial trials or cite a precedent.

**DONE. This section has been reworded to reflect these dilutions were performed as an experiment to trial reducing possible inhibitors** **through a series of increasing dilutions of DNA with water.**

Lines 192: ND2 barcoding confirms species identity, but the haplotype network’s purpose is unclear until Results. Move its description to Section 3.2 for coherence.

**DONE. Reworded to include an introductory sentence, but has not been moved, as this section describes the analysis method, while Section 3.2 describes the results of the analysis.**

Lines 201-209: Primer design is detailed, but the choice of ND2 over other loci (e.g., COI) isn’t justified—explain its variability or conservation in H. frenatus.

**DONE. Additional text has been included to state that ND2 is commonly used for gecko DNA barcoding. ND2 is well characterised in AHG, which is known to include multiple genetically distinct clades, and that ND2 has also been examined in many closely related species.**

Lines 212-217: The 1:6:3 primer ratio is optimized, but raw optimization data are missing. Please include them in the S1 Table or justify them via Ref [17].

**DONE. Added additional text to explain we tested multiple primer ratios as in the reference.**

Lines 250-252: The gBlock evaluation is robust, but the 35-minute run time contradicts the abstract’s 25-minute claim. Conciliate this discrepancy.

**DONE. Added additional text to explain the longer amplification time (35 min) allows detection of gBlock at very low concentrations.**

Lines 265-267: Sensitivity testing is thorough, but scat vs. tissue differences (line 267) need statistical analysis (e.g., t-test on amplification times) to confirm significance.

**DONE. T-tests in the Methods (here) and in the Results have been added to the manuscript (data in file S2), with the results are significant for time differences between tissue and scats, as expected.**

Lines 285-286: Field validation is strong, but the switch from loops to pipettes in lab testing (line 285) introduces a variable—did this affect results? Test or discuss. Even if minimal, add a subsection on statistical methods to clarify data analysis (e.g., means ± SD).

**DONE. Possible differences between using loops and pipettes was not tested statistically in the current study. However, additional text and a citation to the effectiveness of using loops in the field [reference 17] has now been added.**

Lines 294-305: Primer optimization is concise, but Figure 1’s alignment is redundant without sequence variation data—simplify or move to S1 Table.

**NOT CHANGED. Figure 1 does currently show sequence variation, both within AHG (Groups A to D) and the other species of gecko tested in our study. We feel it is useful to show where primer sites are located in relation to this sequence variation, so we believe it is valuable to retain this Figure. The Figure legend has been edited to make this clearer.**

Lines 308-326: The haplotype network (Figure 3) reveals a new Cocos Island lineage (“Group E”), a significant finding. Highlight its biosecurity implications (e.g., undetected invasion risk). The ML tree (Figure 2) is cluttered. Reduce non-target taxa to key comparators.

**DONE. Added text, highlighting that the presence of two highly divergent genetic lineages suggests that there have been at least two introduction events to the Cocos Islands. Our finding of “Clade E” is now also referred to in the Abstract and Discussion.**

**NOT CHANGED. All specimens / species on Figure 2 were experimentally tested using the new LAMP assay, so we feel that they should all be included on the ML tree.**

Lines 337: Please clarify the sentences. What did you suggest of the use of 1:1 dilution?

**DONE. Text modified.**

Lines 374-376: Non-target specificity is convincing, but Barrow Island’s 49 negatives could be a false adverse risk—discuss assay limits in Discussion.

**DONE. We discuss the importance for Barrow Island biosecurity in the Discussion. The risk of AHG being introduced to Barrow Island is relatively high, as demonstrated by multiple intercepted AHG in Table 1 / Figure 3, as well as the final case study which includes AHG being intercepted and identified through LAMP as it was in transit to the island.**

Lines 405-423: Sensitivity differences (tissue vs. scat) are striking (22 vs. 14 minutes at low concentrations)—quantify DNA degradation or inhibitor effects to explain this in the discussion.

**DONE. Text has been modified, tissue verse scats in the Discussion, to add scat degraded DNA and inhibitors. T-tests have now also been performed to show a significant difference between DNA obtained from tissue and scats (see comments above).**

Lines 425-437: The assay’s success across H. frenatus diversity is a key strength. However, the lack of congeneric testing (e.g., H. platyurus) is a gap—acknowledge as a limitation or test if feasible.

**DONE. Text has been modified and rearranged. Unfortunately, no congeneric samples were available for our experimental testing.**

Lines 438-448: gBlock utility is well-argued, but scat preservation advice is generic tailored to H. frenatus (e.g., arid vs. humid climates). Please explain more.

**DONE. The text has been modified.**

Lines 450-456: Contamination precautions are practical but overly essential—elevate with LAMP-specific risks (e.g., amplicon carryover).

**DONE. Text has been modified, with LAMP-specific risks now added, such as carryover if tubes are opened post-amplification.**

Lines 464-467: Sensitivity analysis is insightful, but the scat size effect needs data—cross-reference S1 Table or test experimentally.

**DONE. Scat extraction optimisation data has now been included in S2 file.**

Lines 473-474: The 79% scat detection rate is contextualized, but verification advice is vague—specify barcoding turnaround time vs. LAMP’s speed.

**DONE. Text has been modified to refer to the turnaround time of each approach.**

Lines 479-490: The Barrow Island case study is a highlight—quantify cost/time savings to strengthen impact claims.

**DONE. Text has been modified, to highlight the rapid accurate diagnostic results provided by LAMP in this case study.**

Lines 492-501: Livestock applications are speculative—focus on H. frenatus or cite LAMP disease studies to justify extrapolation. Add a paragraph on ecological implications (e.g., H. frenatus displacement of native geckos) to tie back to biosecurity goals.

**DONE. The last ‘livestock’ paragraph has been completely removed.**

Check again the relevance of each reference you cited in this manuscript to the study; e.g., [31] for ND2 sequences are heavily relied upon—diversify with other H. frenatus genetic studies if available.

**DONE. References have been checked and modified.**
