# Supplementary material for: A new molecular tool for detection of the highly invasive gecko, Hemidactylus frenatus
Source: PLoS One. 2026 Feb 4;21(2):e0338377. doi: 10.1371/journal.pone.0338377 (PMC12871987; doi:10.1371/journal.pone.0338377)
Supplement: Attachment — Submitted filename: REVIEW NOTES.docx [file pone.0338377.s005.docx]

REVIEW NOTES

The revised manuscript "A new molecular tool for detection of the highly invasive gecko, Hemidactylus frenatus" (PONE-D-25-13902R1) has undergone significant improvements since its initial review, addressing many of the concerns raised previously. It presents a novel LAMP assay for detecting the invasive Asian House Gecko (AHG) using non-invasive genetic sampling, a topic well-aligned with PLOS ONE’s scope in applied molecular ecology and biosecurity. The study demonstrates scientific rigor through its experimental design, data presentation, and practical application, making it a strong candidate for publication. However, while the revisions have enhanced clarity and depth, some areas still require refinement to fully meet PLOS ONE’s standards for clarity, methodological justification, and comprehensive discussion. Below, I outline my assessment and provide specific reviews and questions for improvement.

Reviewer Comments:

[L.44-125] The authors need to rewrite the introduction more systematically and not jump around with topics in paragraphs. The author can begin with the common knowledge of molecular tools, then introduce LAMP, and subsequently explain why LAMP is important for detecting invasive species, particularly those that are difficult to sample. This will then be applied to Hemidactylus frenatus.

Also explain the studies so far that have been done about this topic, what/how your study answers the important gap of those studies. Therefore, you can clearly state your research aims.

[L. 284-293] I notice there were samples from Indonesia that have been tested for field testing and lab validation of field samples. Please provide the research permit for this particular work sample.
