# Supplementary material for: A new molecular tool for detection of the highly invasive gecko, Hemidactylus frenatus
Source: PLoS One. 2026 Feb 4;21(2):e0338377. doi: 10.1371/journal.pone.0338377 (PMC12871987; doi:10.1371/journal.pone.0338377)
Supplement: Attachment — Submitted filename: Response To Reviewers_R2.docx [file pone.0338377.s006.docx]

**Response to Reviewers (PONE-D-25-13902R1)**

***Reviewer #1:***

The revised manuscript "A new molecular tool for detection of the highly invasive gecko, *Hemidactylus frenatus*" (PONE-D-25-13902R1) has undergone significant improvements since its initial review, addressing many of the concerns raised previously. It presents a novel LAMP assay for detecting the invasive Asian House Gecko (AHG) using non-invasive genetic sampling, a topic well-aligned with PLOS ONE’s scope in applied molecular ecology and biosecurity. The study demonstrates scientific rigor through its experimental design, data presentation, and practical application, making it a strong candidate for publication. However, while the revisions have enhanced clarity and depth, some areas still require refinement to fully meet PLOS ONE’s standards for clarity, methodological justification, and comprehensive discussion. Below, I outline my assessment and provide specific reviews and questions for improvement.

**Reviewer Comments:**

[L.44-125] The authors need to rewrite the introduction more systematically and not jump around with topics in paragraphs. The author can begin with the common knowledge of molecular tools, then introduce LAMP, and subsequently explain why LAMP is important for detecting invasive species, particularly those that are difficult to sample. This will then be applied to *Hemidactylus frenatus*.

Also explain the studies so far that have been done about this topic, what/how your study answers the important gap of those studies. Therefore, you can clearly state your research aims.

***Response:* While we appreciate the reviewer’s suggestion, we feel that the introduction is already structured in a logical order for this study:**

- **First, we introduce non-invasive genetic sampling (NGS) for early detection from animal traces (e.g. from animal scats), and examples of the use of LAMP as a method that can successfully be used on NGS samples.**
- **We then introduce the invasive gecko, AHG, followed by current indirect methods for detection of AHG (auditory and DNA barcoding).**
- **Followed by the project’s primary aim of designing a new LAMP assay for use in identification of AHG from indirect trace samples.**

[L. 284-293] I notice there were samples from Indonesia that have been tested for field testing and lab validation of field samples. Please provide the research permit for this particular work sample.

***Response:* The Indonesian samples were collected under the same ethics permits and “Cadaver and Tissue Usage” form as already stated in the manuscript (see Acknowledgments section).**

***Reviewer #2:***

The manuscript presents a valuable study, with significant potential for advancing the

field. The research is well-designed and methodologically sound. The findings are

clearly presented and support the conclusions drawn. With minor improvements, this

manuscript will be an excellent contribution to the journal.

1. To enhance clarity and flow, consider restructuring the introduction by first

introducing the broader context of biological invasions and the ecological

significance of the studied species. This should be followed by a discussion of the

molecular aspects, ensuring a logical progression for readers.

***Response:* While we appreciate the reviewer’s suggestions (which are a different again from those suggested by Reviewer 1), we feel that the introduction is already structured in a logical order for this study (as outlined above).**

2. Line 169 requires supporting literature. Please provide appropriate citations to

strengthen the claim.

***Response:* A reference to using a toothpick for transferring samples has been added here.**

3. Line 146 The phrase "clean DNA" may not be scientifically precise. Consider

replacing it with a more standard term.

***Response:* This term has been replaced with “high-quality DNA” throughout.**

4. Line 328-329 The manuscript should briefly address whether any variation in

reaction efficiency was observed when loop primers were excluded.

***Response:* This was not experimentally tested. It is standard practice in LAMP assays to use loop primers to increase reaction efficiency, so they were included as part of the assay design.**

5. A comparative discussion with other available molecular tools (e.g., conventional

PCR, qPCR, or other isothermal methods) would further highlight the advantages

and limitations of the proposed technique, providing readers with a clearer

perspective on its applicability.

***Response:* DNA barcoding is the only currently available alternative molecular tool available for identification of AHG (i.e., there are no alternative qPCR assays etc). We have discussed the assay differences and applicability of DNA barcoding compared with LAMP for identification in the Discussion (lines, 504-512).**
