# Supplementary material for: A new molecular tool for detection of the highly invasive gecko, Hemidactylus frenatus
Source: PLoS One. 2026 Feb 4;21(2):e0338377. doi: 10.1371/journal.pone.0338377 (PMC12871987; doi:10.1371/journal.pone.0338377)
Supplement: Attachment — Submitted filename: Response To Reviewers_R3.docx [file pone.0338377.s008.docx]

**Response To Reviewers - PONE-D-25-13902R2**

**Reviewer #2:** The authors have addressed all the comments raised in the previous version of the manuscript. In the present state, the manuscript is acceptable for the publication.

Thank you.

**Reviewer #3:** This manuscript describes development of a novel tool for detection of an invasive species. The work is technically sound and worthy of publication. The manuscript is generally well written, but there are some corrections and clarifications needed to meet the standard for publication. I have provided comments and suggested changes on the attached PDF and summarise the recommendations here:

***General comments***

Check usage of commas, these are sometimes missing where appropriate to use and in other cases are included inappropriately. I have made suggested changes on the PDF but please check throughout

DONE

The term 'in-field' is sometimes spelled as "infield" which isn't the intended meaning - make sure "in-field" is used consistently

DONE

There are several sentences with repeated use of the same or very similar terms. While not strictly incorrect, these can be jarring to read, and I'd recommend rewording to avoid repetition where possible. I have commented on examples in the PDF

DONE

The word "as" is used a lot in the text, with various meanings. While its meaning is usually clear from the context, I'd prefer to see "because" used instead of "as" where appropriate. I have highlighted one example in the PDF but there are several. This is just a stylistic preference, but it can help readability and clarity.

DONE

In several places reference is made to "see below" or "as above" or similar. it would be better to refer to the specific relevant section in each case.

DONE

There are some long sentences that are difficult to follow. I have suggested splitting these into multiple shorter sentences

DONE

The LAMP anneal derivative temperature is sometimes referred to as just "anneal derivative" - make consistent (including "temperature" makes most sense)

DONE

I have not reviewed figures because they are not visible within this submission or via the reviewer log in

Noted

**Executive summary**

Lines 28 and 39 - I would prefer the authors use a term such as demonstrated/demonstrating or supported/supporting rather than confirmed/confirming when referring to the assay specificity. While the level of validation reported here is acceptable, I'd be hesitant to say that specificity is "confirmed" without much wider application of the assay

DONE. Changed as suggested.

Line 29 - 'specific' doesn't appear to make sense here - should this be 'sensitive'?

NOT DONE. For LAMP assays it is common practice to refer to testing an assay against a species-panel as “specificity testing”, whilst testing against known DNA concentrations (e.g. a dilution series) to determine the limits of detection is referred to as “sensitivity testing”. We have used these terms throughout this paper.

Line 40 - Sentence starting "While highly effective," - This reads as though degradation was effective. You could just leave this out, otherwise I'd suggest saying "While the assay was highly effective,"

DONE. Changed as suggested.

**Introduction**

Line 49 - repetition of "morphological"

DONE. Deleted as suggested.

Line 115 - suggest inserting "potentially" before "outperforming". Molecular methods weren't formally compared with traditional methods here, so this is speculative.

DONE. Added as suggested.

Line 124-125 - unnecessary repetition of "AHG".

DONE. Replaced 2 x “AHG” with “it”.

Line 136 "see below" - add specific reference

DONE. This note is not referring to other studies, but refers to the present work, where further details of the collection sites for our study are provided. Specific reference to the relevant section has been added (as comment above).

**M&M**

Line 153 - protocol should be protocols

DONE. Changed as suggested.

Line 156 - "remaining fraction" may be a better term rather than "other half" (given the fraction was 2/3 for some samples)

DONE. Changed as suggested.

Lines 156, 164 - You may need to define what you mean by destructive (vs non-destructive as used below) in this context. In my experience, "destructive" is when the organism is destroyed to provide the DNA sample, so I wouldn't think of scat extractions as being "destructive", but there may be different definitions of the term. I'm also unclear how this destructive sampling (line 156) is different from the non-destructive sampling in the next section (line 164).

DONE. Added definitions, at first mention in text. “Destructive extraction (i.e., involving complete sample homogenisation)”; “Non-destructive extraction (i.e., not involving complete sample homogenisation)”.

Paragraph starting line 178 - Similarly to comment above, I think this needs a bit more context in terms of what you mean by "non-destructive" - e.g. do you mean the samples were (or replicated?) tissue naturally shed from the animal? Saying "in-field" when you were using museum samples also seems inconsistent/confusing. I assume you mean you used museum samples to simulate in-field extraction? Suggest re-wording to clarify.

DONE. As in above comment (i.e., terms are now defined in text). “Non-destructive” extraction applies here as well as the tissue samples were not homogenised.

Line 188 - Sentence starting "Most AHG" is very long - suggest splitting into 2-3 sentences

DONE. Changed as suggested.

Line 196-197 - put as "Australian Genome Research Facility (AGRF)"

DONE. Changed as suggested.

Line 212 - "comprise" may be a better term than "include" here

DONE. Changed as suggested.

Line 223 - the abbreviations used for the primer pairs should be defined. I'd suggest doing this within section 2.4.1 (suggest after sentence finishing at line 217). but it could be done at the start of section 2.4.2 or even in the intro if preferred. Currently this information is presented in the results (section 3.1) but would be better placed in the methods.

DONE. Changed as suggested. Added primer definitions here and removed from section 3.1.

Line 239 - as above, define F2 and B2

DONE. Changed as suggested. Added a reference to “FIP and BIP being comprised of two primer regions, F1+F2 and B1+B2 respectively” to the above definitions.

Line 247 - use "was" instead of "being" to make this sentence grammatically correct

DONE. Changed as suggested.

Line 259-260 - reads as "copy number...was prepared" Suggest you mean something like "copy number was determined, and..."

DONE. Changed as suggested.

Line 262 - add specific reference

DONE. “… conditions as mentioned above…” refers to the present study, not a previously published study. Specific reference to the relevant section has been added (as comment above).

Line 262-263 - The term "run time" appears to have a different meaning here from that in line 247 leading to some confusion about what is meant. Suggest rewording to clarify.

DONE. Changed as suggested. Have deleted 1 x “run time” in text, and changed another to “amplification time”, for clarity. “Run time” is now only used to refer to the complete LAMP assay amplification + annealing time.

Line 272 - remove "for"

DONE. Changed as suggested.

Line 281, 293, 305 - add specific references

DONE. As above, these sentences all provide directions to refer to other sections of the present study, not a previously published study. Specific reference to the relevant section has been added (as comment above).

Line 304-305 - suggest "and" in place of second instance of "using" to avoid repetition

DONE. Changed as suggested.

**Results**

Line 311-313 - this sentence provides a definition of the primers that is needed (and better placed) in the methods

DONE. Changed as suggested (see above comment).

Line 326 - reference to Table 2 here implies that Table 2 shows barcoding results, but rather it shows the primers that were used - the cross-reference needs clarification.

DONE. Deleted “Table 2”, making it clear that Table 3 (and Fig 2) provide the DNA barcoding results. have also added a reference to “S1 Supplementary Table” here, as it also includes DNA barcoding identifications.

Line 367 - Remove "Overall"

DONE. Changed as suggested.

Line 369 - "temperature" rather than "profile"

DONE. Changed as suggested.

Line 371 - suggest "and" in place of second instance of "confirming"

DONE. Changed as suggested.

Line 380 - "at" instead of "as"

DONE. Changed as suggested.

Line 390 - "by LAMP" rather than "through LAMP" is probably better terminology

DONE. Changed as suggested.

Line 405 - first sentence in section 3.5. This sentence needs rewording. Firstly it reads as "the sensitivity ... was quite sensitive" -suggest "quite high" instead. Also ", detecting" isn't grammatically correct here - suggest ", with as low as...copies/uL detected within 15 minutes"

DONE. Changed as suggested.

Line 407 - generally you refer to copies per uL so spelling out the number of copies here is redundant (if included, put as 100,000 not as text)

DONE. Changed as suggested.

Line 410-411 - sentence starting "The amplification time" currently reads as "the amplification time ... amplified in 8 minutes" - needs rewording to be grammatically correct. Also "starting DNA" is unclear here - do you mean the highest DNA concentration within the dilution series? Neat DNA concentration prior to dilution?

DONE. Changed as suggested.

Line 416-417 - suggest remove second (redundant) use of "LAMP" in this sentence

DONE. Changed as suggested.

Line 417 - Suggest "Across" rather than "In"

DONE. Changed as suggested.

Line 420 - Suggest rewording for better clarity readability, e.g. "... (NTC) did not amplify in any of the 18 runs, "

DONE. Changed as suggested.

Line 423 - I don't think you have enough evidence to "prove" robustness of the assay yet - suggest "demonstrating" as a better term.

DONE. Changed as suggested.

Line 431 - "The" instead of "This"

DONE. Changed as suggested.

Line 432-434 - Sentence starting "DNA dilutions" reads as "DNA dilutions ... were less sensitive" which doesn't make sense. The sentence is also not grammatically correct - needs to be reworded. Suggested rewording is provided on the attached PDF

DONE. Changed as suggested in the PDF.

**Discussion**

Line 452 - suggest adding "from other groups" at the end of the sentence

DONE. Changed as suggested.

Line 453 - "and" instead of "which"

DONE. Changed as suggested.

Line 456 - suggest "yielded" rather than "possessed" as a better term here

DONE. Changed as suggested.

Line 469-470 - suggest rewording to avoid repetition

DONE. Changed as suggested.

Line 472 - "rate" is probably a better term than "speed" here

DONE. Changed as suggested.

Line 495 - remove "size"

DONE. Changed to “amount” for clarity.

**Reviewer #4:** This is the first time I reviewed the manuscript although it has already been under one circle of review. Unfortunately, I was not provided with the Figures and I could not provide any comments on their quality.

Noted

I have seen that both reviewers have provided comments regarding the structure of the Introduction. It is obvious that both reviewers have different writing styles, yet they are right. The Introduction and its paragraphs are not nicely connected to each other. It reads as if they were written separately and placed into the introduction. Although I do not have many suggestions on the paragraphs themselves, the nice flow between them is missing. For example, you describe nicely NGS for scats on the 2nd paragraph and all of the sudden you move to LAMP on the 3rd paragraph. What if you mention at the beginning of the 3rd paragraph something along the lines of “for NGS sampling and samples with low quality DNA, we need to use new sensitive methods that are effective and easy to use in the field. This is LAMP”.

DONE. Linking sentences between paragraphs have been added to the introduction, and other parts of the introduction have been rearranged to improve flow between paragraphs. Additionally, the use of gBlocks as positive controls for LAMP has been moved to the Methods section, as it did not fit in well with the introduction.

There are a few issues with the population and phylogeography analyses of this study. The methodology of many of the outputs described in the Results, are not presented in the “materials and methods”.

DONE. Changed as suggested (see other comments).

**Abstract**

Lines 26-30: This section is a bit confusing as there are two sentences where the time for the detection of AHG DNA varies. Is the second time (15 mins) an average time whereas the 25 mins for very low DNA concentrations? You should provide clarifications.

DONE. Changed as suggested. The terms assay “sensitivity” and “specificity” are used here again (see comment above) to differentiate between detection thresholds, and species-specificity, respectively.

**Introduction**

Line 52: Replace “e.g.” with “e.g.,”. Check this issue throughout the manuscript.

DONE. Changed as suggested.

**Methods and Methods**

Line 158: Was the PowerFecal® DNA kit not as successful as the QIAmp® Fast DNA Stool Mini kit? If that is the case, you should describe the results/comparison.

DONE. The reference to the preliminary work using the PowerFecal kit have been removed, as this kit was not used past initial testing.

Line 183: Leave a space between numbers and units. Check this issue throughout the manuscript.

DONE. Changed as suggested.

Line 188: ND2. This is the first time you use the term for the gene. Please provide the full name.

DONE. Changed as suggested.

Lines 188-197: Here you have used new primers, but you have not provided the complete PCR conditions, nor the volumes and concentrations of the used reagents.

DONE. Have clarified that the PCR conditions were as per the previously published study, apart from the annealing temperature being reduced to 56oC.

Lines 199-201: How were the raw sequences processed? There is no initial information about it and the authors go straight to describe how the obtained a ML tree.

DONE. Added extra information about how sequences were processed.

Line 204: What do the authors mean with “worldwide sequences”? Are they referring to sequences of specimens from around the world or the species’ distribution? Please clarify.

DONE. Changed as suggested.

Line 286: Section 2.6 is missing.

DONE. Corrected labelling of sections.

**Results**

Lines 311-313: You should rephrase this sentence as it reads as if you have 12 primers.

DONE. This information has been modified and moved to the methods (see above comment).

Lines 325-326: This is a repetition as the details, and the method has been described in the previous section.

DONE. This information has been modified (see above comment).

Lines 326-327: How was the haplotype network created? You have not described the details in the methods.

NOT DONE. Detailed information on how the haplotype network was constructed is already in the methods.

Line 331: “highly divergent lineage (>6% different),” how was this number generated?

DONE. This information has been added to the Methods.

Lines 332-334: This part should move to the discussion. This is not a result.

DONE. This has been changed as suggested.

Lines 347-349: Something is missing in this sentence.

DONE. This has been modified as suggested.

Lines 370-372: Please rephrase the sentence as the use of “confirming” is confusion.

DONE. This has been modified as suggested (see comment above).

Lines 405-407: “The detection sensitivity” ………… “was quite sensitive, detecting”. Please rephrase the sentence.

DONE. This has been modified as suggested (see comment above).

Lines 407-408: “one hundred thousand copies (1 x 105 copies/μL)” Keep only one. It is the same thing.

DONE. This information has been modified (see above comment).

**Discussion**

Line 450: Replace “Table” with “Tables”.

DONE. This has been changed as suggested.

Line 460: “36% different for the section” how did the authors come with this number?

DONE. This information has been added to the Methods (see above comment).

I have also seen that some of the previous reviewers’ comments have not been addressed. For example, the research permit for the samples from Indonesia. The ethics permits and “Cadaver and Tissue Usage” of one country is not the same when that involves samples collected at a different country. An ethics permit is not a research permit.

DONE. The preserved samples obtained from overseas were sourced directly from the Western Australian Museum, as stated in the Acknowledgments. The wording has been modified to make it clearer that samples were provided through the WAM, or an animal ethics cadaver and/or tissue notification form.

Additionally, the suggestion of a previous reviewer about “A comparative discussion with other available molecular tools (e.g., conventional PCR, qPCR, or other isothermal methods) would further highlight the advantages and limitations of the proposed technique, providing readers with a clearer perspective on its applicability.” has not been addressed. The reply that the authors provide in the text, does not address the suggestion. The discussion would actually benefit from it.

DONE. A discussion of other molecular approaches, including real-time PCR, compared with LAMP, for molecular diagnostics has been added to the Discussion.
